# General, Practical and Selective Oxidation Protocol for CF_3_S into CF_3_S(O) Group

**DOI:** 10.3390/molecules24071249

**Published:** 2019-03-30

**Authors:** Liubov V. Sokolenko, Raisa K. Orlova, Andrey A. Filatov, Yurii L. Yagupolskii, Emmanuel Magnier, Bruce Pégot, Patrick Diter

**Affiliations:** 1 Institute of Organic Chemistry, National Academy of Sciences of Ukraine, Murmans’ka Str. 5, 02094 Kyiv, Ukraine; sokolenko.liubov@gmail.com (L.V.S.); OrlovaRaisa@ukr.net (R.K.O.); filatov@ioch.kiev.ua (A.A.F.); 2 Bâtiment Lavoisier, Université de Versailles-Saint-Quentin, 45 avenue des Etats-Unis, 78035 Versailles, France; bruce.pegot@uvsq.fr (B.P.); patrick.diter@uvsq.fr (P.D.); 3 Enamine Ltd, A. Matrosova str. 23, Kiev 01103, Ukraine

**Keywords:** trifluoromethylsulfides, trifluoromethylsulfoxides, oxidation, trifluoroperacetic acid, dihydrogen peroxide

## Abstract

A simple and efficient protocol for the oxidation of trifluoromethyl, mono- and difluoromethyl sulfides to the corresponding sulfoxides without over-oxidation to sulfones, using TFPAA prepared in situ from trifluoroacetic acid and 15% H_2_O_2_ aqueous solution was developed. The methodology is suitable for a wide range of aromatic and aliphatic substrates in milligram and multigram scales.

## 1. Introduction

The synthesis of trifluoromethylsulfinyl containing compounds is a recent trend in current organic chemistry due to the practical significance of such compounds [1]. In particular, their biological activity is promising. They are also key intermediates for trifluoromethyl-containing sulfoximine synthesis and are excellent chiral auxiliaries [2,3,4,5]. Synthetic approaches dedicated to trifluoromethyl sulfoxides include (1) trifluoromethylation of the corresponding sulfinyl halides or sulfinic esters using TMSCF_3_ [6,7,8], (2) reaction of aromatic compounds with triflinate salts [9], (3) rearrangement of aryl triflinates in the presence of AlCl_3_ [10], (4) trifluoromethanesulfinilation using Langlois’ system (CF_3_SO_2_Na/POCl_3_) [11] or with 1-(trifluoromethylsulfinyl)-pyrrolidine-2,5-dione [12]. However, the most important and common method for the synthesis of trifluoromethylsulfoxides is the oxidation of the corresponding trifluoromethylsulfides using various oxidants, mainly peroxyacids, e.g. mCPBA [1]. It is noteworthy to mention that oxidation with mCPBA is sensitive to temperature. As a result, over-oxidation frequently occurs and non-negligible amounts of trifluoromethylsulfone are formed [13]. Furthermore, this reagent converts into m-chlorobenzoic acid, which is sometimes not easy to separate from the sulfoxide. Oxidation of an aliphatic sulfide was also described with Oxone^®^ as oxidant [14]. Waste by-products formed during the oxidation reaction as well as the necessity to use silica as a component of reaction medium cause difficulties in scaling up. Trifluoroperacetic acid (TFPAA) was shown to be a convenient reagent for the oxidation of sulfides to sulfoxides, as it reacts more rapidly at low temperature than other peroxy acids [15]. To the best of our knowledge, TFPAA has not been widely applied to the oxidation of perfluoroalkyl sulfides and only a few articles [14,16,17,18,19,20,21] and patents [22,23,24,25,26,27] documented this procedure. TFPAA solution can be prepared from 30% H_2_O_2_ and trifluoroacetic acid or 80–90% H_2_O_2_ and trifluoroacetic anhydride [28,29]. On the basis of literature data and our experience in oxidation reactions of trifluoromethyl sulfides, the use of TFPAA, obtained by both methods, has one common limitation as for mCPBA: over-oxidation readily occurs to form difficult-to-separate mixtures of trifluoromethyl sulfide, trifluoromethyl sulfoxide, and trifluoromethyl sulfone [17,18,19]. On the other hand, regarding the advantages of this method (easy to prepare and a cheap reagent, trifluoroacetic acid as side product), we believed there was an urgent need to exploit it. The description of a general and selective method of oxidation is especially important in the context of an exponential growth of molecules bearing a SCF_3_ group [1].

Herein we propose an easy-to-handle and high-yielding procedure for the oxidation of alkyl and aryl perfluoroalkyl sulfides to the corresponding sulfoxides without over-oxidation thanks to the reappraisal of oxidation by TFPAA.

## 2. Results and Discussion

**Scheme 1:**
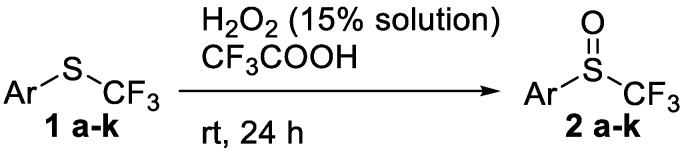
Oxidation of aryl trifluoromethyl sulfides **1**.

We postulated that a decrease in concentration, associated with a rigorous measurement of the latter, could dramatically reduce the formation of unwanted sulfone. Indeed, the use of TFPAA obtained in situ from trifluoroacetic acid and 15% H_2_O_2_ aqueous solution proved to be successful (see experimental part). This method was firstly applied to aryl trifluoromethyl sulfides **1** bearing both electrons donating and electron-withdrawing substituents in various positions on the aromatic ring (Scheme 1, Table 1). Corresponding aryl trifluoromethyl sulfoxides **2** were obtained in high yields whatever the nature of the substituents on the aromatic ring. In all experiments, the crude product contained less than 7% of non-reacted trifluoromethyl sulfide; at the same time, trifluoromethyl sulfone was detected in negligible amounts in only two reactions (Table 1, Entry 4, 10).

In most cases the crude product needs no further purification. If needed, compounds **2** were easily separated from impurities by standard methods (see experimental part for details). It should be noted that during the oxidation of acylated phenol **1i** partial hydrolysis occurred and the crude product contained ~15% of o-trifluoromethylsulfinyl phenol, which we failed to separate from **2i** by column chromatography.

**Scheme 2:**
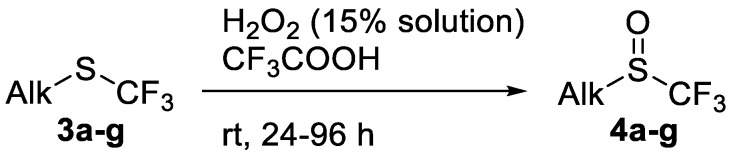
Oxidation of alkyl trifluoromethyl sulfides **3**.

Oxidation of aliphatic sulfides also proved successful (Scheme 2, Table 2).

The thioether **3a** was oxidized by TFPAA in high yield (83%) in 24 h. This simple procedure can be favorably compared to our previous method which employed Oxone^®^ [14]. Compound **4b** was isolated in low yield, most probably due to the high solubility of the product in water. Attempts to separate **4b** from **3b** by distillation failed. Use of an acyl protective group (Table 2, Entry 3) led to a higher yield of sulfoxide **4c**. In this case, as well as in the previous one, a small amount (<3%) of trifluoromethyl sulfone was detected in the reaction mixture and the crude product. As in the experiment with acylated phenol **1i** (Table 1, Entry 9), product **4c** contains ~15% of the corresponding alcohol (Table 2, Entry 3), which we failed to separate from the ester by distillation. Oxidation of sulfide **3d** by 15% H_2_O_2_ solution in TFA proceeded with 90% conversion. The use of H_2_O_2_ in excess (1.1 equiv.) led to the same results and the sulfone was not observed in this reaction mixture. Two other examples demonstrated the efficiency of this transformation (entries 6–7).

**Scheme 3:**
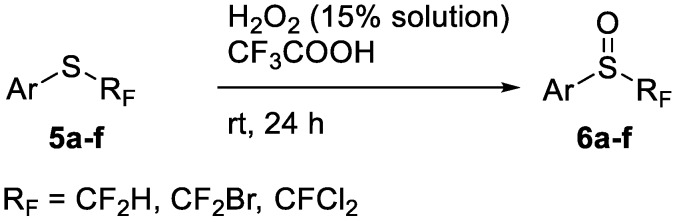
Oxidation of fluoromethyl and difluoromethyl sulfides **5**.

To show the scope of our oxidation procedure a number of sulfides bearing difluoromethyl **5a**–**c**, bromodifluoromethyl **5e**, dichlorofluoromethyl **5f**, and 1,1,2,2-tetrafluoro-2-((1,2,4-triazol)­-1-yl)-ethyl **5d** groups were converted into sulfoxides (Scheme 3,Table 3).

As it is evident from Table 3, all compounds **6a**–**f** were obtained in high yields and the crude products were pure enough to be used without further purification. The oxidation of **5a** was strongly exothermic and the reaction was complete within 4 h at room temperature yielding pure sulfoxide **6a**, which contained neither difluoromethyl sulfide **5a** nor difluoromethyl sulfone. If this reaction was performed at −15 °C for 6 h, with further stirring at ~10 °C for 20 h, the crude product contained ~2% of unreacted sulfide **5a**.

It should be emphasized that the oxidation reactions of sulfides **1**, **3**, and **5** could be performed on a 0.05 to 50 g scale.

**Scheme 4:**
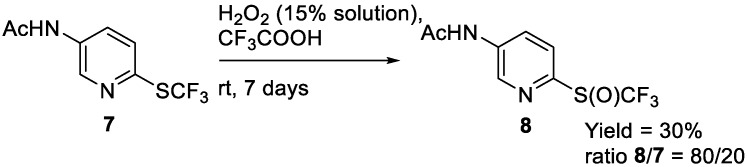
Oxidation of heterocyclic trifluoromethyl sulfides.

The application of this method to heterocyclic thioethers is highly challenging, as demonstrated by the two following preliminary results. Thus, 2-trifluoromethylsulfenyl-5-acetylamino-pyridine **7** was oxidized to sulfoxide **8** with low conversion after seven days stirring, and we failed to separate the product from the starting sulfide (Scheme 4). A study devoted to the oxidation of heterocyclic substrates is needed but falls outside the scope of the present study.

## 3. Materials and Methods

### 3.1. General Information

The purification of products by column chromatography (CC) was performed on Silica gel, 70–230 mesh 60A (Aldrich, Saint Louis, MI, USA) or by preparative TLC chromatography. ^1^H NMR spectra were recorded at 500 MHz with Bruker AVANCE DRX 500 instrument, or at 200 MHz or 300 MHz with Bruker AC-200 or AC-300, or at 400 MHz with Varian UNITY–Plus 400 spectrometer. ^19^F NMR spectra were recorded on Varian UNITY–Plus 400 spectrometer at 376.5 MHz or at 470 MHz with Bruker AVANCE DRX 500 instrument or at 188 MHz with Bruker AC-200 (Bruker, Billerica, MA, USA). Chemical shifts are given in ppm relative to Me_4_Si and CCl_3_F, respectively, as internal or external standards. ^13^C NMR-spectra (proton decoupled) were recorded on a Bruker AVANCE DRX 500 instrument at 125.7 MHz, or on Varian UNITY–Plus 400 spectrometer at 100.6 MHz, or at 75 or 50 MHz with Bruker AC-300 or AC-200. IR spectra were recorded with a Vertex 70 (Bruker) instrument (in KBr tablet). Melting points were determined in open capillaries using SMP3 instrument (Stuart Scientific Bibby Sterlin Ltd, Stone, Staffordshire, UK). Elemental analysis was performed in the Analytical Laboratory of the Institute of Organic Chemistry, NAS of Ukraine, Kyiv.

In all experiments trifluoroacetic acid from Sigma-Aldrich, 99% (CAS 76-05-1) was used. Difluoromethyl sulfides **5a**–**c** were synthesized by standard method [30]. Compound **5d** was synthesized as was described in the article [31]. Fluoromethylsulfides **1k**, **3f,g**, and **5e,f** were prepared according to the described procedures [32,33,34]. Trifluoromethylsulfides **1a**–**j**, **3a**–**e**, **7**, and **9** were purchased from Enamine Ltd (www.enamine.net).

### 3.2. Important Information

It is critical to know the exact concentration of H_2_O_2_ used in order to avoid over dosage of reagent that led to over oxidation. A solution of 15% H_2_O_2_ (mass concentration) was prepared from commercial ~30–35% H_2_O_2_ solution by dilution with water ~1:1 *w*/*w*. The concentration of H_2_O_2_ solution was measured using densimetry or titration (see Supplementary Materials).

### 3.3. General Procedure for Oxidation of Polyfluoroalkyl Sulfides to Polyfluoroalkyl Sulfoxides

To the solution of sulfide **1**, **3**, or **5** (10 mmol) in CF_3_COOH (15–20 mL) 15 mass% aqueous solution of H_2_O_2_ (containing 10 mmol of H_2_O_2_) was added dropwise very slowly (during 40–90 min) at room temperature. Reaction is strongly exothermic; H_2_O_2_ was added at such a rate that the temperature was kept in the range 25–28 °C inside the flask (20 °C for compounds **1k**, **3f,g**, and 0 °C for compounds **5e,f**). Reaction mixture was stirred overnight at r.t. (for compound **3d** − 96 h, for **7**–7 days), poured into water, neutralized with solid NaHCO_3_ to pH=6–7, then extracted with ether or ethyl acetate (4 × 30 mL). Compounds **2k**, **4f,g**, and **6e,f** were extracted with dichloromethane before neutralizing the TFA with NaHCO_3_. The organic phase was washed with water (4 × 20 mL), dried with MgSO_4_ or Na_2_SO_4_. Solvent was removed at atmospheric pressure for low-boiling liquids or on a rotary evaporator for solids or high-boiling liquids. Crude product was analyzed by NMR and purified if necessary.

*Phenyl Trifluoromethyl sulfoxide***2a** [13,35,36]. Colorless liquid; yield 1.8 g (95%). ^1^H NMR (CDCl_3_, 400 MHz): δ = 7.56–7.63 (m, 3H, Ar-H), 7.75–7.77 (m, 2H, Ar-H). ^19^F NMR (DMSO-*d_6_*, 376.5 MHz): δ = −76.6 (s). Compound **1a** was oxidized on a 50 g scale (in 70 mL of CF_3_COOH) with the same yield.

*N-(4-((Trifluoromethyl)sulfinyl)phenyl)acetamide***2b** [37,38]. Crude product was purified by column chromatography, eluent CH_2_Cl_2_ (R_f_ = 0), then methyl-tert-butyl ether (R_f_ = 0.6). Pale yellow solid; yield 2.1 g (85 %); m.p. 140–141 °C (Lit. 141–142 °C). ^1^H NMR (DMSO-*d_6_*, 500 MHz): δ = 2.10 (s, 3H, CH_3_), 7.80 (d, *^3^J_H-H_* = 8 Hz, 2H, Ar-H), 7.90 (d, *^3^J_H-H_* = 8 Hz, 2H, Ar-H), 10.41 (s, 1H, NH). ^19^F NMR (DMSO-*d_6_*, 376.5 MHz): δ = −75.2 (s). ^13^C NMR (DMSO-d_6_, 125.7 MHz): δ = 24.5, 119.8, 125.2 (q, *^1^J_C-F_* = 335.6 Hz, CF_3_), 127.6, 128.2, 144.8, 169.6. Compound **1b** was also oxidized on a 10 g scale (in 30 mL of CF_3_COOH) with the same yield.

*N-(3-((Trifluoromethyl)sulfinyl)phenyl)acetamide***2c** [37]. White solid; yield 2.4 g (96%); m.p. 101–102 °C (Lit. 104.5–105.5 °C). ^1^H NMR (DMSO-*d_6_*, 500 MHz): δ = 2.08 (s, 3H, CH_3_), 7.49 (d,*^3^J_H-H_* = 8 Hz, 1H, Ar-H), 7.61 (t, *^3^J_H-H_* = 8 Hz, 1H, Ar-H), 7.85 (d, *^3^J_H-H_* = 8 Hz, 1H, Ar-H), 8.24 (s, 1H, Ar-H), 10.38 (s, 1H, NH). ^19^F NMR (DMSO-*d_6_*, 376.5 MHz): δ = −74.8 (s). ^13^C NMR (DMSO-*d_6_*, 125.7 MHz): δ = 24.0, 115.1, 120.2, 123.6, 124.7 (q, *^1^J_C-F_* = 335.6 Hz, CF_3_), 130.2, 135.8, 140.7, 168.9.

*N-(2-((Trifluoromethyl)sulfinyl)phenyl)acetamide***2d**. Crude product was purified by column chromatography, eluent methyl-tert-butyl ether (R_f_ = 0.6). White solid; yield 2.1 g (86%); m.p. 68–69 °C. IR (KBr): 3295, 3103, 2999, 2765, 2387, 2310, 2116, 1685, 1586, 1512, 1474, 1431, 1377, 1304, 1266, 1180, 1136, 1074, 1044, 1007, 889, 853, 776, 670, 603, 576, 549, 522, 476, 455, 431 cm^−1^. ^1^H NMR (CDCl_3_, 500 MHz): δ = 2.17 (s, 3H, CH_3_), 7.20 (t, *^3^J_H-H_* = 7.5 Hz, 1H, Ar-H), 7.39 (d, *^3^J_H-H_* = 7.5 Hz, 1H, Ar-H), 7.61 (t, *^3^J_H-H_* = 7.5 Hz, 1H, Ar-H), 8.55 (d, *^3^J_H-H_* = 7.5 Hz, 1H, Ar-H), 10.00 (s, 1H, NH). ^19^F NMR (CDCl_3_, 376.5 MHz): δ = -72.3 (s). ^13^C NMR (CDCl_3_, 125.7 MHz): δ = 24.9, 118.1, 123.0, 123.6, 125.4 (q, *^1^J_C-F_* = 337 Hz, CF_3_), 128.5, 135.1, 142.2, 168.9. Anal. Calcd for C_9_H_8_F_3_NO_2_S: C, 43.03; H, 3.21; N, 5.58. Found: C, 43.00; H, 3.18; N, 5.57.

*1-Fluoro-4-((trifluoromethyl)sulfinyl)benzene***2e** [9]. Crude product contains 97 mol% of **2e** and 3 mol% of starting sulfide, which may be removed in vacuum (1 mBar) to give pure sulfoxide **2e**. Light yellow liquid; yield 1.9 g (96%). ^1^H NMR (CDCl_3_, 400 MHz): δ = 7.25–7.29 (m, 2H, Ar-H), 7.76–7.80 (m, 2H, Ar-H). ^19^F NMR (DMSO-*d_6_*, 376.5 MHz): δ = −75.7 (s, 3F, CF_3_), −104.8 (s, 1F, Ar-F). ^13^C NMR (CDCl_3_, 100.6 MHz): δ = 117.2 (d, *^2^J_C-F_* = 22 Hz), 124.6 (q, *^1^J_C-F_* = 335 Hz, CF_3_), 128.5 (d, *^3^J_C-F_* = 9 Hz), 131.1, 166.0, (d, *^1^J_C-F_* = 254.5 Hz, C-F). Compound **1e** was oxidized on a 50 g scale (in 70 mL of CF_3_COOH) with the same yield.

*1-Fluoro-2-((trifluoromethyl)sulfinyl)benzene***2f**. White solid; yield 2.0 g (98%); m.p. 44–45 °C. IR (KBr): 3094, 2124, 1986, 1948, 1820, 1724, 1633, 1598, 1474, 1449, 1261, 1141, 955, 867, 823, 762, 706, 673, 579, 544, 490, 459, 430 cm^−1^. ^1^H NMR (CDCl_3_, 400 MHz): δ = 7.19–7.23 (m, 1H, Ar-H), 7.42–7.46 (m, 1H, Ar-H), 7.61–7.67 (m, 1H, Ar-H), 7.94–7.98 (m, 1H, Ar-H). ^19^F NMR (CDCl_3_, 376.5 MHz): δ = −75.9 (s, 3F, CF_3_), −114.5 (s, 1F, Ar-F). ^13^C NMR (CDCl_3_, 125.7 MHz): δ = 116.4 (d, *^2^J_C-F_* = 20 Hz), 123.5 (dq, *^3^J_C-F_* = 15 Hz, *^4^J_C-F_* 2.5 Hz), 124.9 (qd, *^1^J_C-F_* = 335.5 Hz, *^4^J_C-F_* = 3.8 Hz, CF_3_),125.6 (d, *^4^J_C-F_* = 3.8 Hz), 127.2, 135.5 (d, *^3^J_C-F_* = 7.5 Hz), 159.7 (d, *^1^J_C-F_* = 252.7 Hz, C-F). Anal. Calcd for C_7_H_4_F_4_OS: C, 39.63; H, 1.90; S, 15.11. Found: C, 39.62; H, 1.91; S, 15.11.

*1-Bromo-4-((trifluoromethyl)sulfinyl)benzene***2g** [30,38]. Crude product contains 97 mol% of **2g** and 3 mol% of starting sulfide, which may be removed by washing with pentane to give pure product **2g**. White solid; yield 2.6 g (96%); m.p. 57–58 °C (Lit. 56–57 °C). ^1^H NMR (CDCl_3_, 500 MHz): δ = 7.66 (d, *^3^J_H-H_* = 8.5 Hz, 2H, Ar-H), 7.75 (d, *^3^J_H-H_* = 8.5 Hz, 2H, Ar-H). ^19^F NMR (CDCl_3_, 470 MHz): δ = −74.4 (s). ^13^C NMR (CDCl_3_, 125.7 MHz): δ = 124.4 (q, *^1^J_C-F_* = 335.6 Hz, CF_3_), 127.3, 128.6, 133.0, 134.7. Compound **1g** was oxidized on a 15 g scale (in 35 mL of CF_3_COOH) with the same yield.

*2-((Trifluoromethyl)sulfinyl)-acetophenone***2h**. Crude product contains 97 mol% of **2h** and 3 mol% of starting sulfide, which may be removed by washing with pentane to give pure product **2h**. Pale yellow solid; yield 2 g (85 %); m.p. 97–98 °C. IR (KBr): 3080, 2968, 1675, 1585, 1468, 1438, 1359, 1280, 1187, 1130, 1073, 961, 885, 772, 663, 602, 571, 483, 434 cm^−1^. ^1^H NMR (CDCl_3_, 400 MHz): δ = 2.68 (s, 3H, CH_3_), 7.74 (t, *^3^J_H-H_*= 7 Hz, 1H, Ar-H), 7.88 (t, *J* = 7 Hz, 1H, Ar-H), 8.04 (d, *^3^J_H-H_* = 7 Hz, 1H, Ar-H), 8.36 (d, *^3^J_H-H_*= 7 Hz, 1H, Ar-H). ^19^F NMR (CDCl_3_, 376.5 MHz): δ = −70.0 (s). ^13^C NMR (CDCl_3_, 100.6 MHz): δ = 26.2, 125.1 (q, *^1^J_C-F_* = 340 Hz, CF_3_), 126.0, 131.0, 132.4, 134.2, 135.4, 140.3, 198.3. Anal. Calcd for C_9_H_7_F_3_O_2_S: C, 45.76; H, 2.99; S, 13.57. Found: C, 45.78; H, 3.01; S, 13.60.

*2-((Trifluoromethyl)sulfinyl)phenyl acetate***2i**. Crude product was purified by column chromatography, eluent hexane-methyl-tert-butyl ether, 2:1 (R_f_ = 0.5), but the product contains ~15% of the corresponding phenol. Pale yellow oil; yield 1.8 g (70 %). ^1^H NMR (CDCl_3_, 500 MHz): δ = 2.33 (s, 3H, CH_3_), 7.05 (d, *^3^J_H-H_* = 8 Hz, 1H, Ar-H), 7.53 (t, *^3^J_H-H_* = 8 Hz, 1H, Ar-H), 7.68 (t, *^3^J_H-H_* = 8 Hz, 1H, Ar-H), 8.03 (d, *^3^J_H-H_* = 8 Hz, 1H, Ar-H). ^19^F NMR (CDCl_3_, 470 MHz): δ = −73.5 (s). ^13^C NMR (CDCl_3_, 125.7 MHz): δ = 20.5, 123.4, 124.9 (q, *^1^J_C-F_* = 336 Hz, CF_3_), 126.8, 127.0, 127.8, 134.5, 148.6, 167.8.

*2,4-Dinitro-1-((trifluoromethyl)sulfinyl)benzene***2j**. Crude product was purified by column chromatography, eluent hexane-methyl-*tert*-butyl ether, 5:1 (R_f_ = 0.2). Pale yellow solid; yield 2.4 g (83 %), m.p. 85–87 °C. IR (KBr): 3110, 3039, 2883, 1611, 1544, 1347, 1224, 1186, 1134, 1074, 917, 896, 860, 835, 742, 678, 602, 574, 531, 496, 439 cm^−1^. ^1^H NMR (DMSO-*d_6_*, 500 MHz): δ = 8.46 (d, *^3^J_H-H_* = 8 Hz, 1H, Ar-H), 8.92 (d,*^3^J_H-H_* = 8 Hz, 1H, Ar-H), 8.97 (s, 1H, Ar-H). ^19^F NMR (CDCl_3_, 376.5 MHz): δ = −68.5 (s). ^13^C NMR (DMSO-d_6_, 125.7 MHz): δ = 121.0, 125.7 (q, *^1^J_C-F_* = 342 Hz, CF_3_), 128.8, 130.4, 142.1, 146.9, 150.8. Anal. Calcd for C_7_H_3_F_3_N_2_O_5_S: C, 29.59; H, 1.06; N, 9.86; S, 11.28. Found: C, 29.58; H, 1.05; N, 9.87; S, 11.29.

*2-((Trifluoromethyl)sulfinyl)naphthalene***2k**. Product was purified by column chromatography, eluent Et_2_O-petroleum ether, 1:9 (R_f_ = 0.6). Pale yellow liquid; yield 1.4 g (87%). IR (KBr): 2940, 1171, 1126, 1073, 813, 744,639 cm^−1^. ^1^H NMR (300 MHz, CDCl_3_): δ = 7.53–7.67 (m, 2H, Ar-H), 7.75 (d, *^3^J_H-H_* = 8.6 Hz, 1H, Ar-H), 7.85–7.96 (m, 2H, Ar-H), 7.99 (d, *^3^J_H-H_* = 8.7 Hz, 1H, Ar-H), 8.31 (s, 1H, Ar-H). ^19^F NMR (188 MHz, CDCl_3_): δ = −74.5 (s). ^13^C NMR (75 MHz, CDCl_3_): δ = 120.4, 124.9 (q, *^1^J_C-F_* = 335 Hz, CF_3_), 127.7, 127.8, 128.2, 128.8, 129.0, 129.9, 132.4, 132.5, 132.6, 135.5. HRMS: calcd. for C_11_H_7_F_3_SONa 267.0063; found 267.0067 [M − Na]+ (δ = −1.5).

*1-Chloro-2-((trifluoromethyl)sulfinyl)ethane***4a** [14]. Product was purified by distillation. Colorless liquid; yield 1.5 g (83%); b.p. 68–70 °C (10 Torr). ^1^H NMR (CDCl_3_, 400 MHz): δ = 3.21–3.24 (m, 1H, CH_2_), 3.43–3.48 (m, 1H, CH_2_), 3.95–4.00 (m, 2H, CH_2_). ^19^F NMR (CDCl_3_, 376.5 MHz): δ = −73.6 (s). Compound **3a** was oxidized on a 15 g scale (in 35 mL of CF_3_COOH) with the same yield.

*2-(Trifluoromethyl)sulfinyl-ethanol***4b**. Crude product was purified by distillation, but the product contains 11% of starting sulfide. Colorless liquid; yield 0.25 g (15%); b.p. 108–110 °C (10 Torr). IR (KBr): 3417, 2893, 1396, 1293, 1174, 1138, 1047, 1004, 946, 844, 749, 645, 563, 442 cm^−1^. ^1^H NMR (CDCl_3_, 500 MHz): δ = 3.02–3.20 (m, 2H, CH_2_), 3.67 (s, 1H, OH), 4.09–4.13 (m, 2H, CH_2_). ^19^F NMR (CDCl_3_, 470 MHz): δ = −73.6 (s). ^13^C NMR (CDCl_3_, 125.7 MHz): δ = 51.2, 54.4, 125.5 (q, *^1^J_C-F_* = 332 Hz, CF_3_).

*2-((Trifluoromethyl)sulfinyl)ethyl acetate***4c**. Crude product was purified by distillation, but the product contains 15% of the corresponding alcohol. Colorless liquid; yield 1.1 g (54%); b.p. 100–102 °C (10 Torr). ^1^H NMR (CDCl_3_, 500 MHz): δ = 2.10 (s, 3H, CH_3_), 3.20–3.33 (m, 2H, CH_2_), 4.47–4.64 (m, 2H, CH_2_). ^19^F NMR (CDCl_3_, 470 MHz): δ = −73.7 (s). ^13^C NMR (CDCl_3_, 125.7 MHz): δ = 20.4, 48.2, 55.9, 125.3 (q, *^1^J_C-F_* = 333 Hz, CF_3_), 170.3.

*Methyl 2-((trifluoromethyl)sulfinyl)acetate***4d** [16]. Product was purified by distillation. Colorless liquid; yield 1.1 g (60%); b.p. 90 °C (10 Torr). ^1^H NMR (CDCl_3_, 500 MHz): δ = 3.79 (s, 3H, CH_3_), 3.87 (d, *^2^J_H-H_* = 15 Hz, 1H, CH_2_), 3.94 (d, *^2^J_H-H_* = 15 Hz, 1H, CH_2_). ^19^F NMR (CDCl_3_, 376.5 MHz): δ = −73.8 (s). ^13^C NMR (CDCl_3_, 125.7 MHz): δ = 53.5, 53.6, 125.1 (q, *^1^J_C-F_* = 334.5 Hz, CF_3_), 164.2.

*Methyl 3-((trifluoromethyl)sulfinyl)propanoate***4e**. Product was purified by distillation. Colorless liquid; yield 1.25 g (60%); b.p. 96–98 °C (10 Torr). IR (KBr): 2959, 1735, 1440, 1367, 1173, 1136, 1074, 980, 828, 746, 661, 564, 450 cm^−1^. ^1^H NMR (CDCl_3_, 400 MHz): δ = 2.77–2.97 (m, 2H, CH_2_), 3.17–3.27 (m, 2H, CH_2_), 3.72 (s, 3H, CH_3_). ^19^F NMR (CDCl_3_, 376.5 MHz): δ = −74.2 (s). ^13^C NMR (CDCl_3_, 125.7 MHz): δ = 25.6, 43.2 (q, *^3^J_C-F_* = 2.5 Hz, CH_2_-S(O)CF_3_), 52.4, 125.4 (q, *^1^J_C-F_* = 334.5 Hz, CF_3_), 170.8. Anal. Calcd for C_5_H_7_F_3_O_3_S: C, 29,42; H, 3,46; S, 15.70. Found: C, 29.43; H, 3.44; S, 15.72.

*7-((Trifluoromethyl)sulfinyl)heptanenitrile***4f**. Compound **3f** was oxidized on a 0.12 g scale. Product was purified by preparative TLC chromatography, eluent Et_2_O-petroleum ether, 1:9 (R_f_ = 0.1). Pale yellow liquid; yield 0.11 g (85%). IR (KBr): 2936, 2864, 2239, 1171, 1143, 1080 cm^−1^. ^1^H NMR (CDCl_3_, 300 MHz): δ = 1.40–1.56 (m, 4H, CH_2_ -CH_2_), 1.56–1.76 (m, 2H, CH_2_), 1.74–1.92 (m, 2H, CH_2_), 2.31 (t, *^3^J_H-H_* = 6.9 Hz, 2H, CH_2_), 2.73–2.90 (m, 1H, CH), 2.93–3.11 (m, 1H, CH). ^19^F NMR (CDCl_3_, 188 MHz): δ = −74.1 (s). ^13^C NMR (CDCl_3_, 75 MHz): δ = 17.0, 21.5, 24.9, 27.8, 28.1, 48.2, 119.5, 125.3 (q, *^1^J_C-F_* = 333 Hz, CF_3_). HRMS: calcd. for C_8_H_12_NF_3_SONa 250.0477; found 250.0489 [M − Na]+ (δ = 0.4).

*1-Fluoro-4-(((trifluoromethyl)sulfinyl)methyl)benzene***4g** [39]. Compound **3g** was oxidized on a 0.05 g scale. Product was purified by preparative TLC chromatography, eluent Et_2_O-petroleum ether, 1:9 (R_f_ = 0.3). Pale yellow solid; yield 0.041 g (85%); m.p. 80 °C (Lit. 81–82 °C). IR (KBr): 2983, 2927, 1600, 1507, 1243, 1178, 1139, 1050, 1011, 842, 745 cm^−1^. ^1^H NMR (CDCl_3_, 300 MHz): δ = 4.20 (AB syst., *^2^J_H-H_* = 13.2 Hz, 2H, CH_2_), 7.11 (t, *^3^J_H-H_* = *^3^J_H-F_*= 8.4 Hz, 2H, Ar-H), 7.35 (dd, *^3^J_H-H_* = 8.4 Hz, *^4^J_H-F_* = 5.3 Hz, 2H, Ar-H). ^19^F NMR (CDCl_3_, 188 MHz): δ = −73.11 (s, 3F, CF_3_), −112.25(m, 1F, Ar-F). ^13^C NMR (CDCl_3_, 75 MHz): δ = 54.7 (q, *^3^J_C-F_* = 3.1 Hz), 116.5 (d, *^2^J_C-F_* = 21.9 Hz), 123.3 (d, *^4^J_C-F_* = 3.3 Hz), 125.3 (q, *^1^J_C-F_* = 335.0 Hz, CF_3_), 132.3 (d, *^3^J_C-F_* = 8.5 Hz), 163.4 (d, *^1^J_C-F_* = 249.3 Hz, C-F).

*((Difluoromethyl)sulfinyl)benzene***6a** [40]. Colorless liquid; yield 1.2 g (70%); b.p. 130 °C (10 Torr). ^1^H NMR (CDCl_3_, 400 MHz): δ = 6.02 (t, *^2^J_H-F_* = 55.6 Hz, 1H, CHF_2_), 7.55-7.60 (m, 3H, Ar-H), 7.69–7.71 (m, 2H, Ar-H). ^19^F {H} NMR (CDCl_3_, 376.5 MHz): δ = −119.4 (d, ^2^*J_F-F_* = 260 Hz, 1F, CHF_2_), -120.2 (d, ^2^*J_F-F_* = 260 Hz, 1F, CHF_2_). ^13^C NMR (CDCl_3_, 100.6 MHz): δ = 120.8 (dd, *^1^J_C-F_*= 288 Hz, 287 Hz, CHF_2_), 125.4, 129.5, 132.8, 136.6 (t, *^3^J_C-F_* = 4 Hz).

*N-(4-((Difluoromethyl)sulfinyl)phenyl)acetamide***6b** [30]. Pale yellow solid; yield 1.9 g (80%); m.p. 166–167 °C (Lit. 170–171 °C). ^1^H NMR (DMSO-*d_6_*, 500 MHz): δ = 2.09 (s, 3H, CH_3_), 6.89 (t, *^2^J_H-F_* = 54 Hz, 1H, CHF_2_), 7.70 (d, *^3^J_H-H_* = 8 Hz, 2H, Ar-H), 7.85 (d, *^3^J_H-H_* = 8 Hz, 2H, Ar-H), 10.34 (s, 1H, NH). ^19^F NMR (DMSO-d_6_, 376.5 MHz): δ = -121.4 (dd, ^2^*J_F-F_* = 257 Hz, ^2^*J_H-F_* = 54 Hz, 1F, CHF_2_), -125.5 (dd, ^2^*J_F-F_* = 257 Hz, ^2^*J_H-F_* = 54 Hz, 1F, CHF_2_). ^19^F {H} NMR (CDCl_3_, 376.5 MHz): δ = −121.5 (d, ^2^*J_F-F_* = 257 Hz, 1F, CHF_2_), -123.3 (d, ^2^*J_F-F_* = 257 Hz, 1F, CHF_2_). ^13^C NMR (DMSO-*d_6_*, 125.7 MHz): δ = 24.6, 119.8, 120.6 (t, *^1^J_C-F_* = 292 Hz, CHF_2_), 120.9 (t, *^1^J_C-F_* = 280 Hz, CHF_2_), 127.2, 127.3, 129.9, 143.8, 169.5.

*1-((Difluoromethyl)sulfinyl)-4-nitrobenzene***6c** [30]. Pale yellow solid; yield 1.7 g (75%); m.p. 84–85 °C (Lit. 81–83 °C). ^1^H NMR (DMSO-*d_6_*, 500 MHz): δ = 7.08 (t, *^2^J_H-F_* = 56 Hz, 1H, CHF_2_), 8.04 (d, *^3^J_H-H_* = 6.5 Hz, 2H, Ar-H), 8.46 (d, *^3^J_H-H_* = 6.5 Hz, 2H, Ar-H). ^19^F NMR (DMSO-*d_6_*, 376.5 MHz): δ = −120.8 (dd, ^2^*J_F-F_* = 254 Hz, ^2^*J_H-F_* = 56 Hz, 1F, CHF_2_), -125.9 (dd, ^2^*J_F-F_* = 254 Hz, ^2^*J_H-F_* = 56 Hz, 1F, CHF_2_). ^19^F {H} NMR (DMSO-*d_6_*, 376.5 MHz): δ = −120.8 (d, ^2^*J_F-F_* = 254 Hz, 1F, CHF_2_), −125.9 (d, ^2^*J_F-F_* = 254 Hz, 1F, CHF_2_). ^13^C NMR (DMSO-*d_6_*, 125.7 MHz): δ = 120.4 (t, *^1^J_C-F_* = 284 Hz, CHF_2_), 120.6 (t, *^1^J_C-F_* = 287 Hz, CHF_2_), 124.7, 124.9, 127.4, 127.5, 133.9, 144.3, 150.4.

*(1,1,2,2-Tetrafluoro-2-((1,2,4-triazol)-1-yl)-ethyl)-sulfinylbenzene***6d**. Crude product was purified by column chromatography, eluent CH_2_Cl_2_, then methyl-tert-butyl ether (R_f_ = 0.7). Pale yellow solid; yield 2.7 g (92%); m.p. 77–78 °C. IR (KBr): 3105, 2872, 1819, 1688, 1582, 1512, 1477, 1453, 1403, 1378, 1294, 1262, 1221, 1191, 1140, 1111, 1070, 981, 903, 830, 756, 691, 667, 629, 591, 514, 482, 449 cm^−1^. ^1^H NMR (CDCl_3_, 500 MHz): δ = 7.56–7.65 (m, 3H, Ar-H), 7.77 (d, 2H, Ar-H), 8.15 (s, 1H, Het-H), 8.59 (s, 1H, Het-H). ^19^F NMR (CDCl_3_, 376.5 MHz): δ = −94.0 (d, *^2^J_F-F_* = 226 Hz, 1F, CF_2_), −94.6 (d, *^2^J_F-F_* = 226 Hz, 1F, CF_2_), −112.5 (d, *^2^J_F-F_* = 237 Hz, 1F, CF_2_), 124.3 (d, *^2^J_F-F_* = 237 Hz, 1F, CF_2_). ^13^C NMR (CDCl_3_, 125.7 MHz): δ = 113.0 (tt, *^1^J_C-F_* = 271 Hz, *^2^J_C-F_* = 31 Hz, CF_2_), 116.9 (ddt, *^1^J_C-F_* = 318 Hz, *^1^J_C-F_* = 308 Hz, *^2^J_C-F_* 38 Hz, CF_2_), 126.7, 129.5, 133.8, 135.0, 143.4, 154.1. Anal. Calcd for C_12_H_12_F_4_N_3_OS: C, 44.72; H, 3.75; N, 13.04; S, 9.95. Found: C, 44.74; H, 3.76; N, 13.02; S, 9.98.

*((Bromodifluoromethyl)sulfinyl)benzene***6e** [41]. Compound **5e** was oxidized only on a 50 mmol scale. Crude product was purified by column chromatography, eluent petroleum ether-diethyl ether, 8:2 (R_f_ = 0.6). Yellow oil; yield 8.1 g (91%). IR (KBr):1443, 1114, 1073, 1056, 854, 827, 742, 687 cm^−1^. ^1^H NMR (CDCl_3_, 200 MHz): δ= 7.52 (m, 3H, ArH), 7.72 (m, 2H, ArH). ^19^F NMR (CDCl_3_, 188 MHz): δ = -53.49 and -55.56 (2F, CF_2_Br, syst. AB *^2^J_C-F_* = 145 Hz). ^13^C NMR (CDCl_3_, 50 MHz): δ = 126.2, 128.4 (dd, *^1^J_C-F_* = 355 Hz, 350.5Hz), 129.1, 133.4, 136.7 (dd, *^3^J_C-F_* = 3 Hz, 1 Hz).

*((Dichlorofluoromethyl)sulfinyl)benzene***6f** [42]. Compound **5f** was oxidized only on a 50 mmol scale. Crude product was purified by column chromatography, eluent petroleum ether-diethyl ether, 8:2 (R_f_ = 0.5). Yellow oil; yield 10.6 g (69%). IR (KBr): 1445, 1104, 1061, 840, 792, 744, 684 cm^−1^. ^1^H NMR (CDCl_3_, 200 MHz): δ = 7.61 (m, 3H, Ar-H), 7.82 (m, 2H, Ar-H). ^19^F NMR (CDCl_3_, 188 MHz): δ = −62.8 (s). ^13^C NMR (CDCl_3_, 50 MHz): δ = 126.9 (d, *^1^J_C-F_* = 340 Hz), 126.9 (d, *^4^J_C-F_* = 1.5 Hz), 128.9, 133.5, 137.4 (d, *^3^J_C-F_* = 1.5 Hz).

*N-(6-((Trifluoromethyl)sulfinyl)pyridin-3-yl)acetamide***8**. Crude product was purified by column chromatography, eluent ethyl acetate (R_f_ = 0.5) to give compound **8** with 80 % purity (contains ~20% of starting sulfide). Yellow solid; yield 0.75 g (~30%). ^1^H NMR (DMSO-*d_6_*, 400 MHz): δ = 2.12 (s, 3H, CH_3_), 8.02 (d, *^3^J_H-H_* = 8.8 Hz, 1H, Het-H), 8.41 (d, *^3^J_H-H_* = 8.8 Hz, 1H, Het-H), 10.6 (s, 1H, NH), 8.88 (s, 1H, Het-H). ^19^F {H} NMR (DMSO-*d_6_*, 376.5 MHz): δ = −72.7 (s). ^13^C NMR (CDCl_3_, 125.7 MHz): δ = 24.4, 122.5, 125.3 (q, *^1^J_C-F_* = 338 Hz, CF_3_), 127.9, 139.8, 141.3, 149.3, 170.1.

## 4. Conclusions

In summary, a simple, convenient, and high-yielding procedure for the oxidation of fluoroalkyl sulfides to fluoroalkyl sulfoxides was developed. It was shown that the new protocol is suitable for a wide range of aliphatic and aromatic substrates on a milligram and multigram scales. In almost all cases, over-oxidation did not occur, and the crude products contained only a small quantity of starting sulfides.

## Figures and Tables

**Table 1:** Oxidation of aryl trifluoromethyl sulfides **1**.

Entry	Reagent	Product	Molar ratio in Crude Product ^1^			Crude^2^ Yield, %	Isolated Yield, %
			1	2	ArSO_2_CF_3_		
1	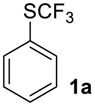	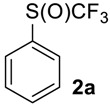	5	95	0	95	-
2	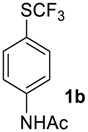	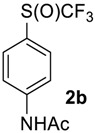	7	93	0	91	85
3	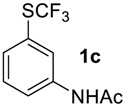	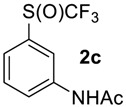	4	96	0	96	-
4	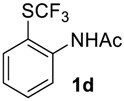	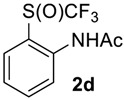	2	98	traces	89	86
5	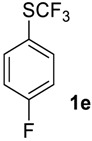	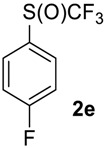	3	97	0	100	96
6	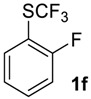	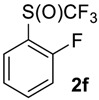	0	100	0	98	-
7	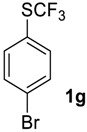	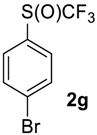	3	97	0	100	96
8	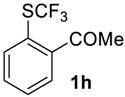	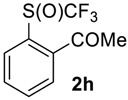	3	97	0	90	85
9	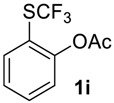	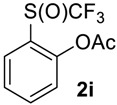	3	97 ^3^	0	84	70 ^3^
10	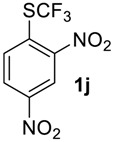	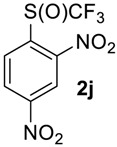	7	91	2	100	83
11	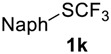	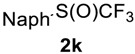	0	100	0	-	87

^1^ Composition of crude product on the basis of ^19^F NMR data; ^2^ Crude yield is the isolated yield of product after removing the solvent before further purification; ^3^ Mixture of **2i** and corresponding phenol in 85:15 molar ratio (according to ^19^F NMR data).

**Table 2:** Oxidation of alkyl trifluoromethyl sulfides **3**.

Entry	Reagent	Product	Molar ratio in Crude Product ^1^			Crude^2^ Yield, %	Isolated Yield, %
			3	4	AlkSO_2_CF_3_		
1	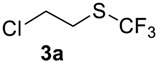	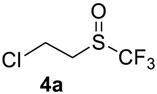	4	96	0	-	83
2	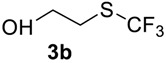	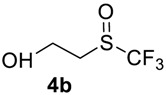	11	89	traces	-	15 ^3^
3	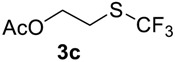	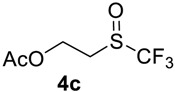	0	97 ^4^	3	-	54 ^4^
4	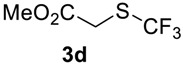	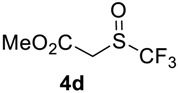	10	90	0	-	60 ^5^
5	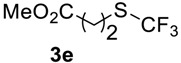	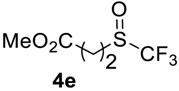	4	96	0	63	60
6	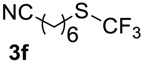	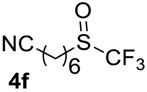	13	87	traces	-	85
7	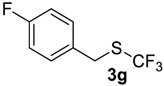	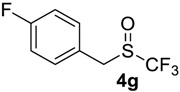	0	99	1	-	85

^1^ Composition of crude product on the basis of ^19^F NMR data; ^2^ Crude yield is the isolated yield of product after removing the solvent before further purification; ^3^ Contains ~11 % of starting sulfide **3b**; ^4^ Mixture of **4c** and corresponding alcohol **4b** in 85:15 molar ratio (according to ^19^F NMR data); ^5^ Use of 10% excess of H_2_O_2_ lead to the same results.

**Table 3:** Oxidation of fluoromethyl and difluoromethyl sulfides **5**.

Entry	Reagent	Product	Molar ratio in Crude Product ^1^			Crude^2^Yield, %	Isolated Yield, %
			5	6	ArSO_2_R_F_		
1	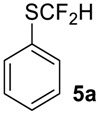	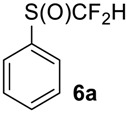	0	100	0	-	70 ^3^
2	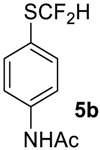	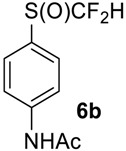	4	96	0	80	-
3	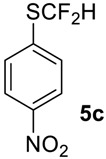	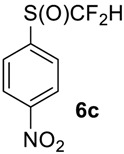	4	96	0	75	-
4	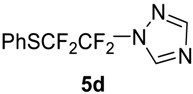	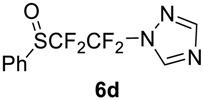	5	95	0	95	92
5	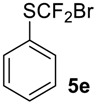	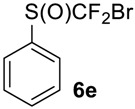	3	96	1	-	91
6	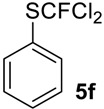	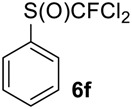	0	100	0	-	69

^1^ Composition of crude product on the basis of ^19^F NMR data; ^2^ Crude yield is the isolated yield of product after removing the solvent before further purification; ^3^ Reaction time 4 h.

## Supplementary Materials

The following are available online: titration of H_2_O_2_ solution, NMRs and IRs of the new synthesized compounds.
