# Supplementary material for: General, Practical and Selective Oxidation Protocol for CF3S into CF3S(O) Group
Source: Molecules. 2019 Mar 30;24(7):1249. doi: 10.3390/molecules24071249 (PMC6479840; doi:10.3390/molecules24071249)

SUPPLEMENTARY MATERIALS

General, Practical and Selective Oxidation Protocol for CF_3S into $CF_3S(O)$ group

Liubov V. Sokolenko[1], Raisa K. Orlova[1], Andrey A. Filatov[1], Yurii L. Yagupolskii[1,3,*], Emmanuel Magnier[2,*], Bruce Pégot[2], Patrick Diter[2]

[1] Institute of Organic Chemistry, National Academy of Sciences of Ukraine, Murmans'ka Str. 5, 02094 Kyiv, Ukraine; yagupolskii@ioch.kiev.ua

[2] Bâtiment Lavoisier, Université de Versailles-Saint-Quentin, 45 avenue des Etats-Unis, 78035 Versailles, France; emmanuel.magnier@uvsq.fr

[3] Enamine Ltd, A. Matrosova str. 23, Kiev 01103, Ukraine

* Correspondence: yagupolskii@ioch.kiev.ua (Y.Y.); emmanuel.magnier@uvsq.fr (E.M.)

Content

Measurement of H_2O_2 solution concentration …………………………………….. 2

Spectra of new products……………………………………………………………….. 3

N-(2-((Trifluoromethyl)sulfinyl)phenyl)acetamide **2d**………………………………… 3

1-Fluoro-2-((trifluoromethyl)sulfinyl)benzene **2f**……………………………………… 5

2-((Trifluoromethyl)sulfinyl)-acetophenone **2h**……………………………………….. 8

2-((Trifluoromethyl)sulfinyl)phenyl acetate **2i**……………………………………... 10

2,4-Dinitro-1-((trifluoromethyl)sulfinyl)benzene **2j**…………………………………… 13

2-((Trifluoromethyl)sulfinyl)naphthalene **2k**………………………………………….. 15

2-(Trifluoromethyl)sulfinyl-ethanol **4b**…………………………………………...… 17

2-((Trifluoromethyl)sulfinyl)ethyl acetate **4c**………………………………………….. 20

Methyl 3-((trifluoromethyl)sulfinyl)propanoate **4e**…………………………………… 21

7-((Trifluoromethyl)sulfinyl)heptanenitrile **4f**………………………………………… 23

(1,1,2,2-Tetrafluoro-2-((1,2,4-triazol)-1-yl)-ethyl)-sulfinylbenzene **6d**………………. 25

N-(6-((Trifluoromethyl)sulfinyl)pyridin-3-yl)acetamide **8**…………………………….. 28

Oxidation of 2-((trifluoromethyl)thio)-1,3,4-thiadiazole **9**…………..………..…….. 30

Measurement of the H_2O_2 solution concentration by densimetry

Measurements of density were performed using set of hydrometers (notably, hydrometer with measurement range 1.000 to 1.060 g/cm^3) "AOH-1" (Minpribor USSR, Production Association "Khimlaborpribor", 1991 model year).

Table. Densities of H_2O_2 solutions at 25ºC.

% H_2O_2 (w/w)	density g/cm^3	% H_2O_2 (w/w)	density g/cm^3
0·0	(0·9970)	49·88	1·1909
1·91	1·0036	53·48	1·2067
5·99	1·0179	55·08	1·2143
9·23	1·0295	59·68	1·2353
13·47	1·0452	62·55	1·2490
17·44	1·0597	64·88	1·2593
21·14	1·0737	68·97	1·2790
24·58	1·0868	73·15	1·2996
27·96	1·0996	73·66	1·3024
29·99	1·1078	76·66	1·3166
34·23	1·1244	80·64	1·3378
39·12	1·1450	84·97	1·3595
40·67	1·1515	89·68	1·3848
42·97	1·1610	89·78	1·3855
47·02	1·1785	89·89	1·3855

Reference:

Easton, M.F.; Mitchell, A.G.; Wynne-Jones, W.F.K. The behaviour of mixtures of hydrogen peroxide and water. Part 1.-Determination of the densities of mixtures of hydrogen peroxide and water. *Trans. Faraday. Soc.* **1952**, *48*, 796-801. DOI: 10.1039/TF9524800796

Measurement of the H_2O_2 solution concentration by titration. Hydrogen peroxide was quantitatively oxidized by titration with a potassium permanganate solution of known strength under acidic conditions.

The initial solution is diluted to a hundredth (1mL of the solution of hydrogen peroxide in a 100 mL volumetric flask). Then 10 mL of this diluted solution was mixed with 10 mL of sulfuric acid (at 20%) and 10 mL of water. This resulting mixture was tittered twice with a solution of potassium permanganate (c= 2.001 10-2 mol/L) until the first appearance of a faint pink color that persists for 30 seconds. An equivalent volume of 20.5 mL was obtained for both experiments giving a concentration of 10.255 mol/L or 348.67g/L

$$C_{H2O2} = 5/2\ (C_{KMnO4} * V_{eq}/V_{H2O2})$$

With the table of concentration in percent we deduced a concentration of 31.2% for the initial solution.

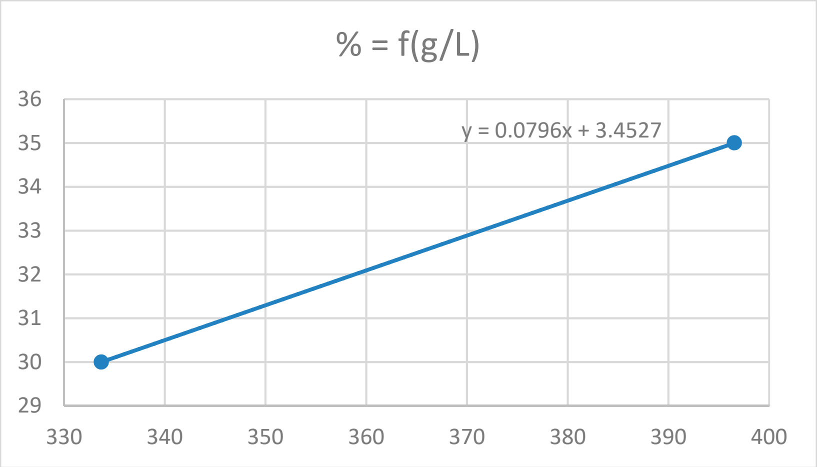

Spectra of new products

N-(2-((Trifluoromethyl)sulfinyl)phenyl)acetamide.

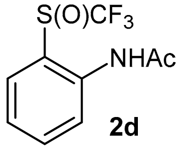

2d

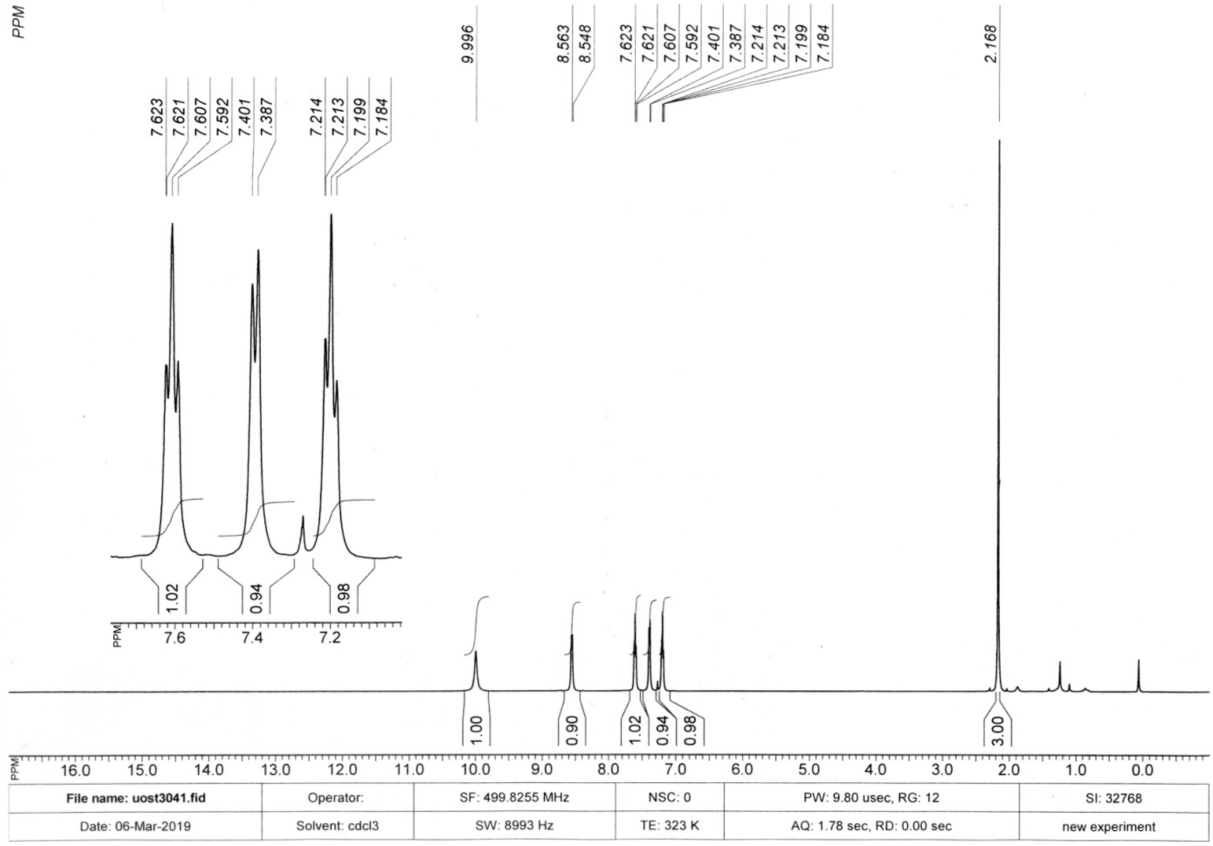

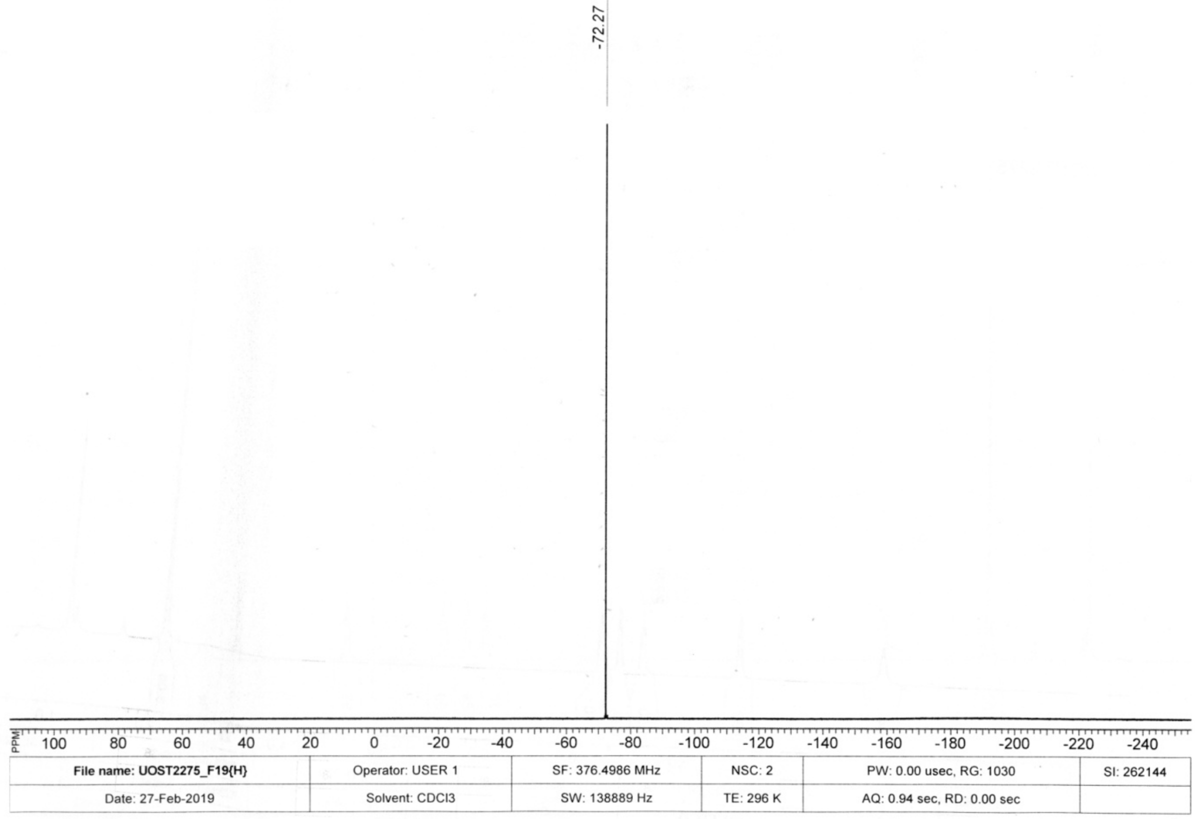

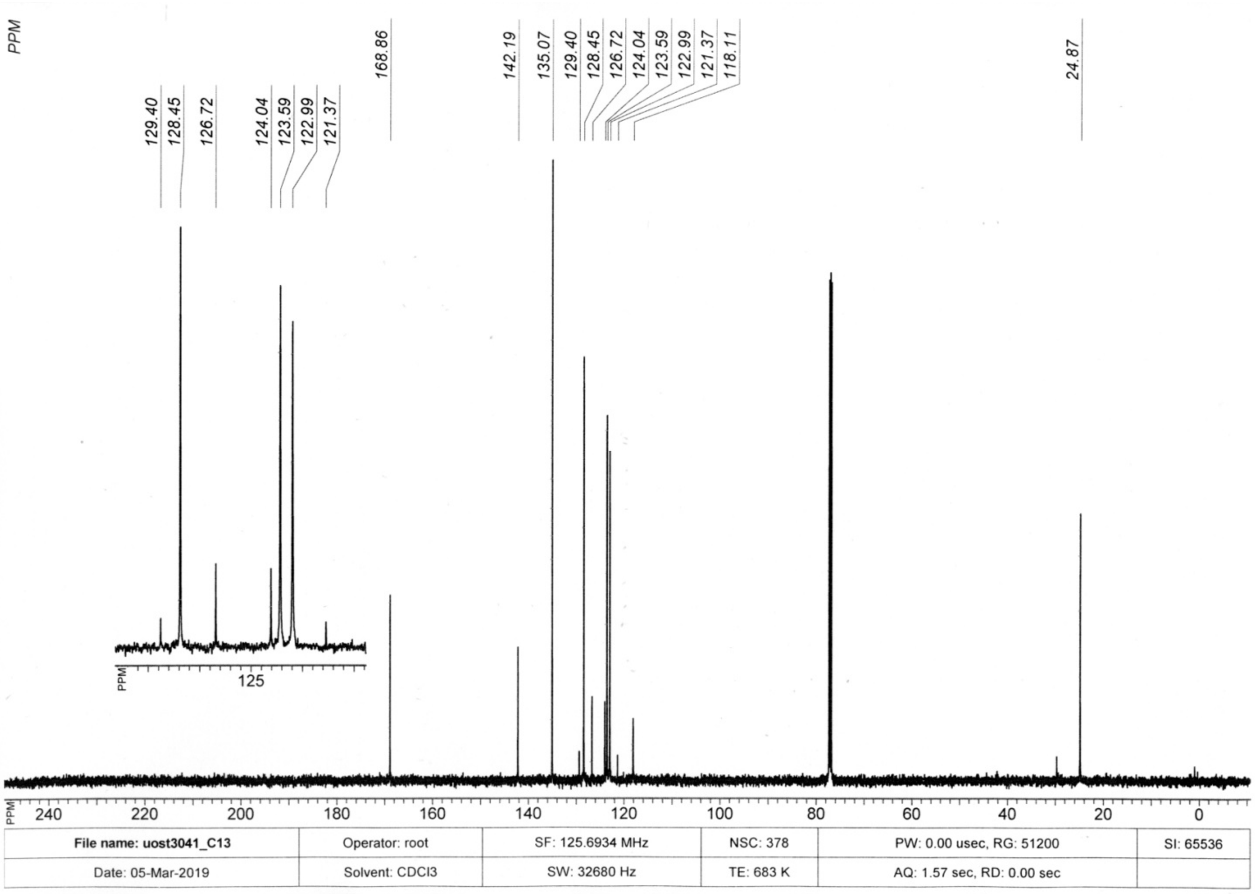
PPM
129.40
128.45
126.72
124.04
123.59
122.99
121.37
168.86
142.19
135.07
118.11
24.87
125
240 220 200 180 160 140 120 100 80 60 40 20 0
File name: uost3041_C13
Operator: root
SF: 125.6934 MHz
NSC: 378
PW: 0.00 usec, RG: 51200
SI: 65536
Date: 05-Mar-2019
Solvent: CDCl3
SW: 32680 Hz
TE: 683 K
AQ: 1.57 sec, RD: 0.00 sec

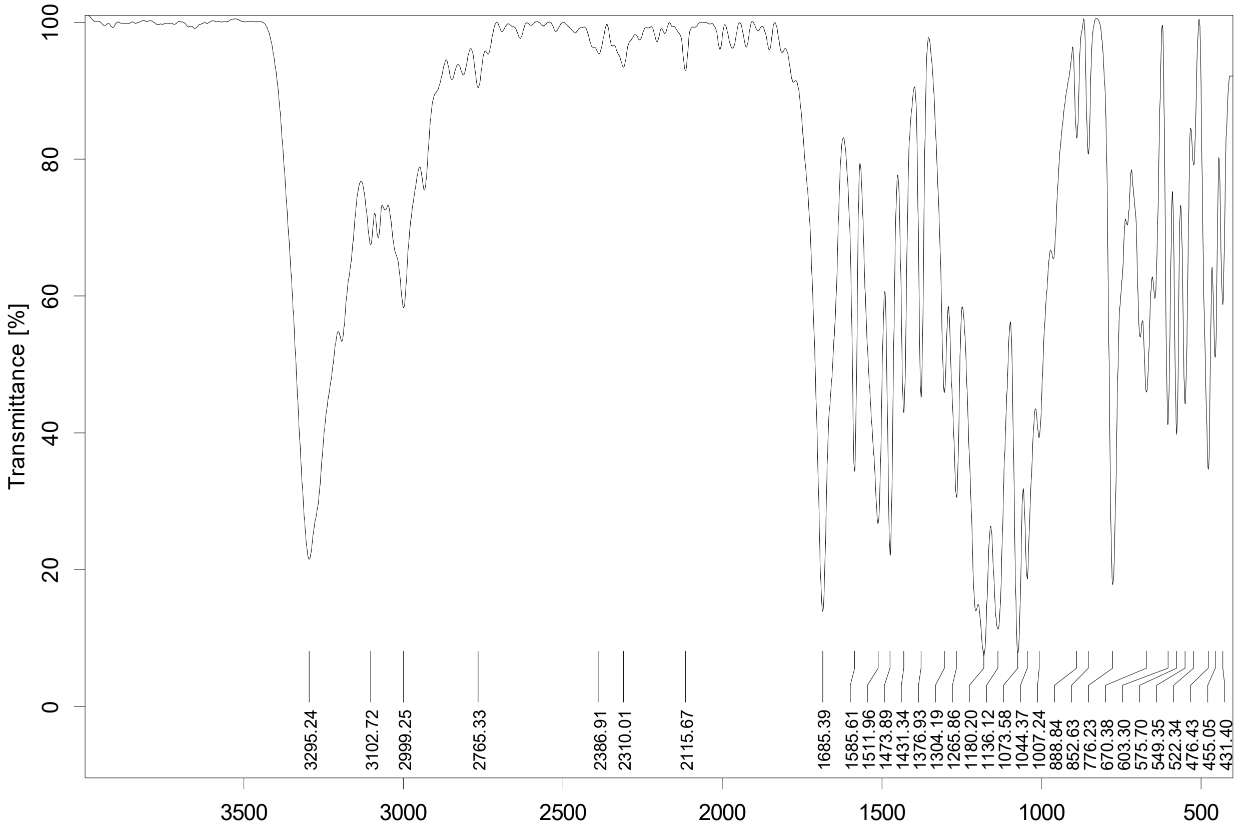
Transmittance [%]
Wavenumber cm-1
3295.24
3102.72
2999.25
2765.33
2386.91
2310.01
2115.67
1685.39
1585.61
1511.96
1473.89
1431.34
1376.93
1304.19
1265.86
1180.20
1136.12
1073.58
1044.37
1007.24
888.84
852.63
776.23
670.38
603.30
575.70
549.35
522.34
476.43
455.05
431.40

1-Fluoro-2-((trifluoromethyl)sulfinyl)benzene.

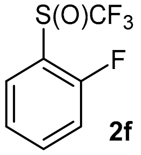

2f

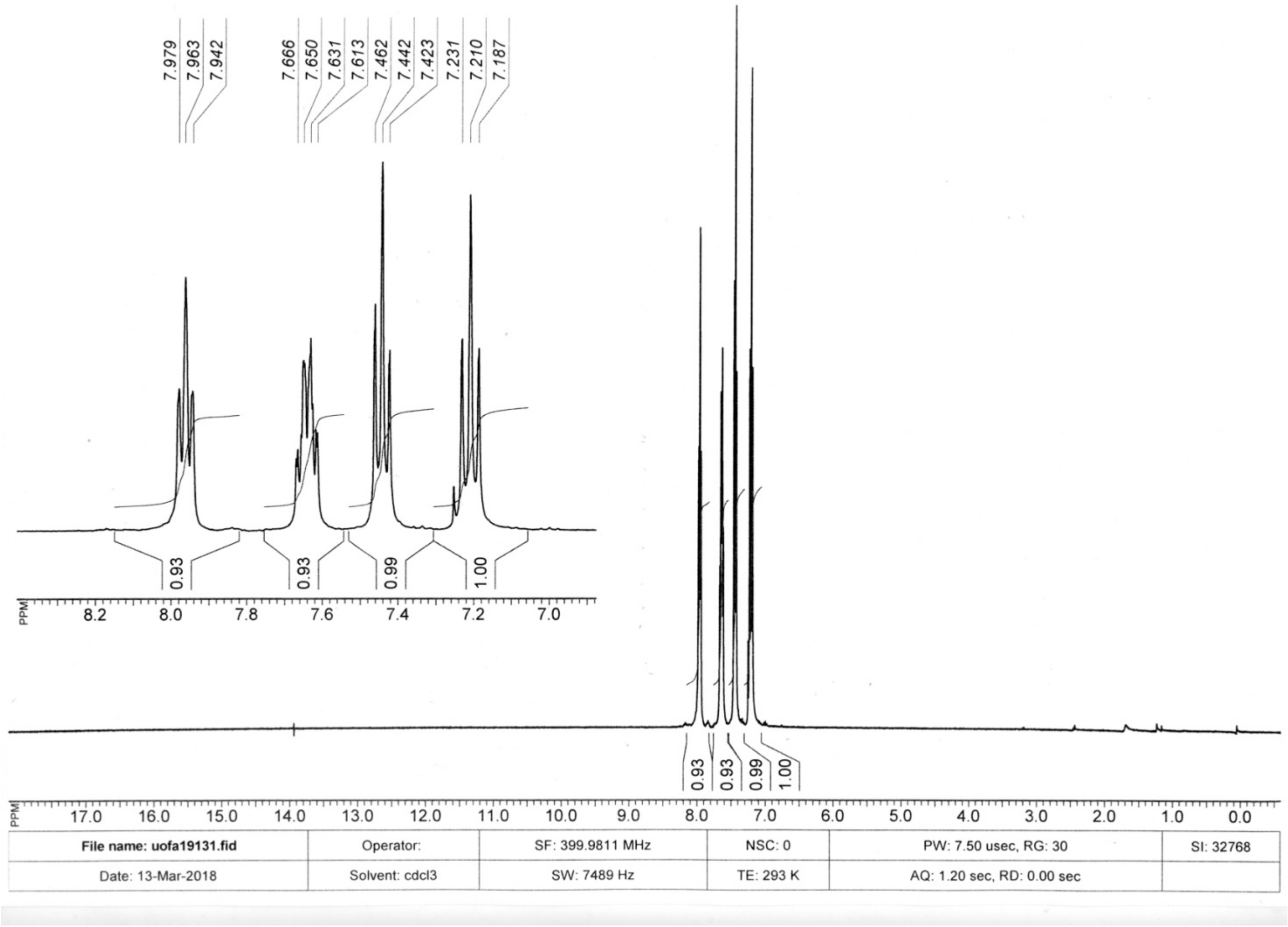

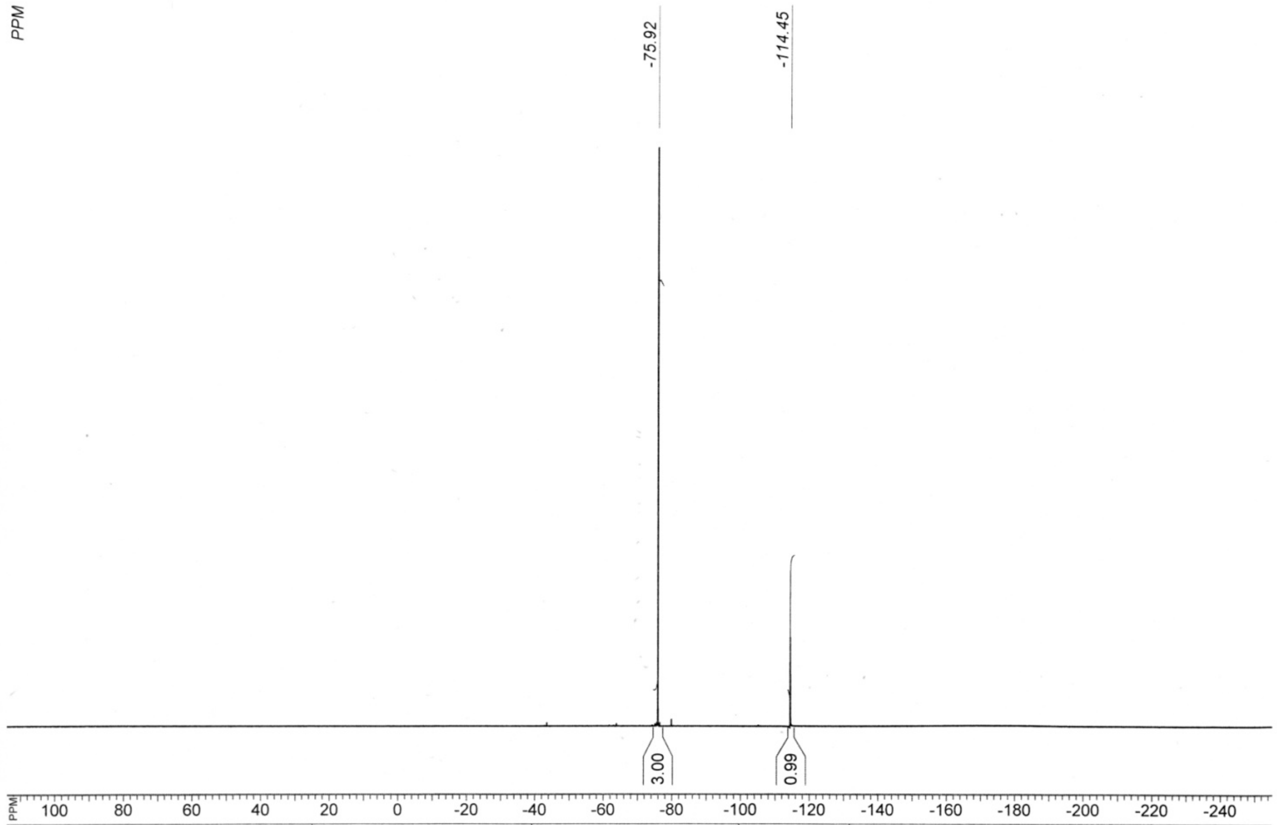

File name: UOFA19112_F19	Operator: USER 1	SF: 376.4986 MHz	NSC: 4	PW: 0.00 usec, RG: 2050	SI: 262144
Date: 02-Mar-2018	Solvent: CDCl3	SW: 138889 Hz	TE: 300 K	AQ: 0.94 sec, RD: 0.00 sec	

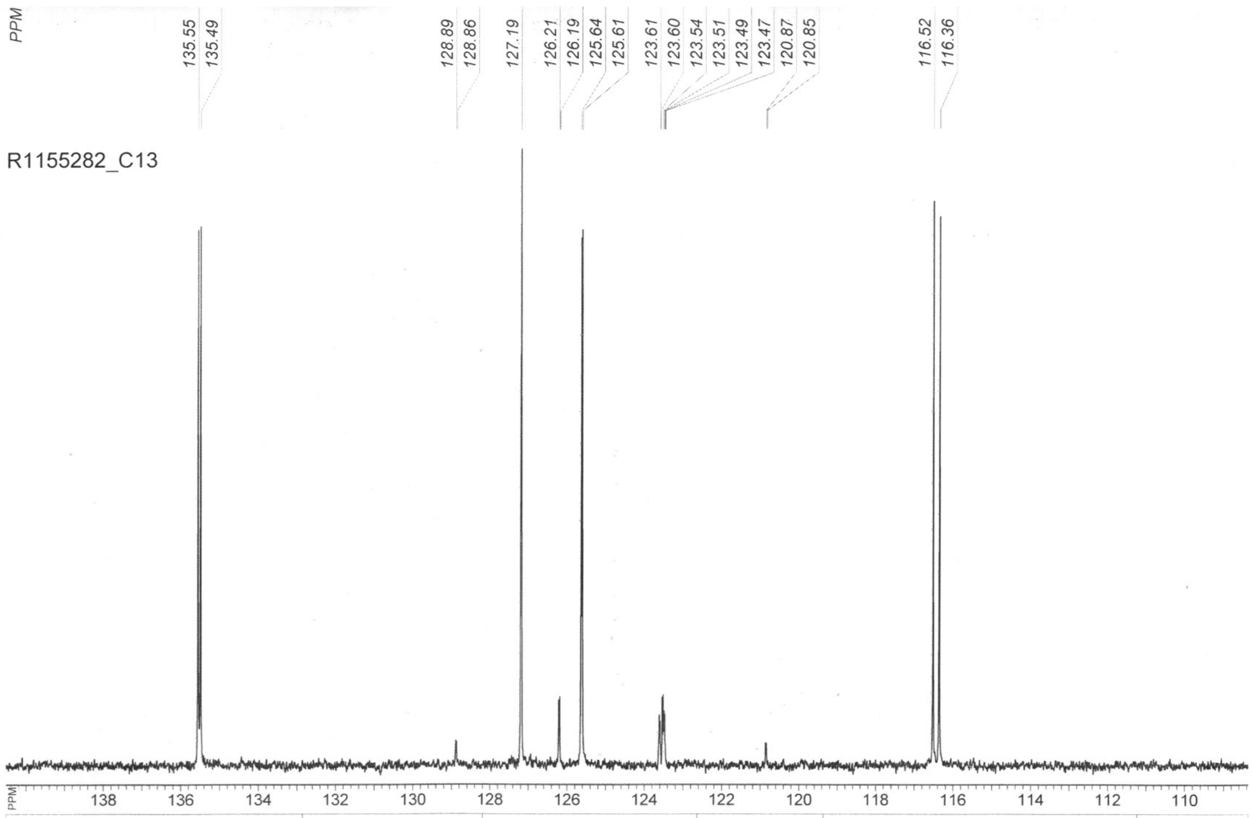

File name: R1155282_C13	Operator: root	SF: 125.6934 MHz	NSC: 1582	PW: 0.00 usec, RG: 51200	SI: 65536
Date: 25-Jul-2018	Solvent: CDCl3	SW: 32680 Hz	TE: 300 K	AQ: 1.57 sec, RD: 0.00 sec	

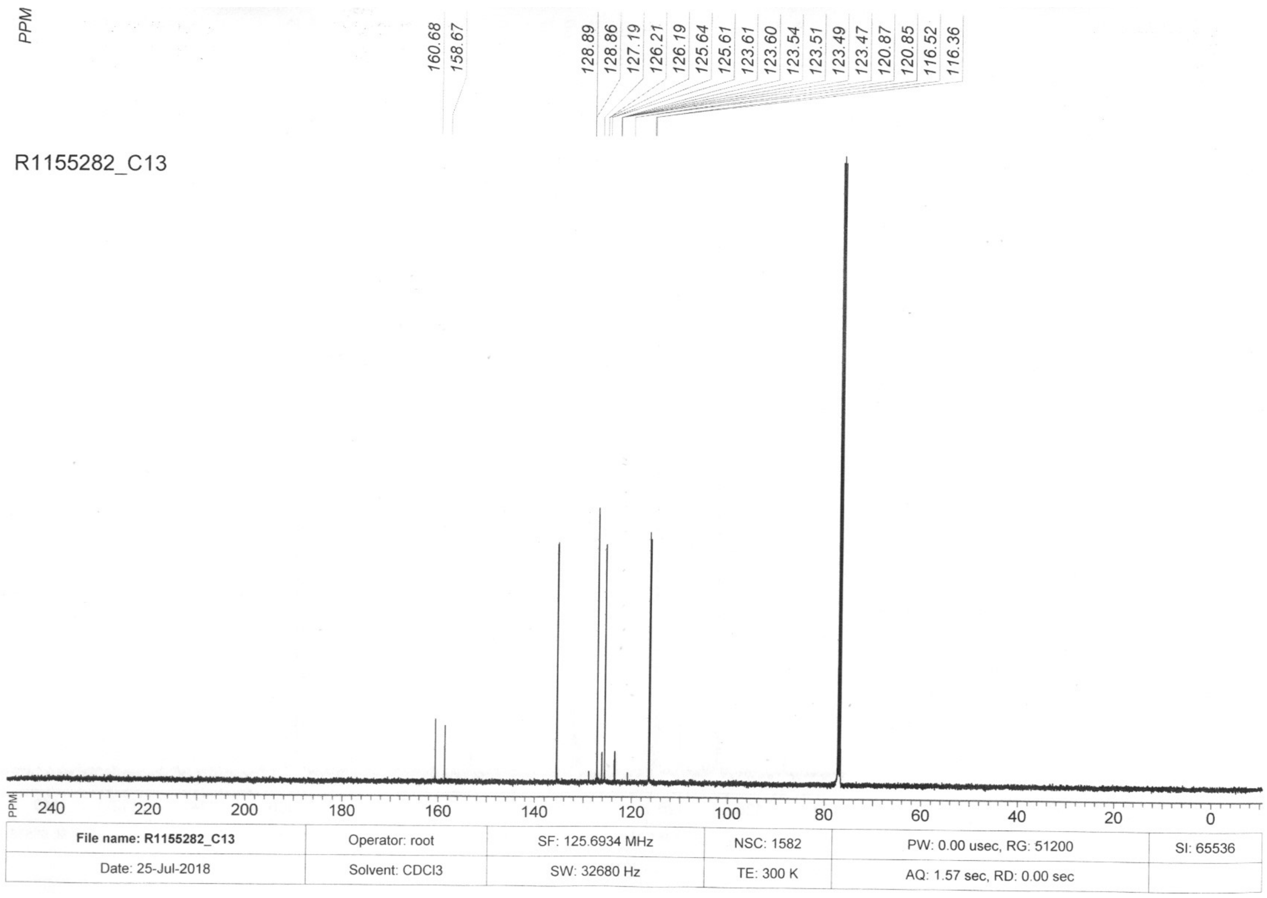
PPM
160.68
158.67
128.89
128.86
127.19
126.21
126.19
125.64
125.61
123.61
123.60
123.54
123.51
123.49
123.47
120.87
120.85
116.52
116.36
R1155282_C13
240 220 200 180 160 140 120 100 80 60 40 20 0
File name: R1155282_C13
Operator: root
SF: 125.6934 MHz
NSC: 1582
PW: 0.00 usec, RG: 51200
SI: 65536
Date: 25-Jul-2018
Solvent: CDCl3
SW: 32680 Hz
TE: 300 K
AQ: 1.57 sec, RD: 0.00 sec

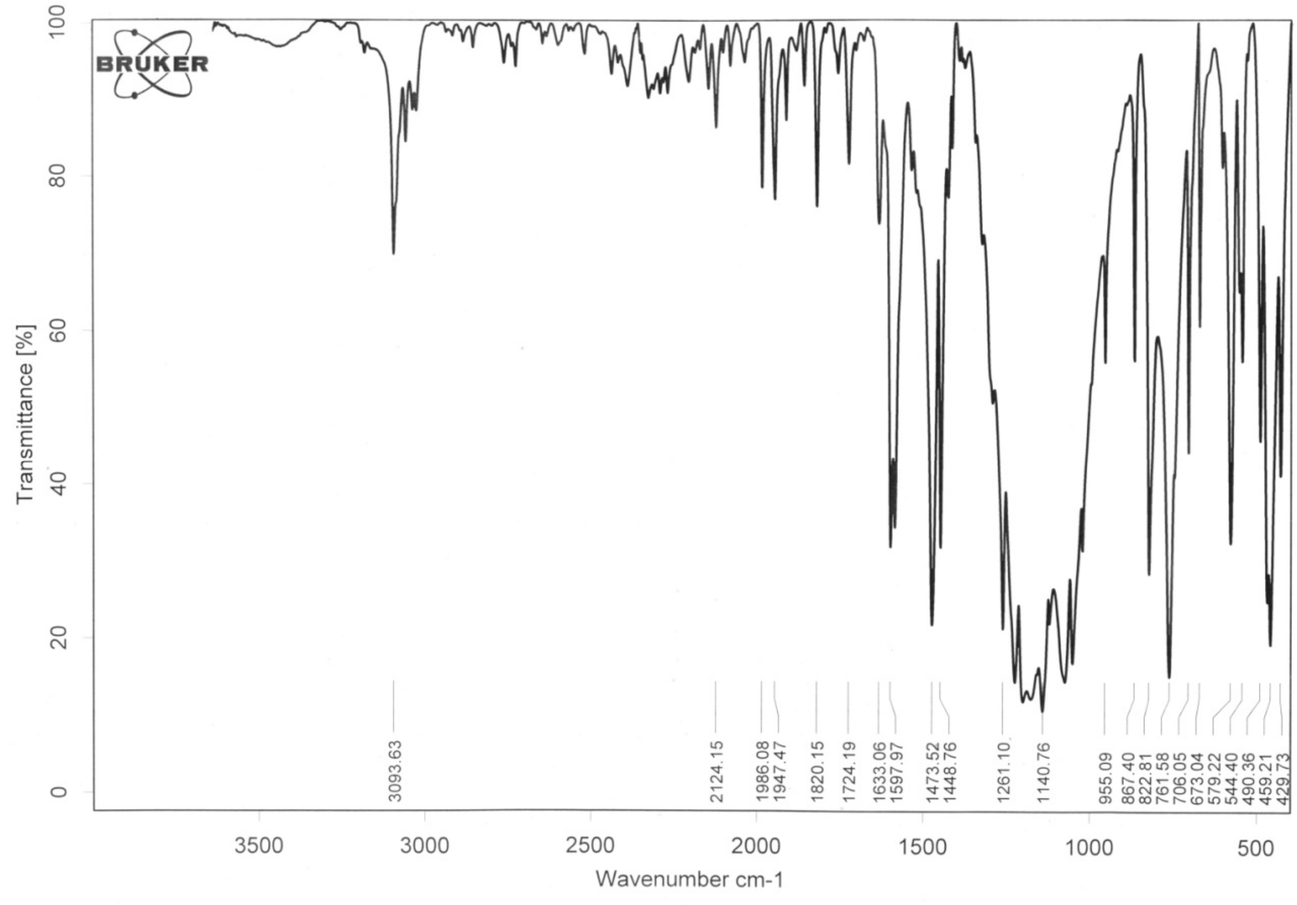
BRUKER
Transmittance [%]
0 20 40 60 80 100
3093.63
2124.15
1986.08
1947.47
1820.15
1724.19
1633.06
1597.97
1473.52
1448.76
1261.10
1140.76
955.09
867.40
822.81
761.58
706.05
673.04
579.22
544.40
490.36
459.21
429.73
3500 3000 2500 2000 1500 1000 500
Wavenumber cm-1

2-((Trifluoromethyl)sulfinyl)-acetophenone.

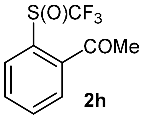

2h

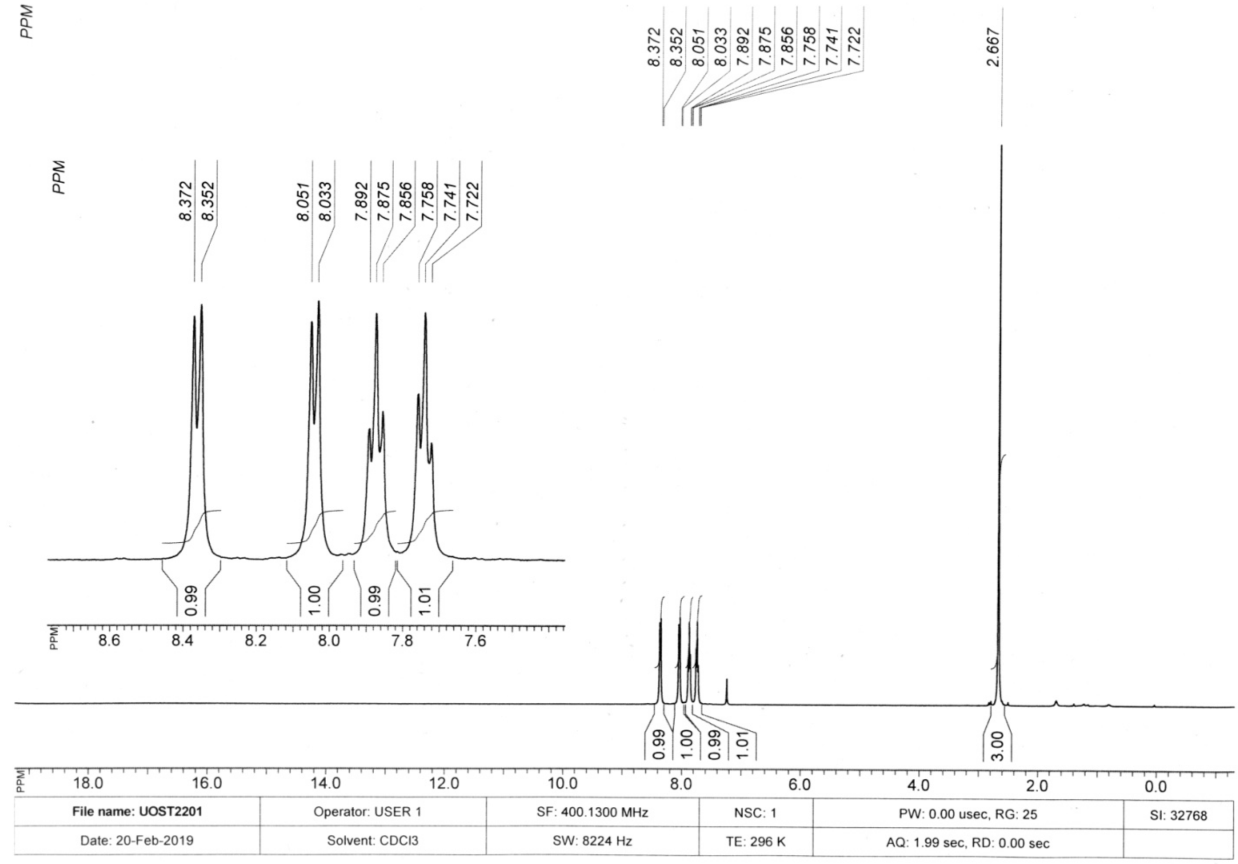

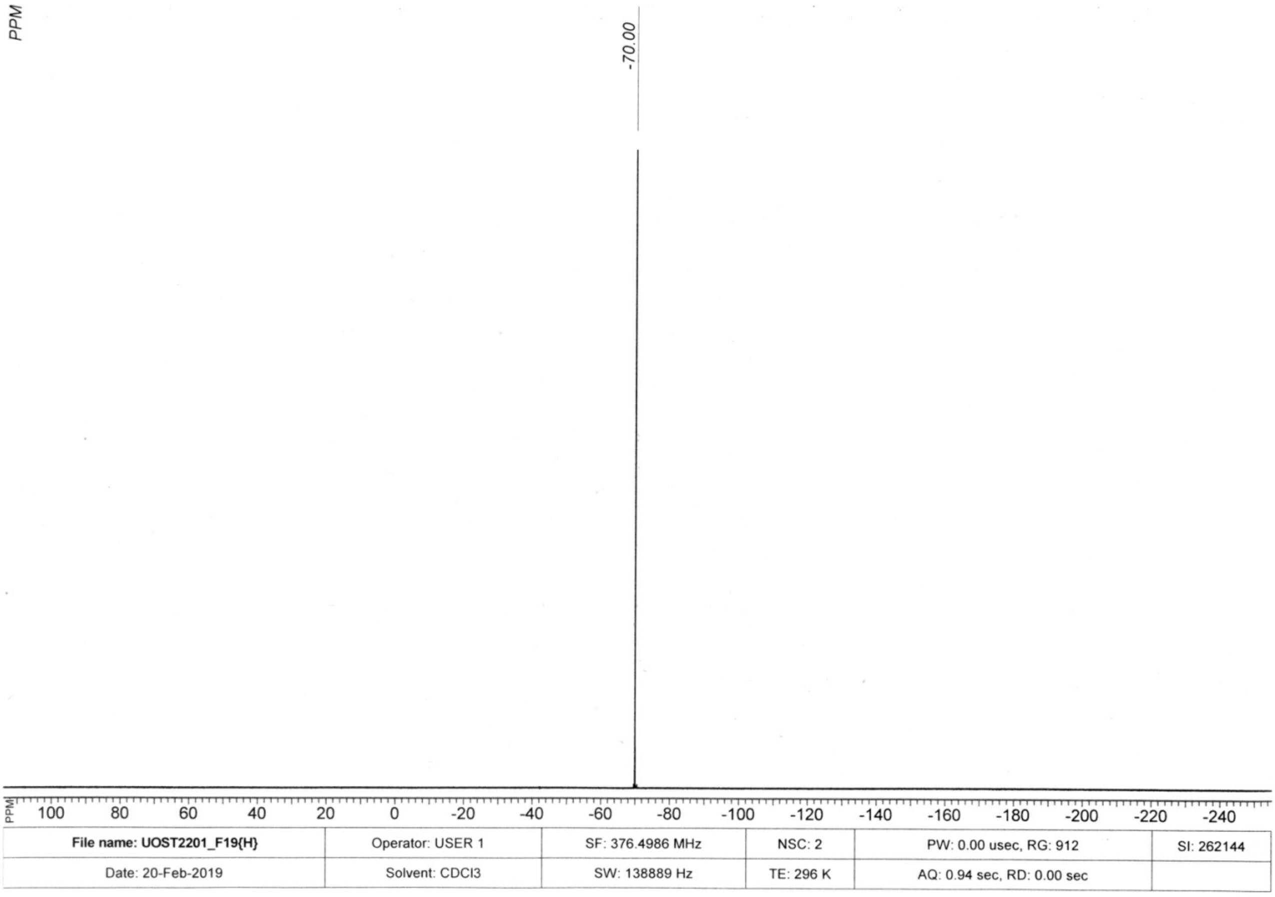

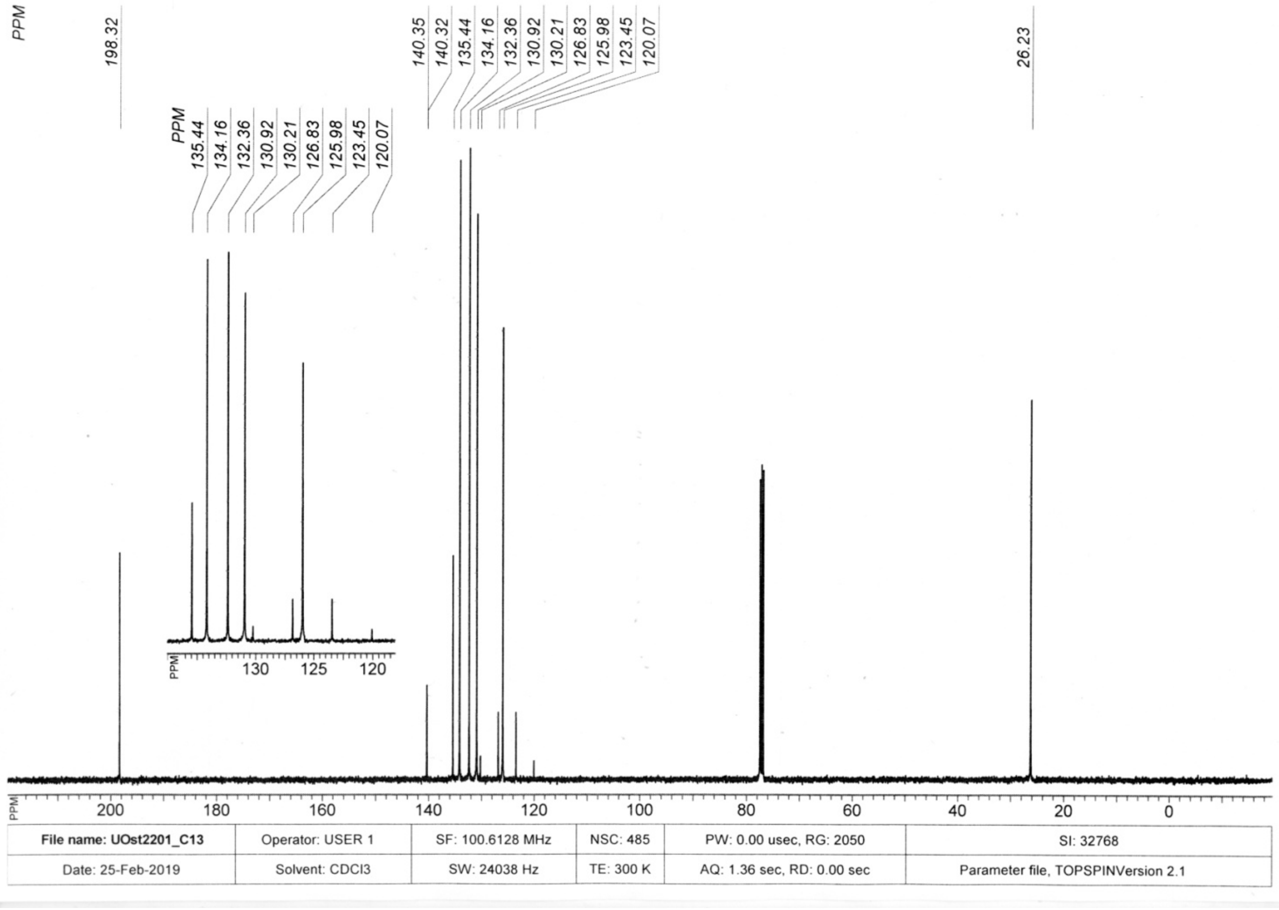
PPM
198.32
140.35
140.32
135.44
134.16
132.36
130.92
130.21
126.83
125.98
123.45
120.07
26.23
PPM
135.44
134.16
132.36
130.92
130.21
126.83
125.98
123.45
120.07
PPM 130 125 120
PPM 200 180 160 140 120 100 80 60 40 20 0
File name: UOst2201_C13
Operator: USER 1
SF: 100.6128 MHz
NSC: 485
PW: 0.00 usec, RG: 2050
SI: 32768
Date: 25-Feb-2019
Solvent: CDCl3
SW: 24038 Hz
TE: 300 K
AQ: 1.36 sec, RD: 0.00 sec
Parameter file, TOPSPINVersion 2.1

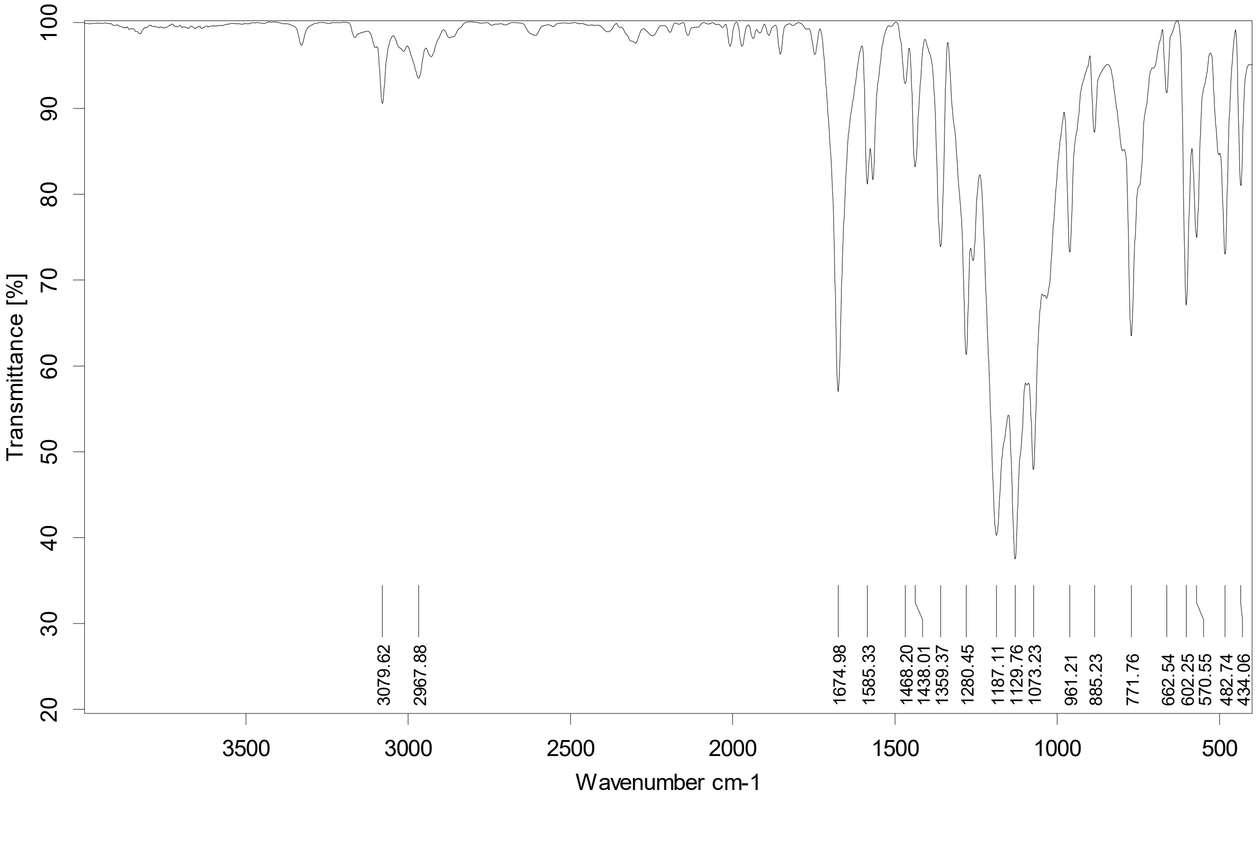
Transmittance [%]
100 90 80 70 60 50 40 30 20
3079.62
2967.88
1674.98
1585.33
1468.20
1438.01
1359.37
1280.45
1187.11
1129.76
1073.23
961.21
885.23
771.76
662.54
602.25
570.55
482.74
434.06
3500 3000 2500 2000 1500 1000 500
Wavenumber cm-1

2-((Trifluoromethyl)sulfinyl)phenyl acetate 2i.

Product after column chromatography. Contains ~15 % of corresponding phenol.

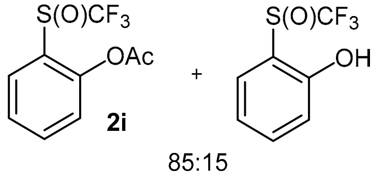

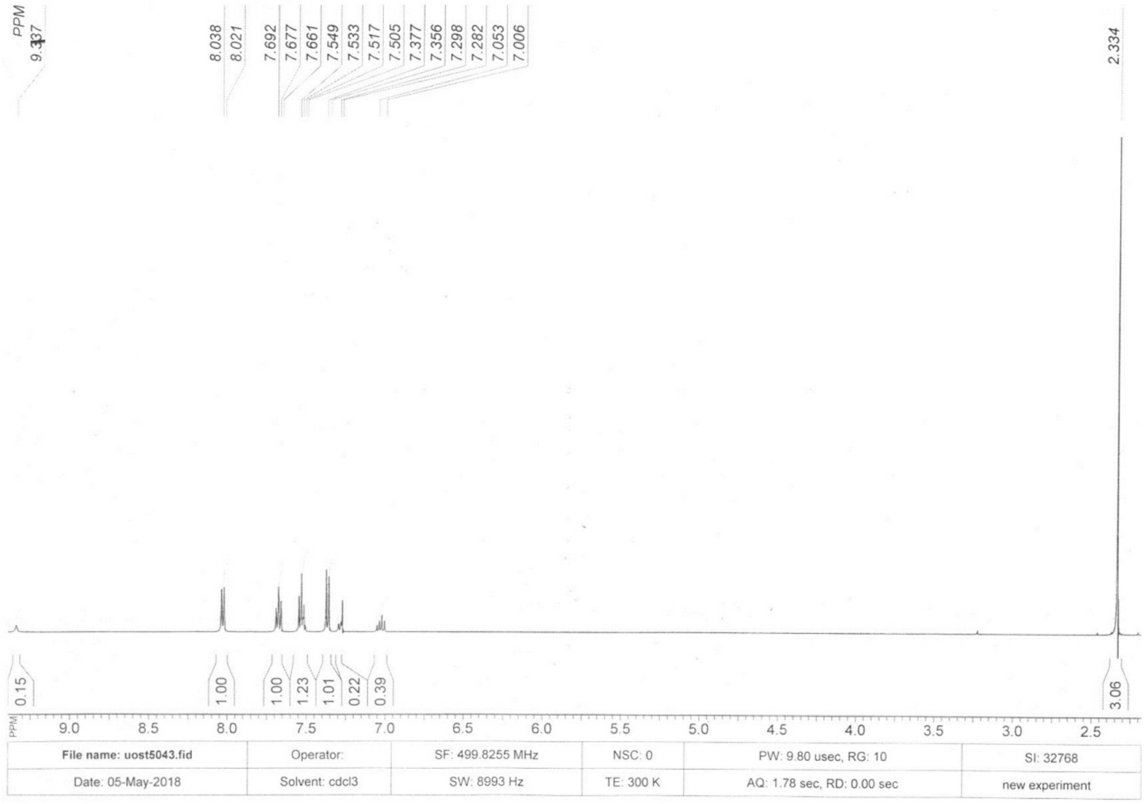

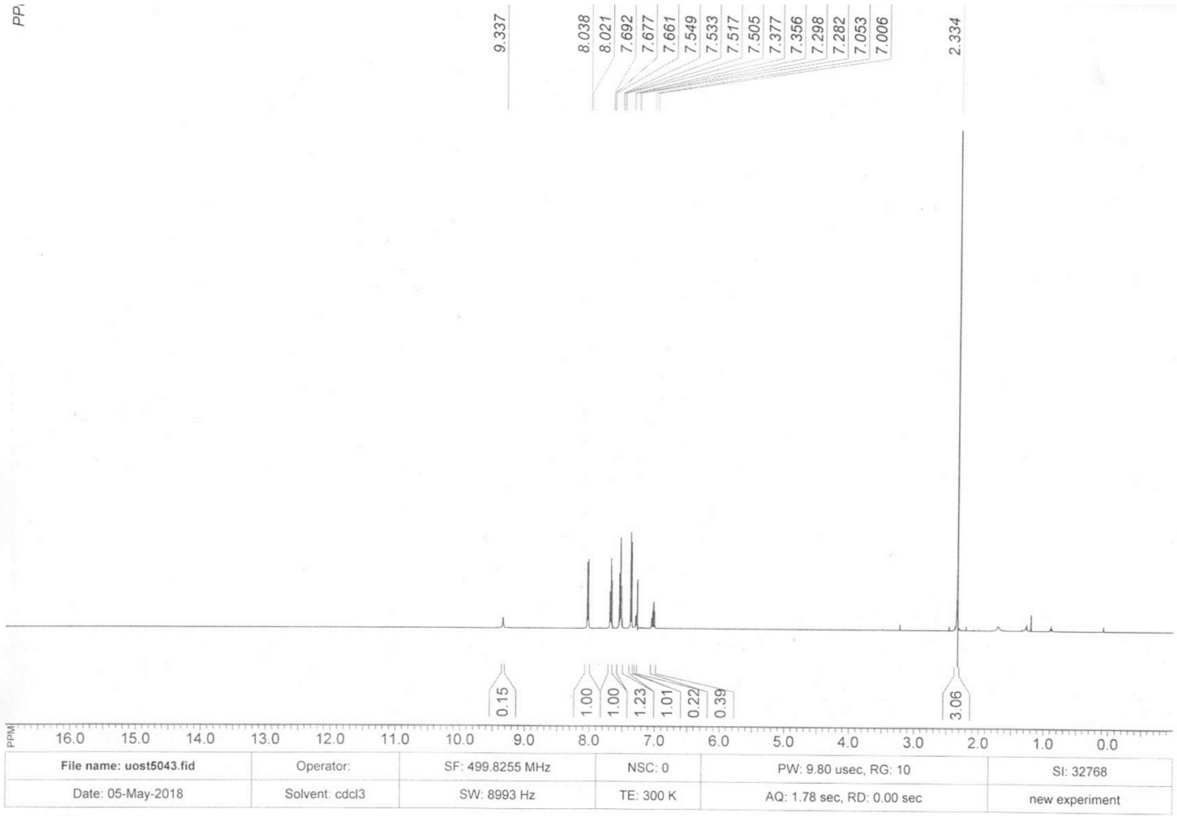

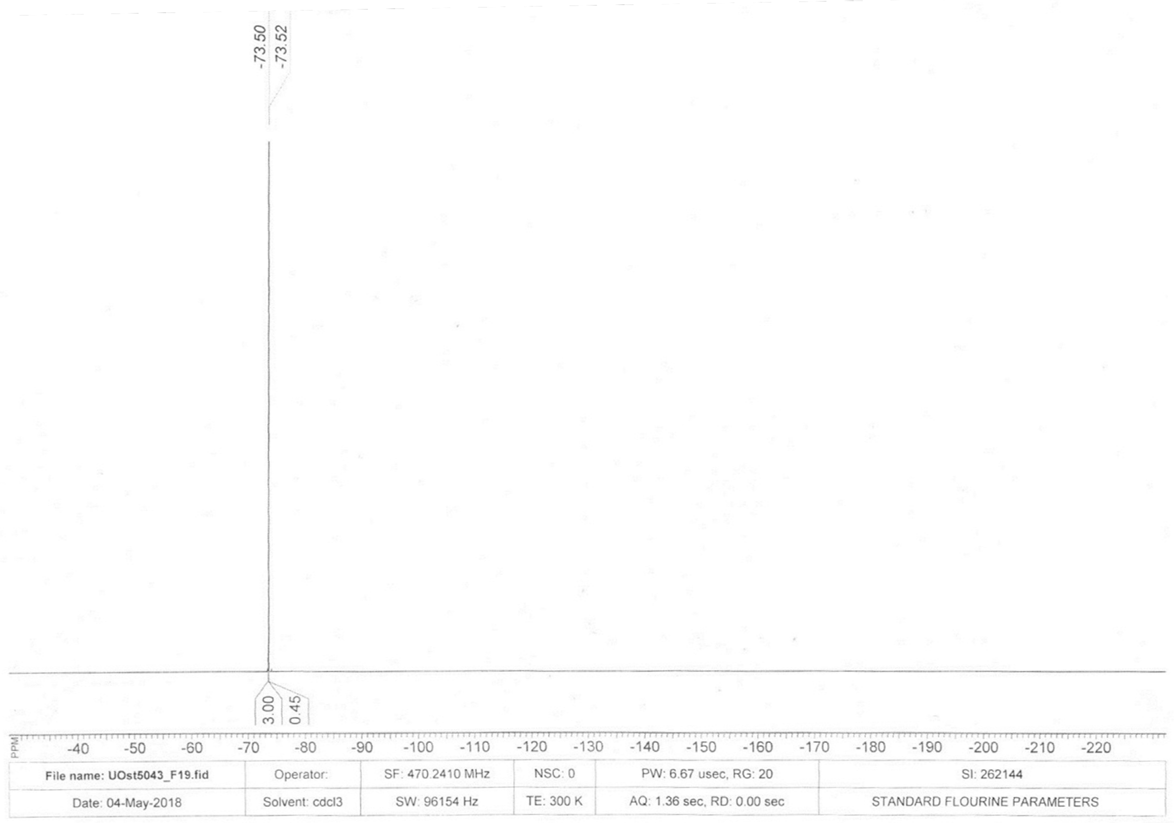
-73.50
-73.52
3.00
0.45
PPM
-40 -50 -60 -70 -80 -90 -100 -110 -120 -130 -140 -150 -160 -170 -180 -190 -200 -210 -220
File name: UOst5043_F19.fid
Operator:
SF: 470.2410 MHz
NSC: 0
PW: 6.67 usec, RG: 20
SI: 262144
Date: 04-May-2018
Solvent: cdcl3
SW: 96154 Hz
TE: 300 K
AQ: 1.36 sec, RD: 0.00 sec
STANDARD FLOURINE PARAMETERS

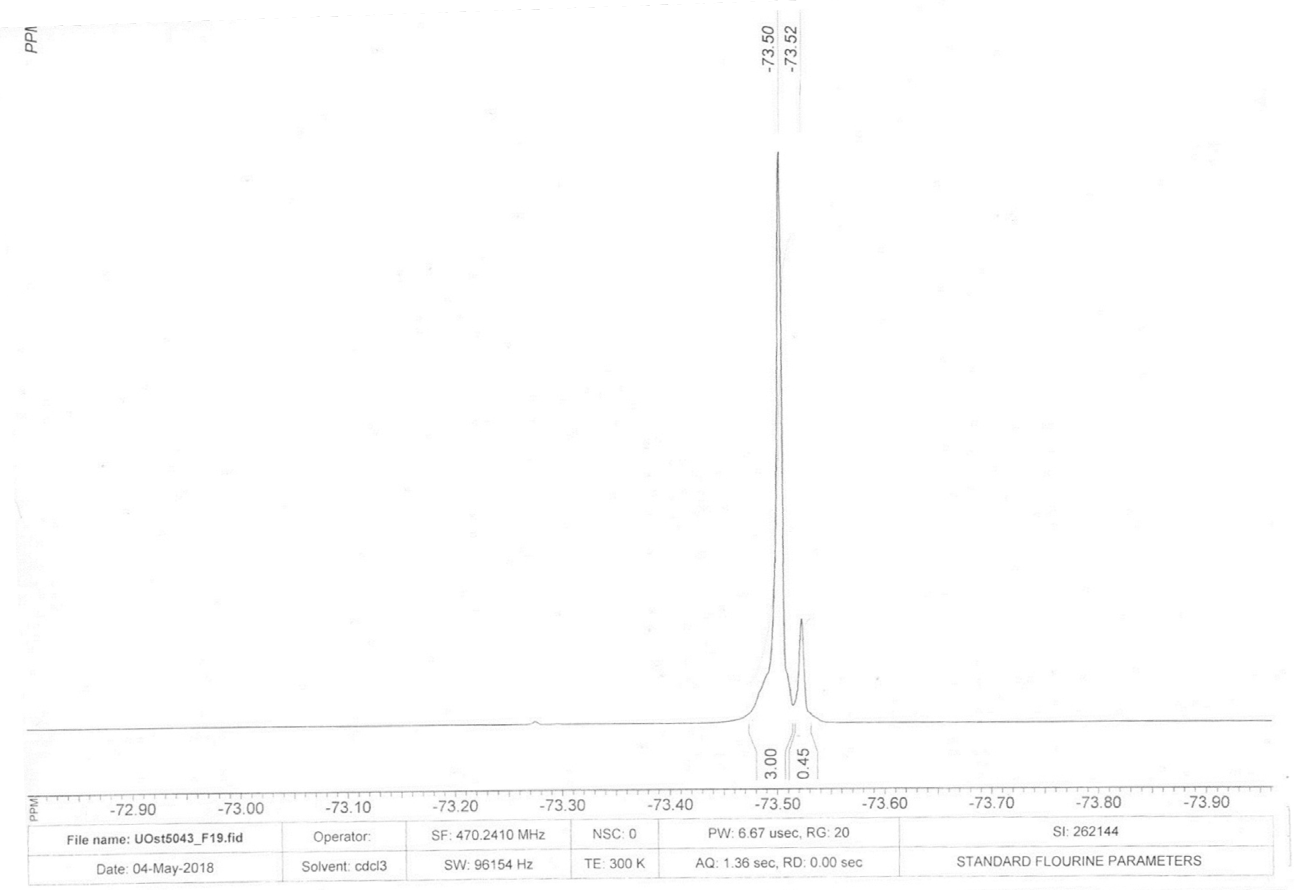
PPM
-73.50
-73.52
3.00
0.45
-72.90 -73.00 -73.10 -73.20 -73.30 -73.40 -73.50 -73.60 -73.70 -73.80 -73.90
File name: UOst5043_F19.fid
Operator:
SF: 470.2410 MHz
NSC: 0
PW: 6.67 usec, RG: 20
SI: 262144
Date: 04-May-2018
Solvent: cdcl3
SW: 96154 Hz
TE: 300 K
AQ: 1.36 sec, RD: 0.00 sec
STANDARD FLOURINE PARAMETERS

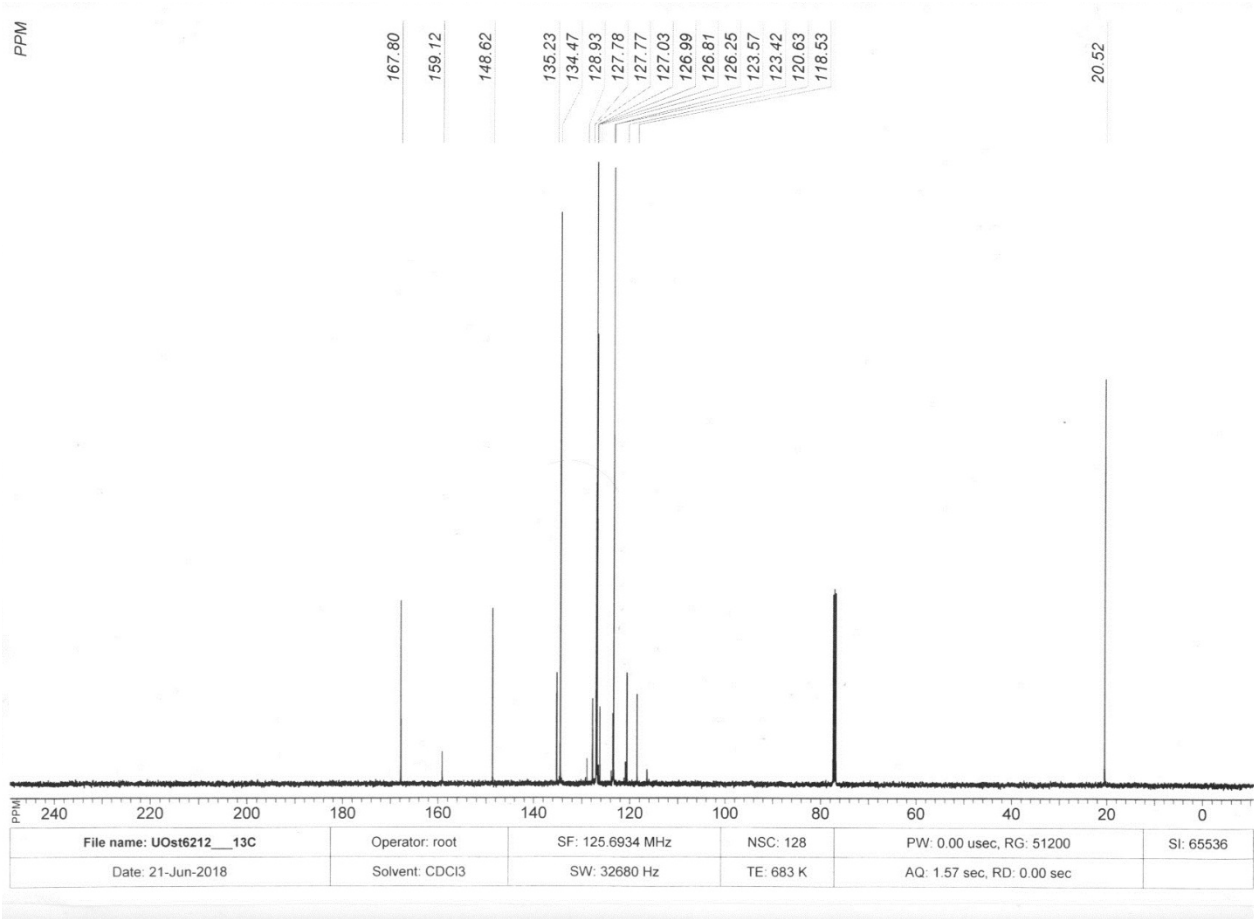
PPM
167.80
159.12
148.62
135.23
134.47
128.93
127.78
127.77
127.03
126.99
126.81
126.25
123.57
123.42
120.63
118.53
20.52
240 220 200 180 160 140 120 100 80 60 40 20 0
File name: UOst6212___13C
Date: 21-Jun-2018
Operator: root
Solvent: CDCl3
SF: 125.6934 MHz
SW: 32680 Hz
NSC: 128
TE: 683 K
PW: 0.00 usec, RG: 51200
AQ: 1.57 sec, RD: 0.00 sec
SI: 65536

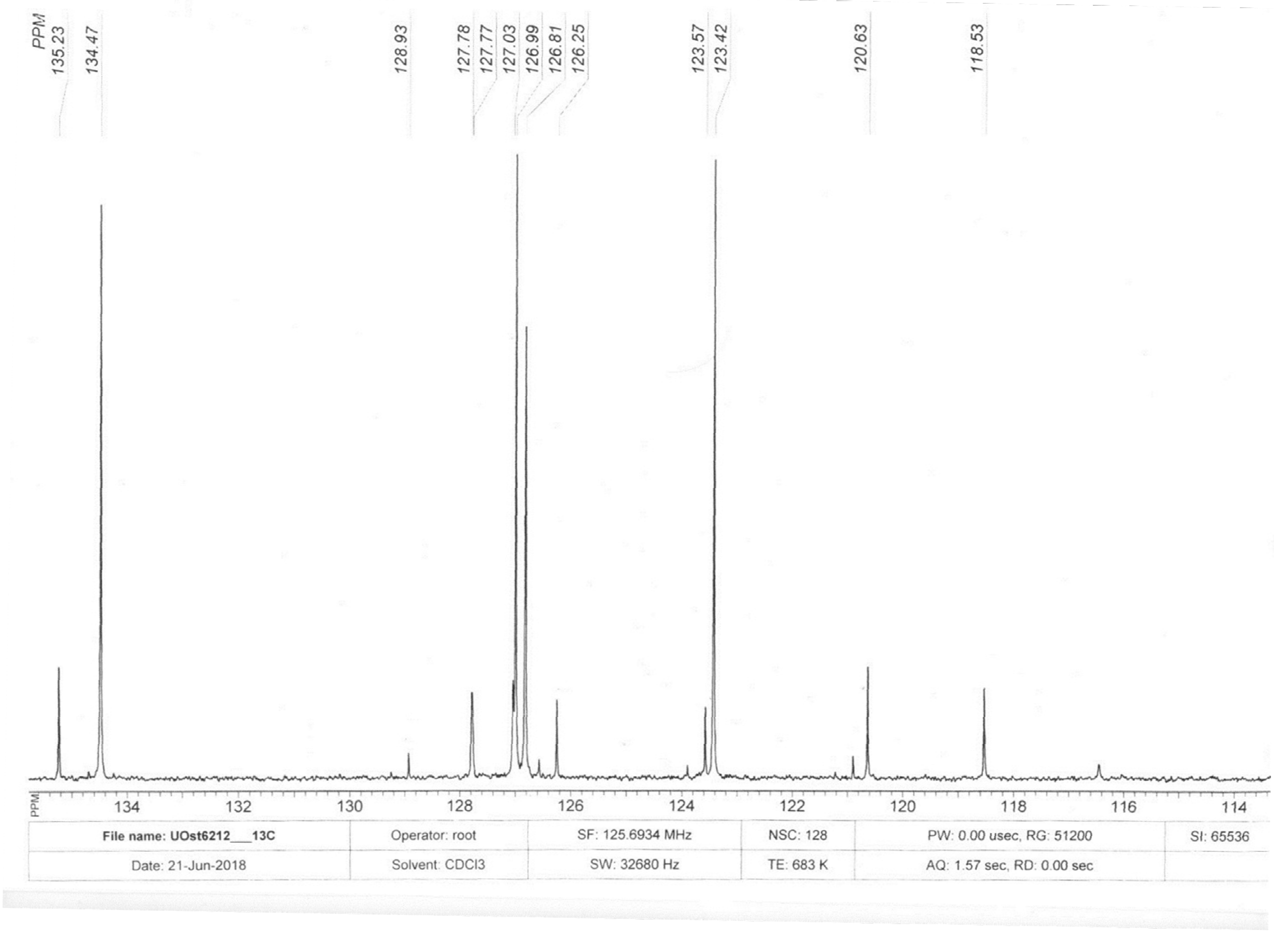
PPM
135.23
134.47
128.93
127.78
127.77
127.03
126.99
126.81
126.25
123.57
123.42
120.63
118.53
134 132 130 128 126 124 122 120 118 116 114
File name: UOst6212___13C
Date: 21-Jun-2018
Operator: root
Solvent: CDCl3
SF: 125.6934 MHz
SW: 32680 Hz
NSC: 128
TE: 683 K
PW: 0.00 usec, RG: 51200
AQ: 1.57 sec, RD: 0.00 sec
SI: 65536

2,4-Dinitro-1-((trifluoromethyl)sulfinyl)benzene.

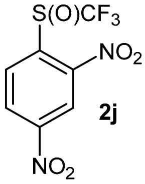

2j

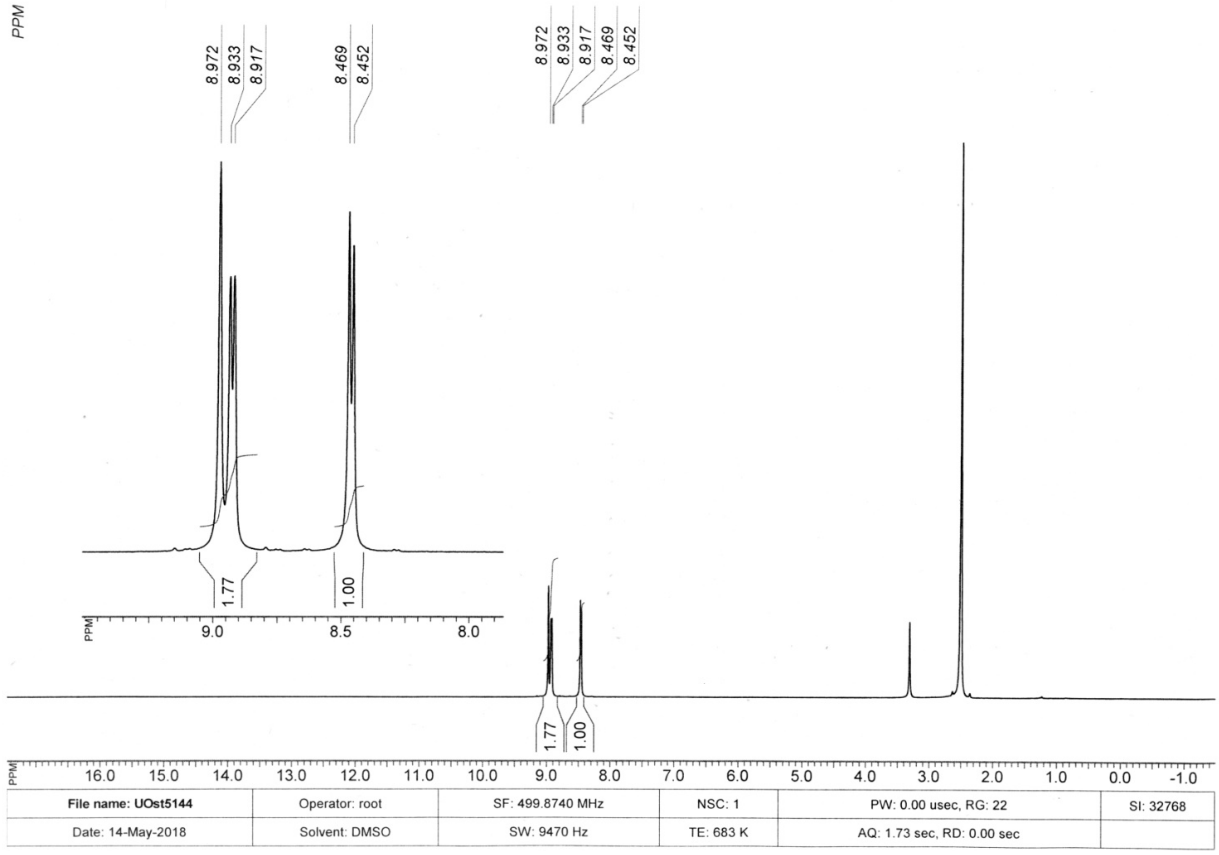

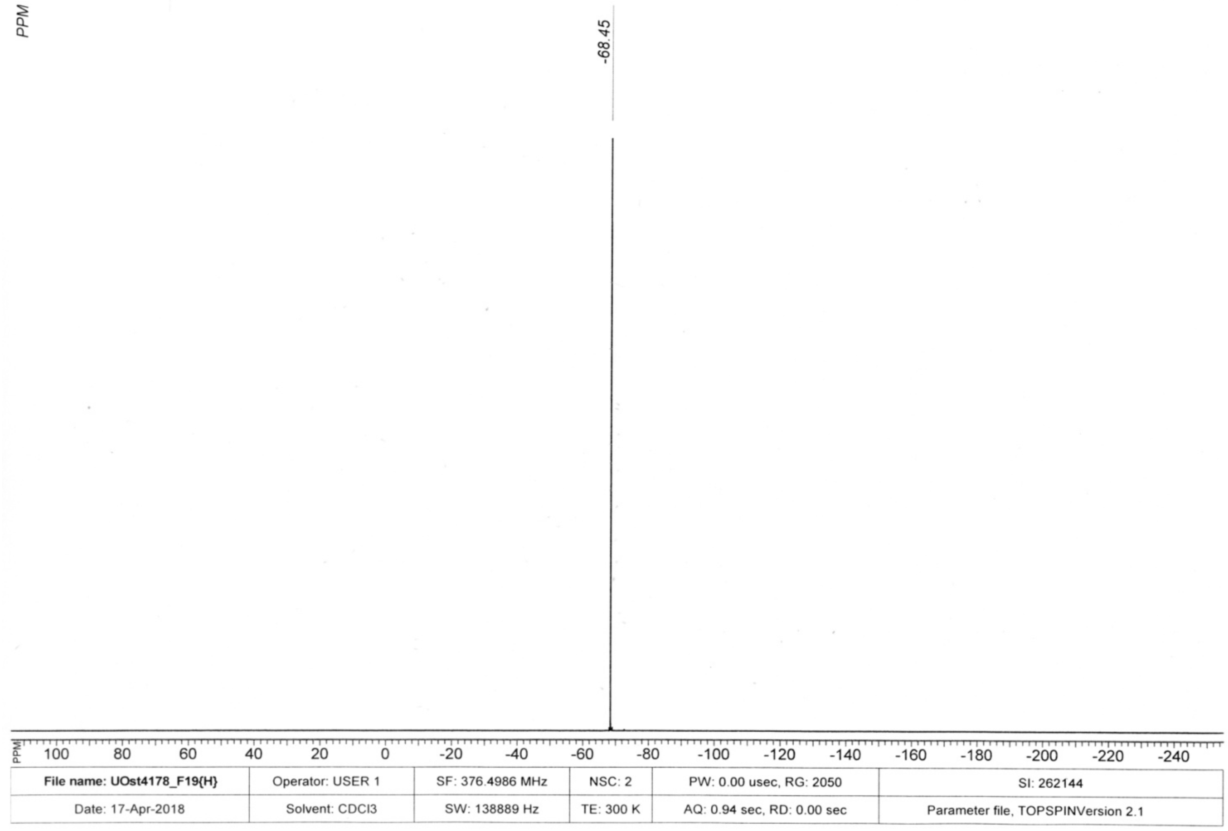

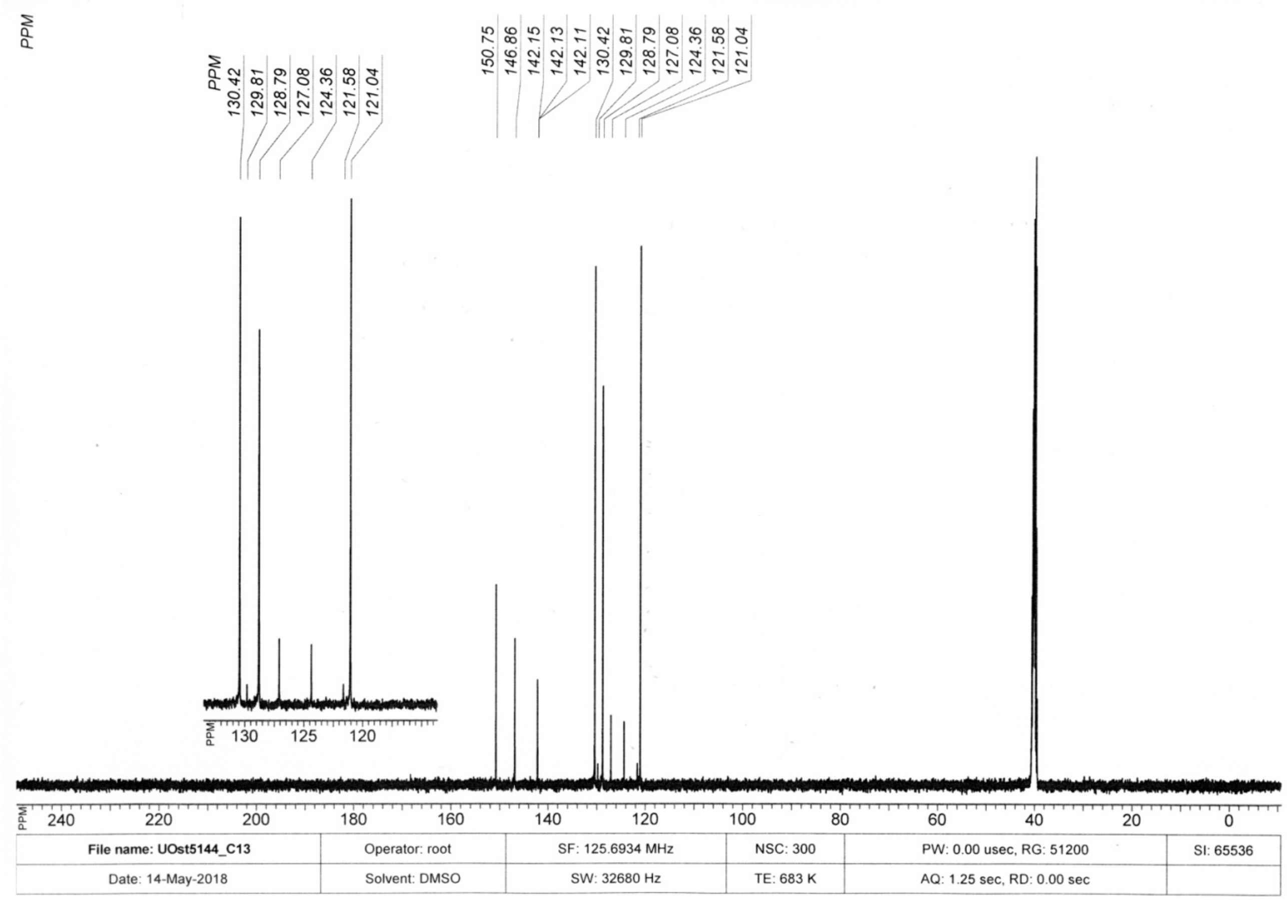
PPM
150.75
146.86
142.15
142.13
142.11
130.42
129.81
128.79
127.08
124.36
121.58
121.04
File name: UOst5144_C13
Date: 14-May-2018
Operator: root
Solvent: DMSO
SF: 125.6934 MHz
SW: 32680 Hz
NSC: 300
TE: 683 K
PW: 0.00 usec, RG: 51200
AQ: 1.25 sec, RD: 0.00 sec
SI: 65536

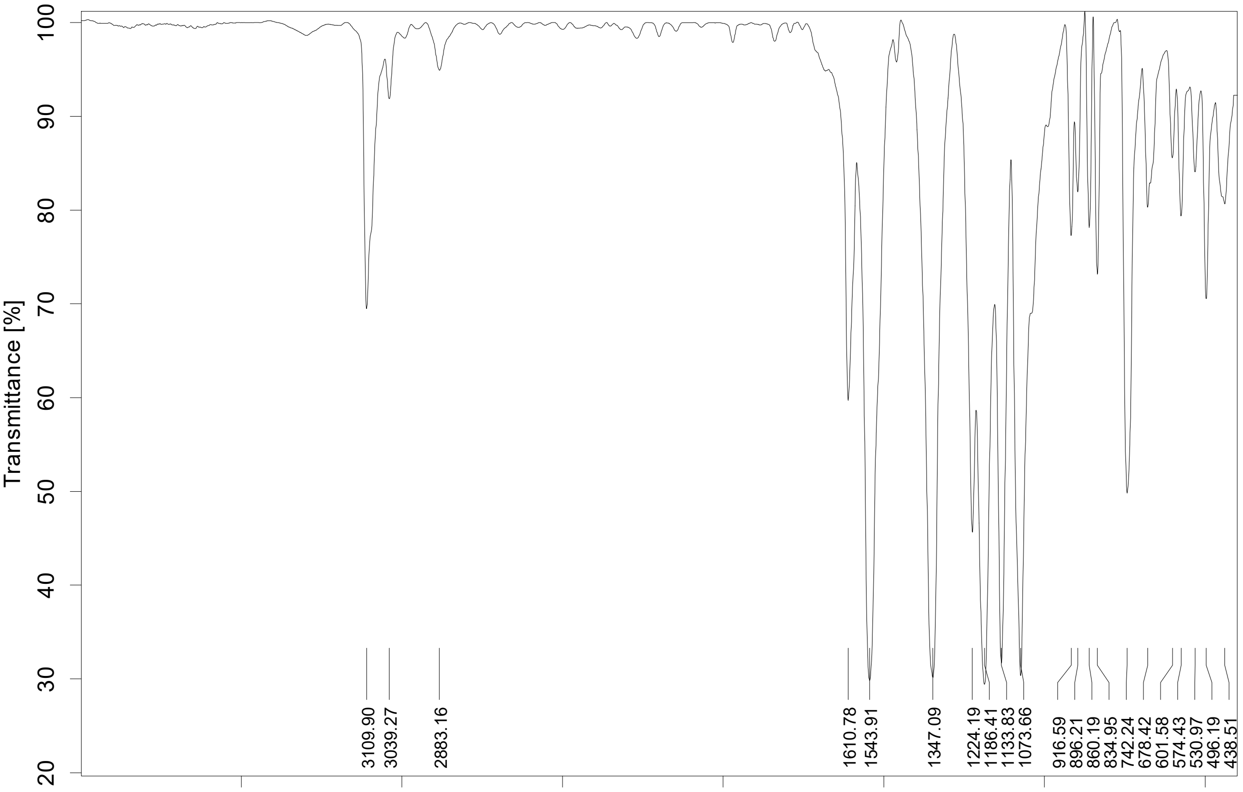
Transmittance [%]
Wavenumber cm-1
3109.90
3039.27
2883.16
1610.78
1543.91
1347.09
1224.19
1186.41
1133.83
1073.66
916.59
896.21
860.19
834.95
742.24
678.42
601.58
574.43
530.97
496.19
438.51

2-((Trifluoromethyl)sulfinyl)naphthalene 2k.

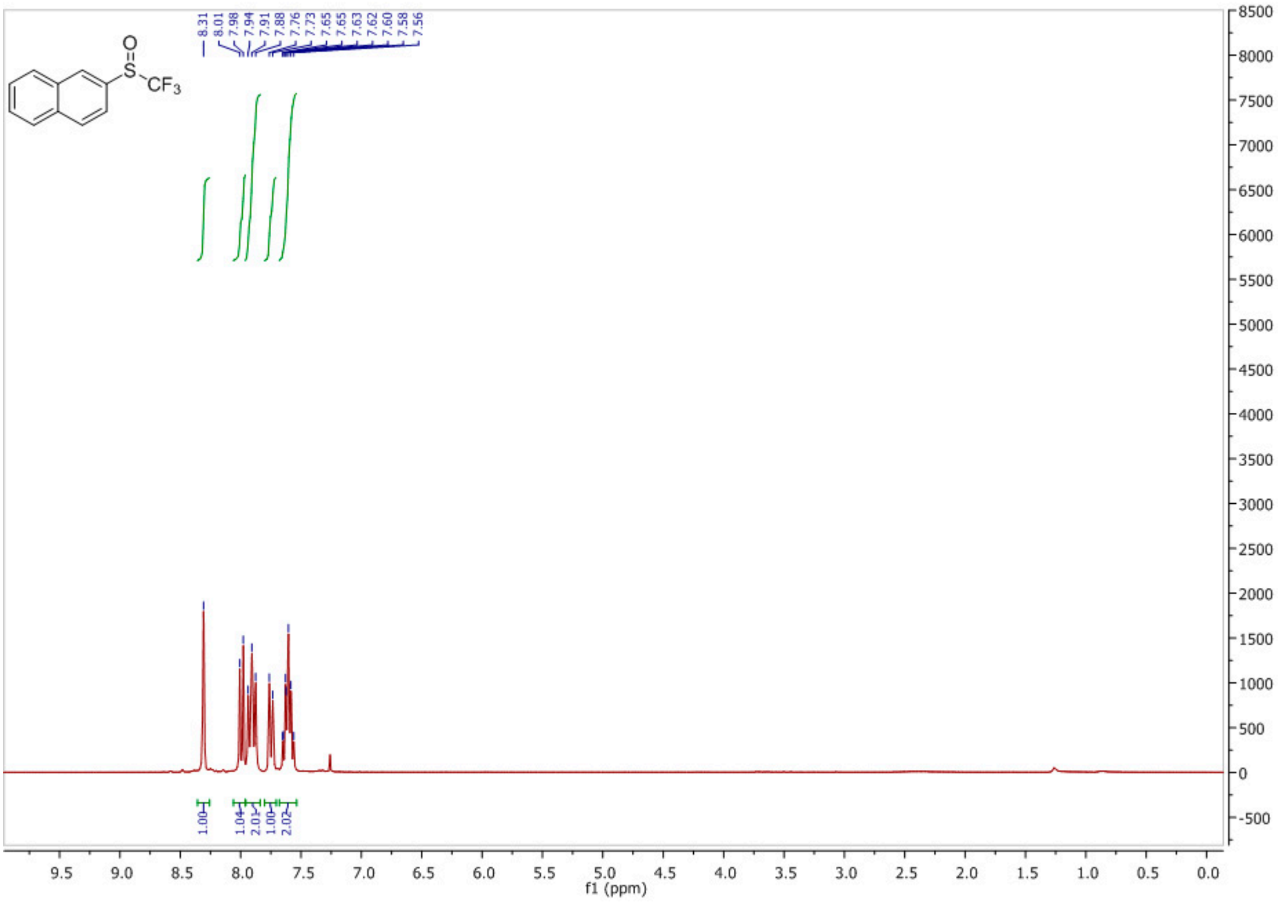

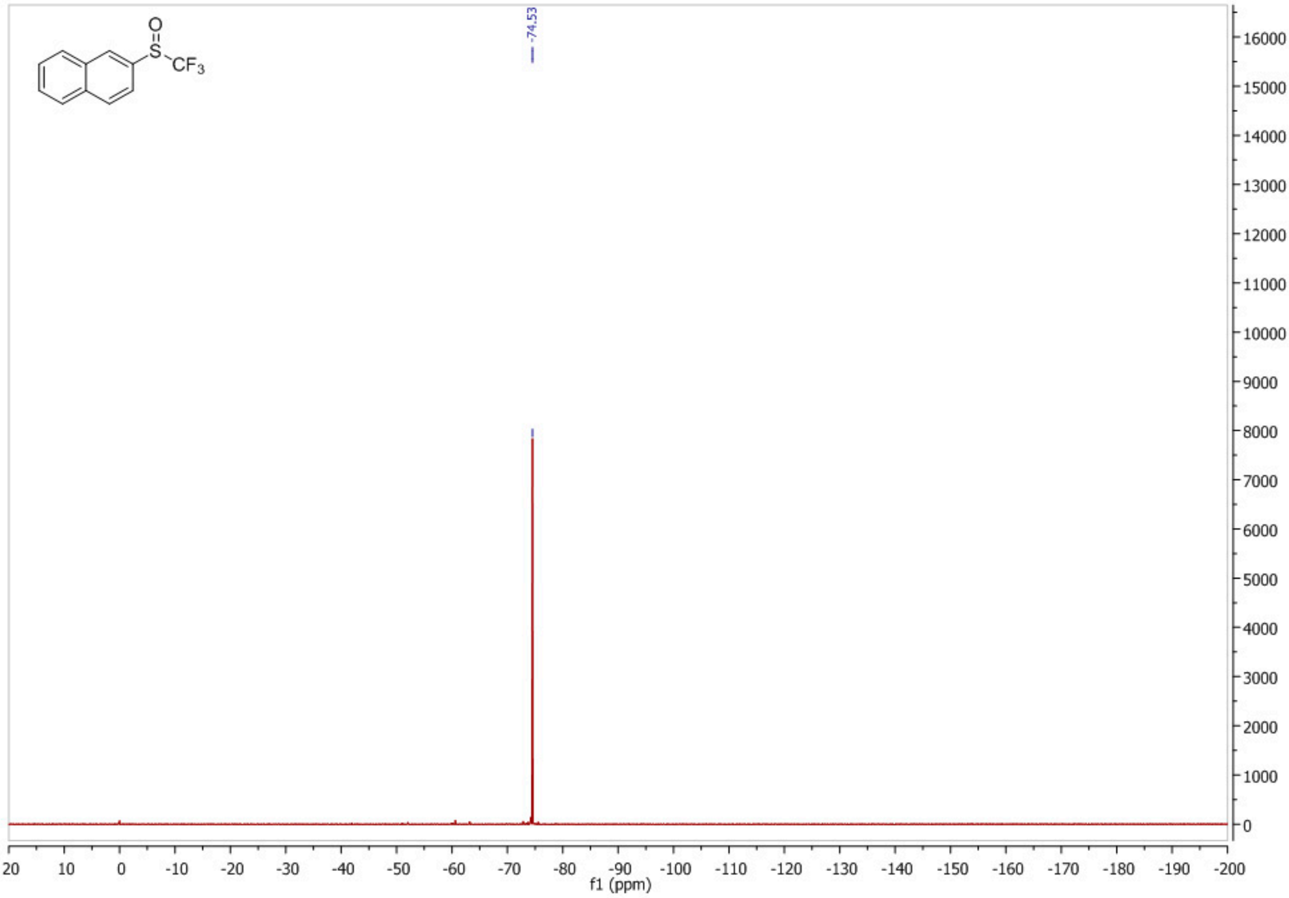

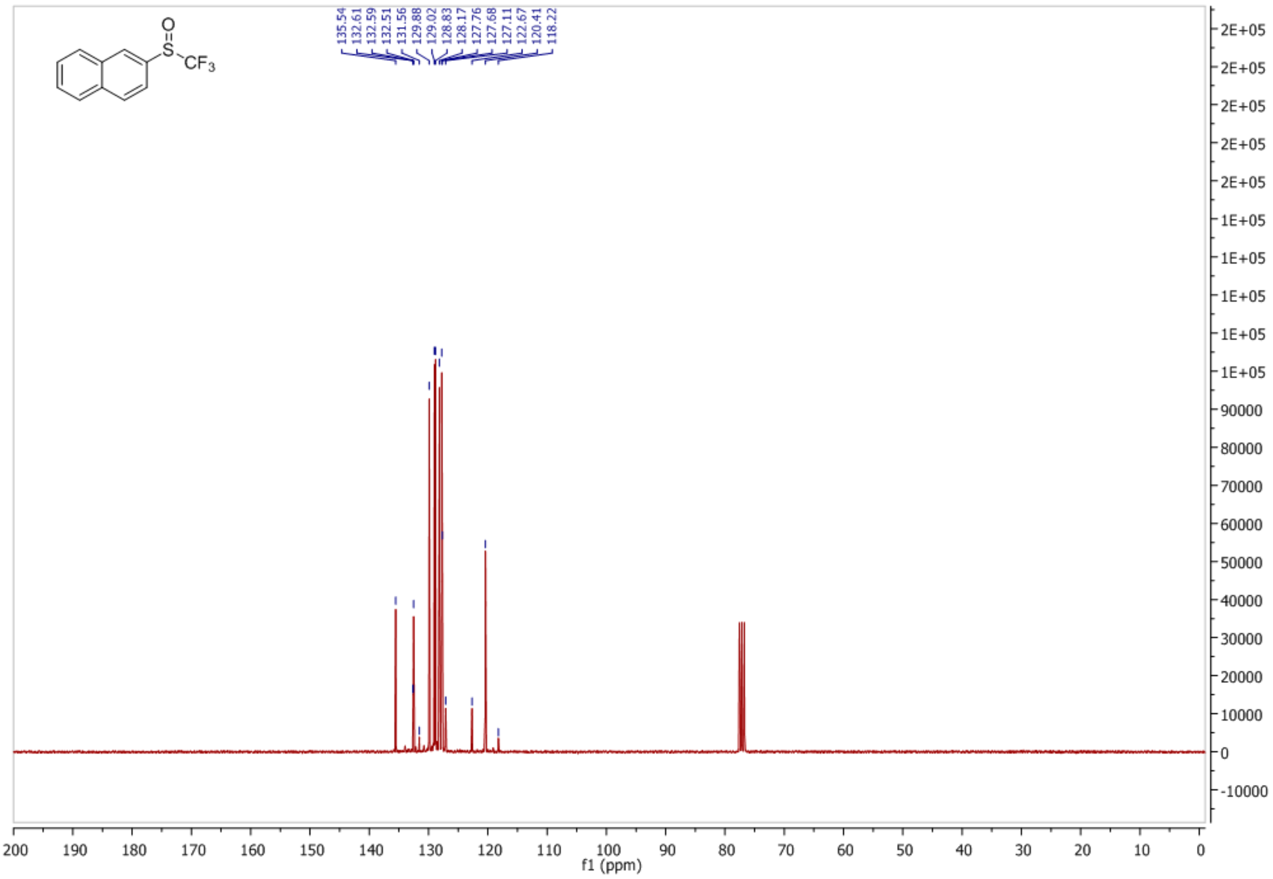
135.54
132.61
132.59
132.51
131.56
129.88
129.02
128.83
128.17
127.76
127.68
127.11
122.67
120.41
118.22
f1 (ppm)

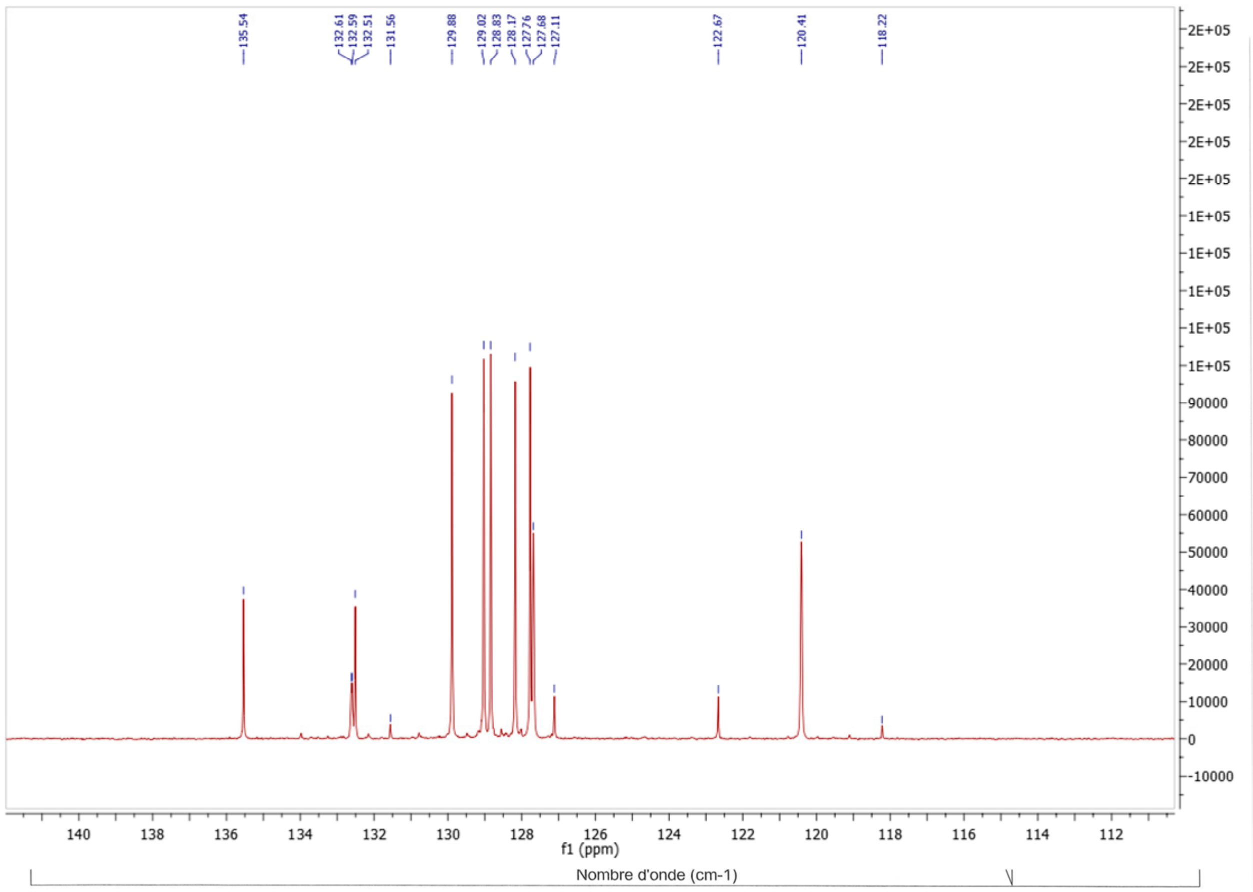
135.54
132.61
132.59
132.51
131.56
129.88
129.02
128.83
128.17
127.76
127.68
127.11
122.67
120.41
118.22
f1 (ppm)
Nombre d'onde (cm-1)

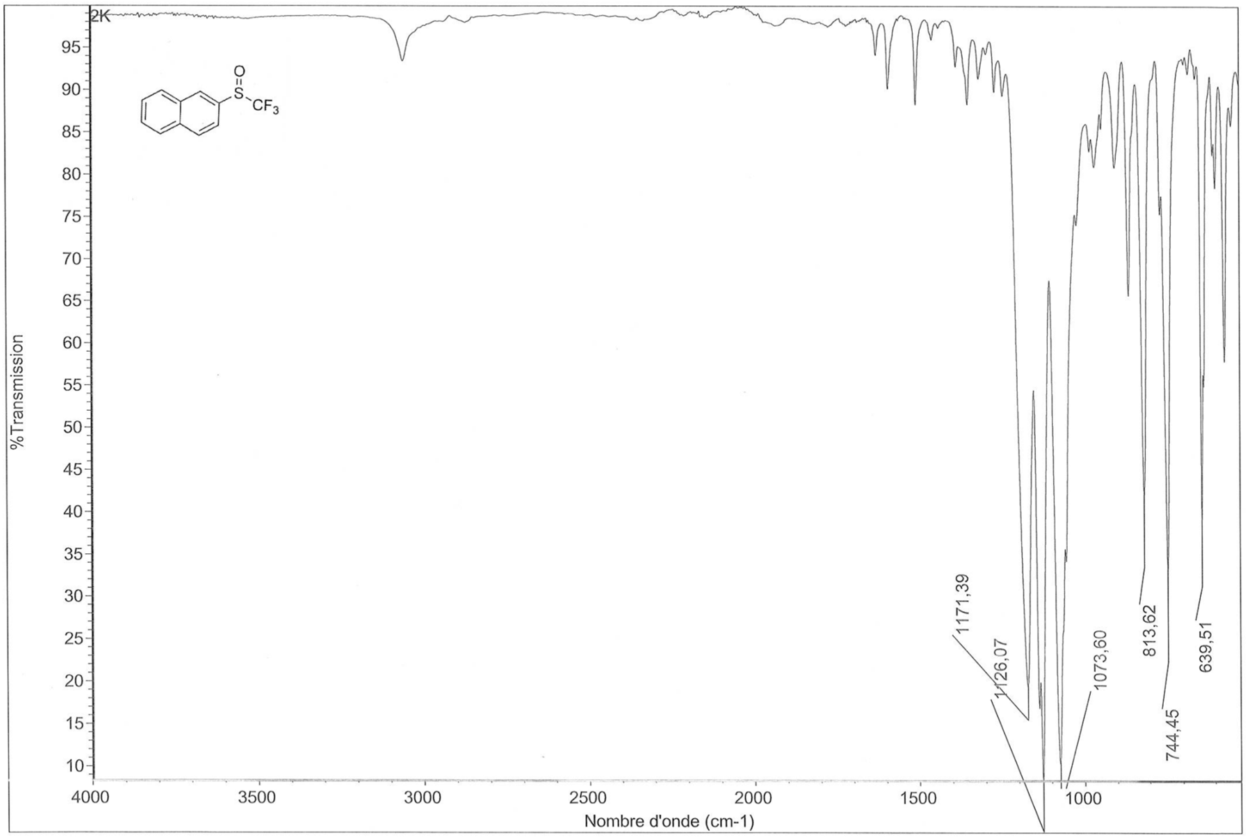

2-(Trifluoromethyl)sulfinyl-ethanol.

Contain ~15 % of starting sulfide.

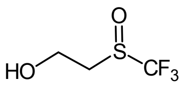

4b

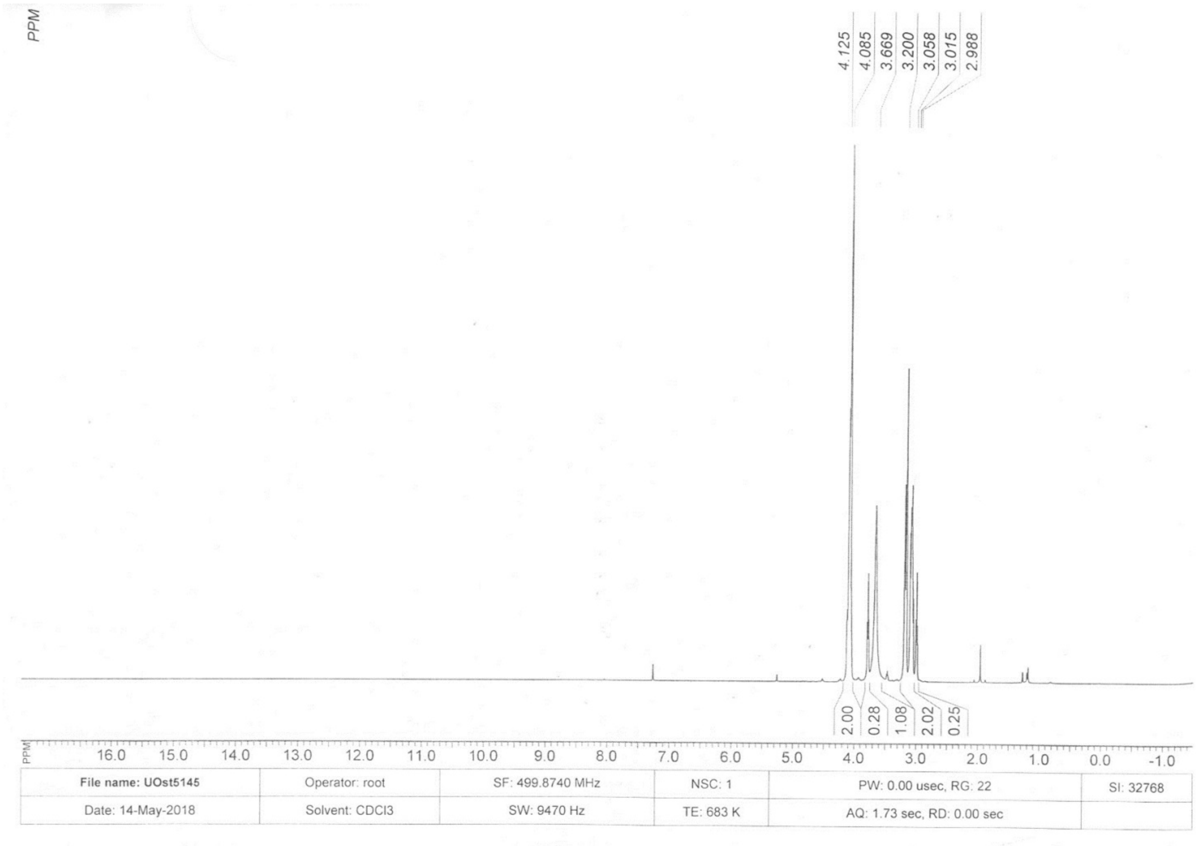

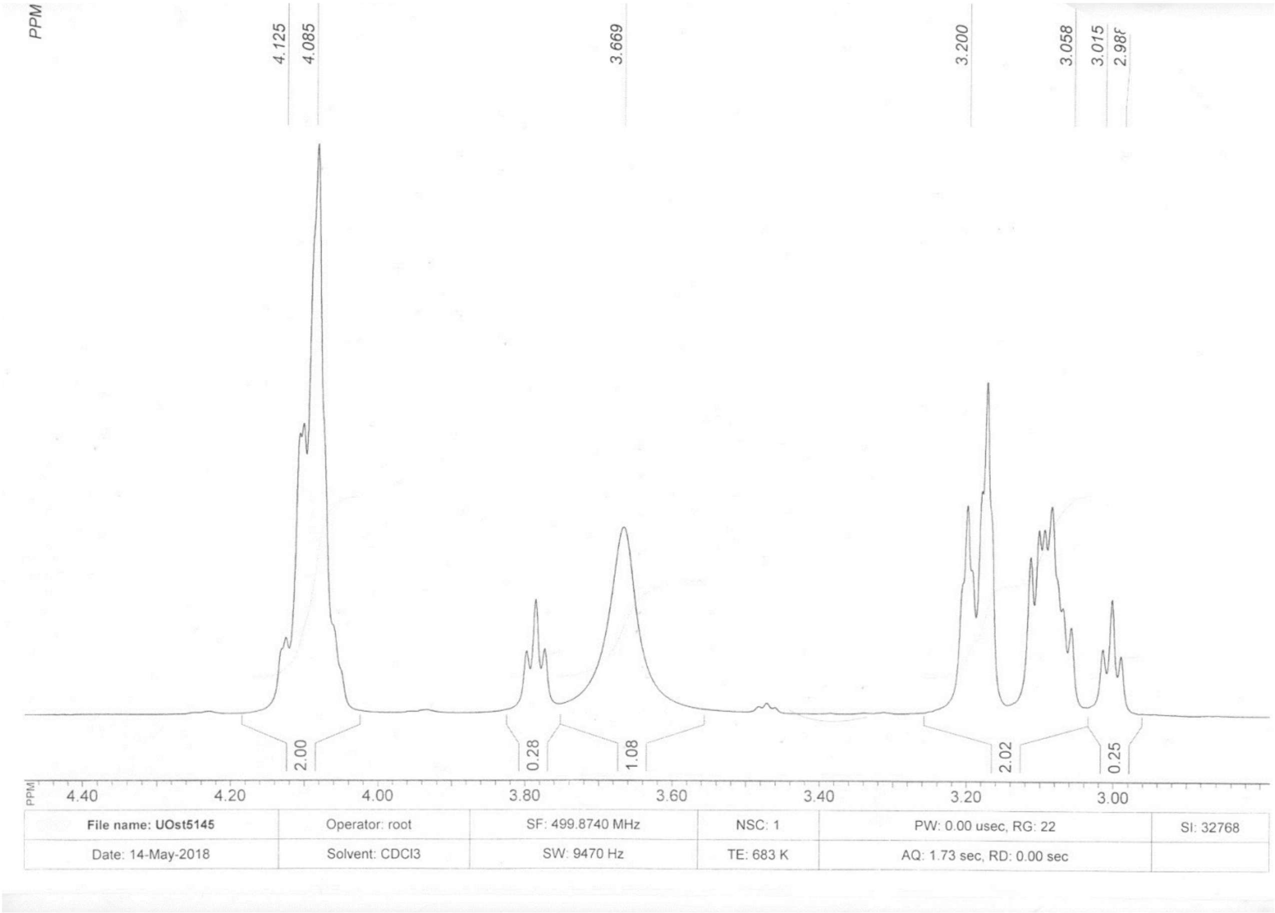
PPM
4.125
4.085
3.669
3.200
3.058
3.015
2.98F
2.00
0.28
1.08
2.02
0.25
4.40 4.20 4.00 3.80 3.60 3.40 3.20 3.00
File name: UOst5145
Operator: root
SF: 499.8740 MHz
NSC: 1
PW: 0.00 usec, RG: 22
SI: 32768
Date: 14-May-2018
Solvent: CDCl3
SW: 9470 Hz
TE: 683 K
AQ: 1.73 sec, RD: 0.00 sec

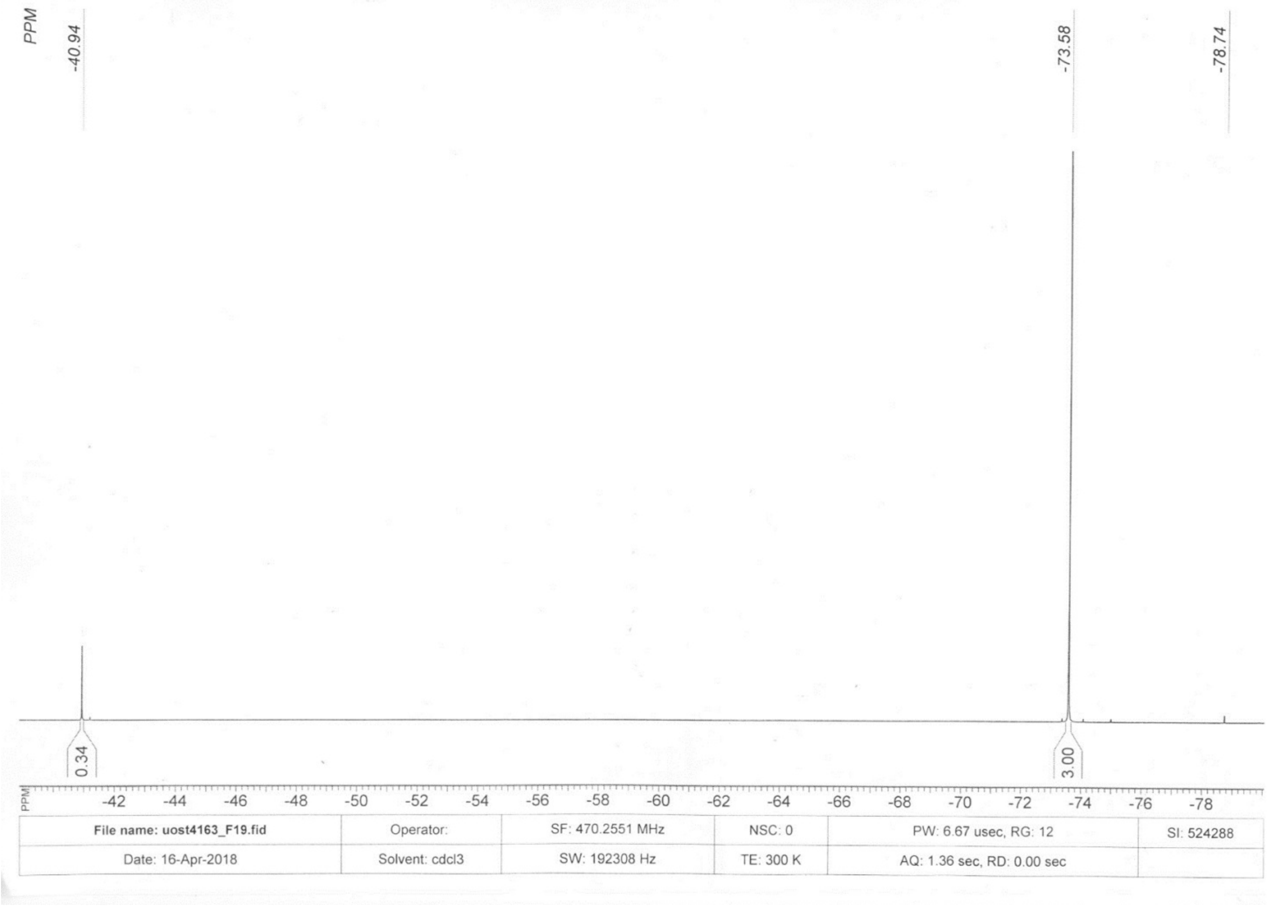
PPM
-40.94
-73.58
-78.74
0.34
3.00
-42 -44 -46 -48 -50 -52 -54 -56 -58 -60 -62 -64 -66 -68 -70 -72 -74 -76 -78
File name: uost4163_F19.fid
Operator:
SF: 470.2551 MHz
NSC: 0
PW: 6.67 usec, RG: 12
SI: 524288
Date: 16-Apr-2018
Solvent: cdcl3
SW: 192308 Hz
TE: 300 K
AQ: 1.36 sec, RD: 0.00 sec

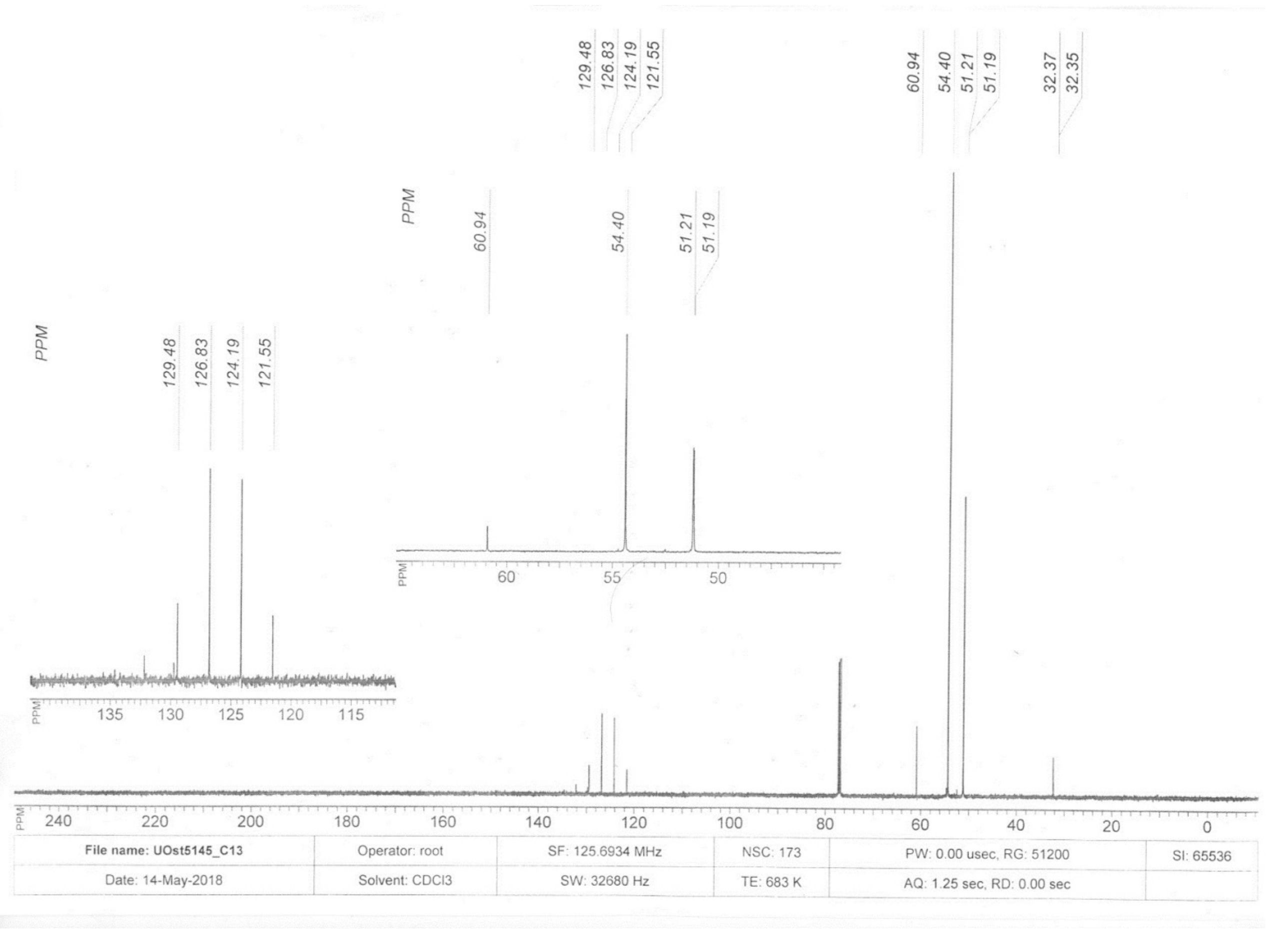
129.48
126.83
124.19
121.55
60.94
54.40
51.21
51.19
32.37
32.35
PPM
File name: UOst5145_C13
Date: 14-May-2018
Operator: root
Solvent: CDCl3
SF: 125.6934 MHz
SW: 32680 Hz
NSC: 173
TE: 683 K
PW: 0.00 usec, RG: 51200
AQ: 1.25 sec, RD: 0.00 sec
SI: 65536

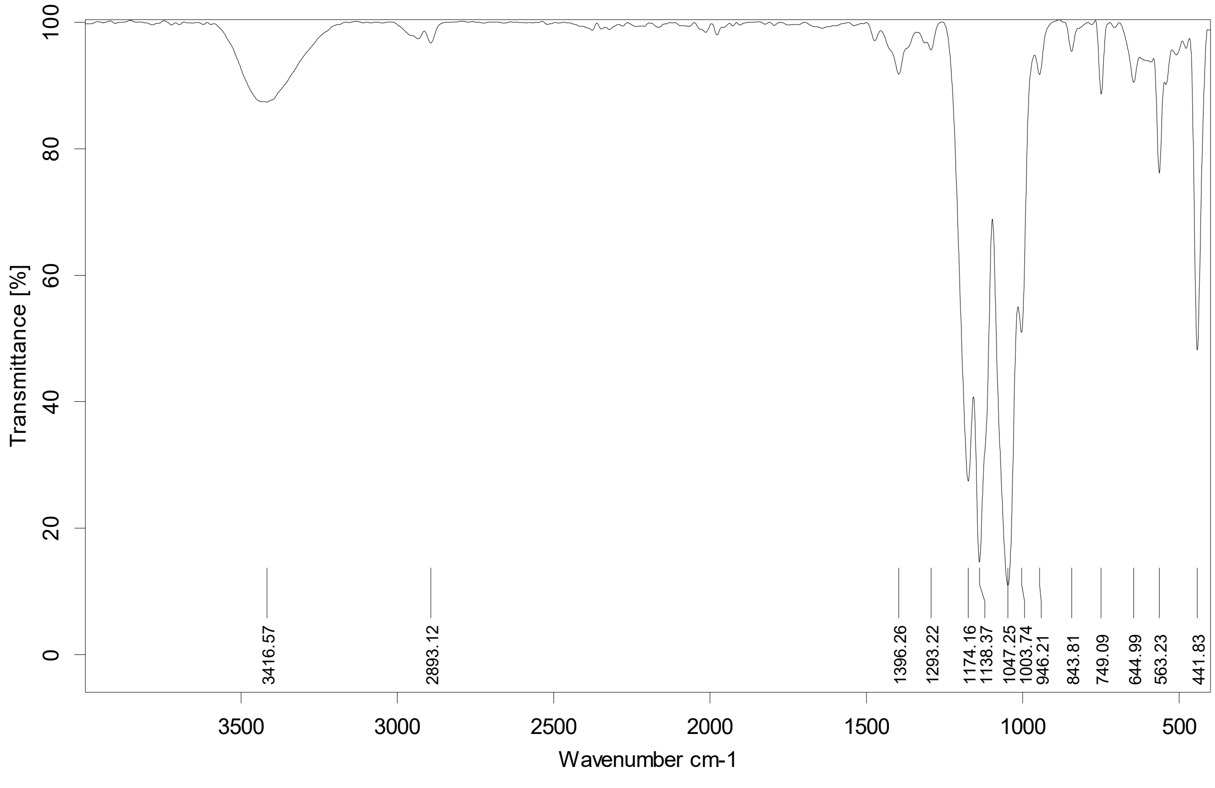
Transmittance [%]
Wavenumber cm-1
3416.57
2893.12
1396.26
1293.22
1174.16
1138.37
1047.25
1003.74
946.21
843.81
749.09
644.99
563.23
441.83

2-((Trifluoromethyl)sulfinyl)ethyl acetate.

Product after distillation. Contains ~15 % of corresponding alcohol.

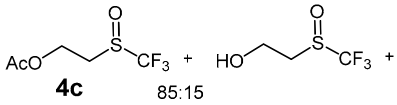

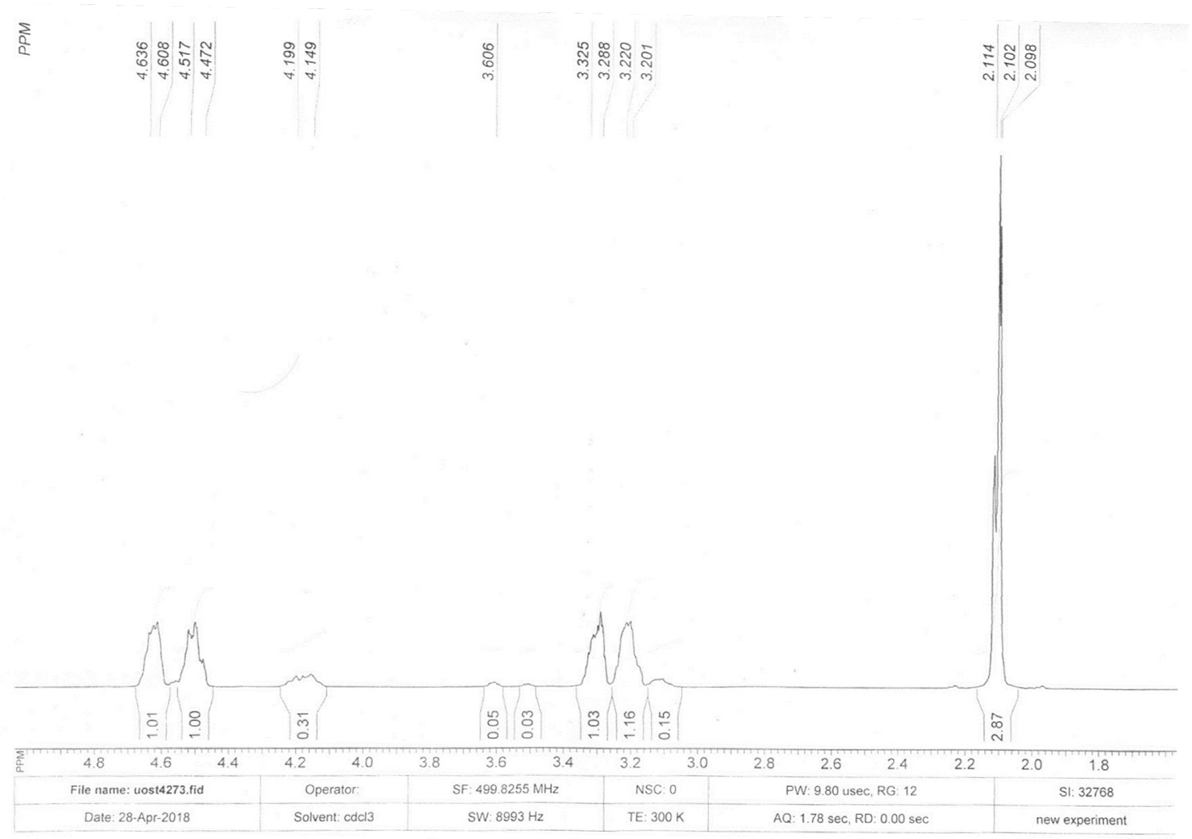

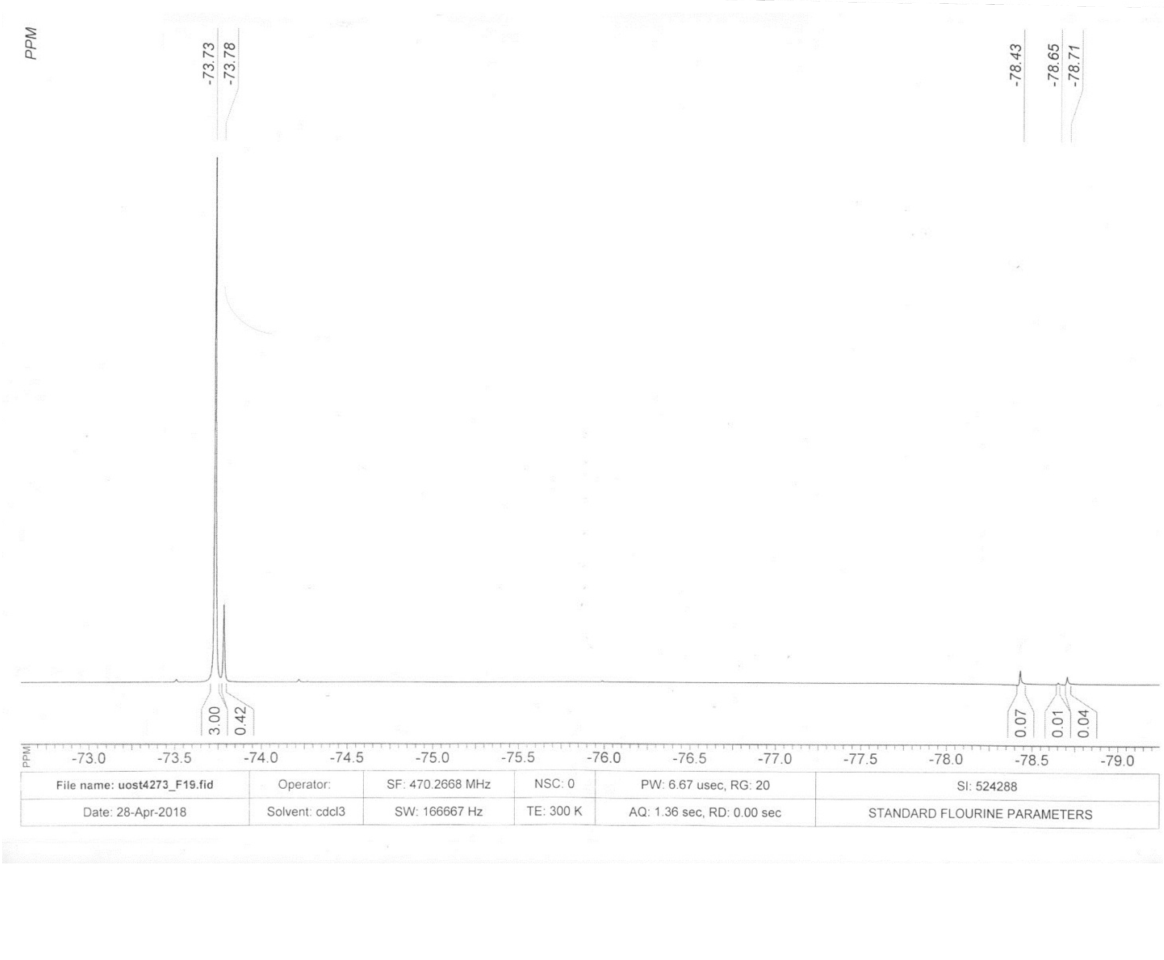

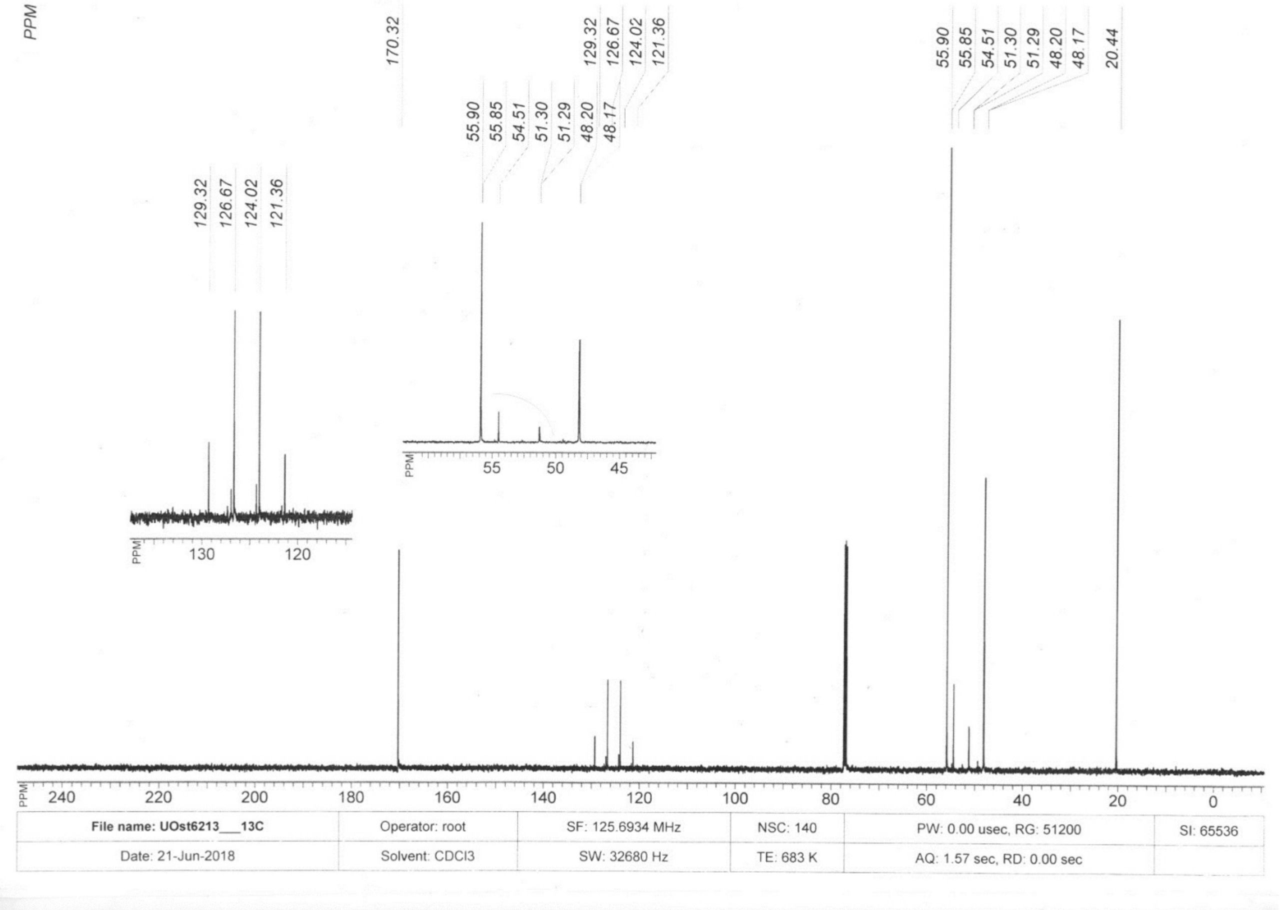

Methyl 3-((trifluoromethyl)sulfinyl)propanoate.

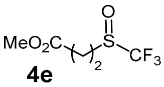

4e

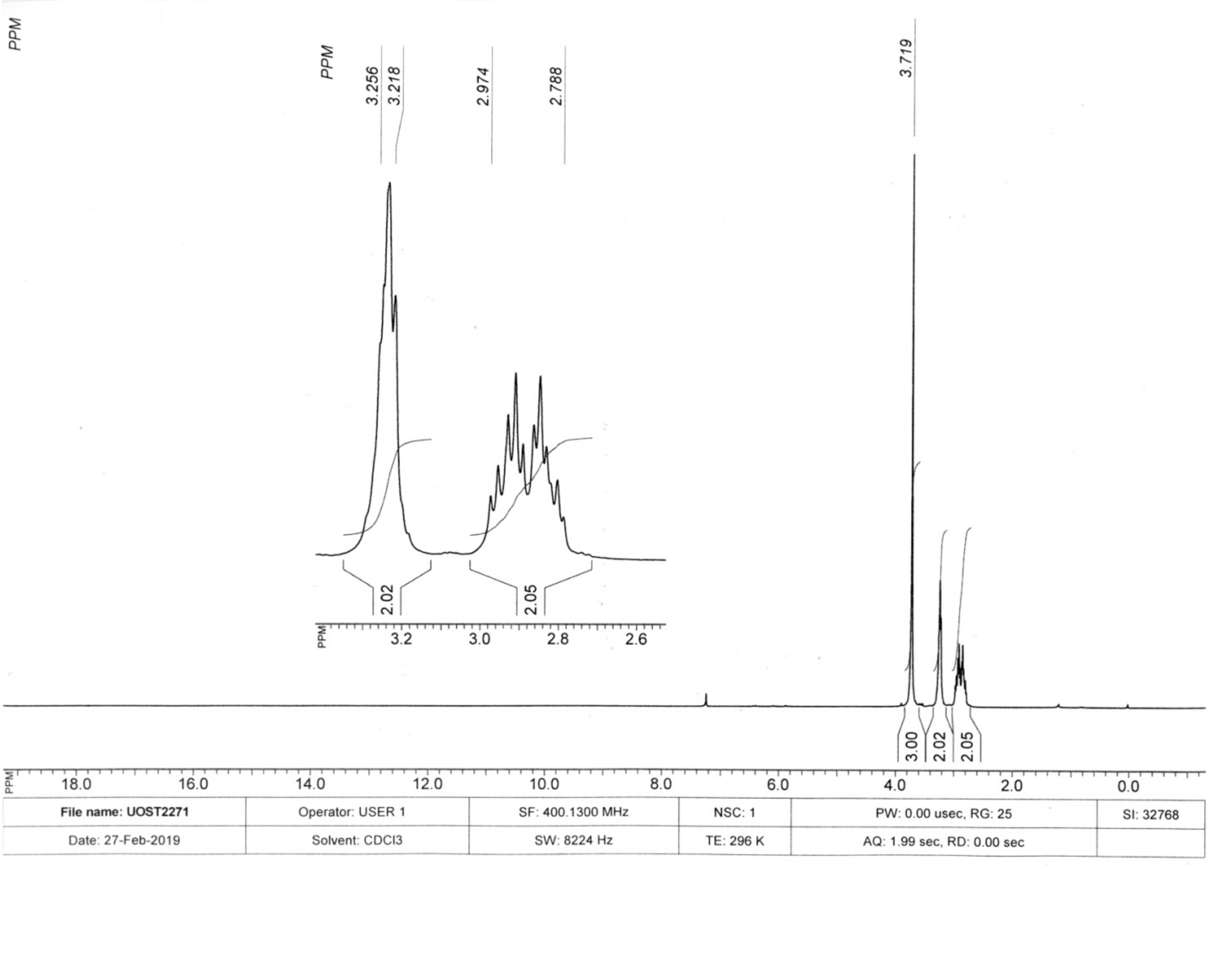

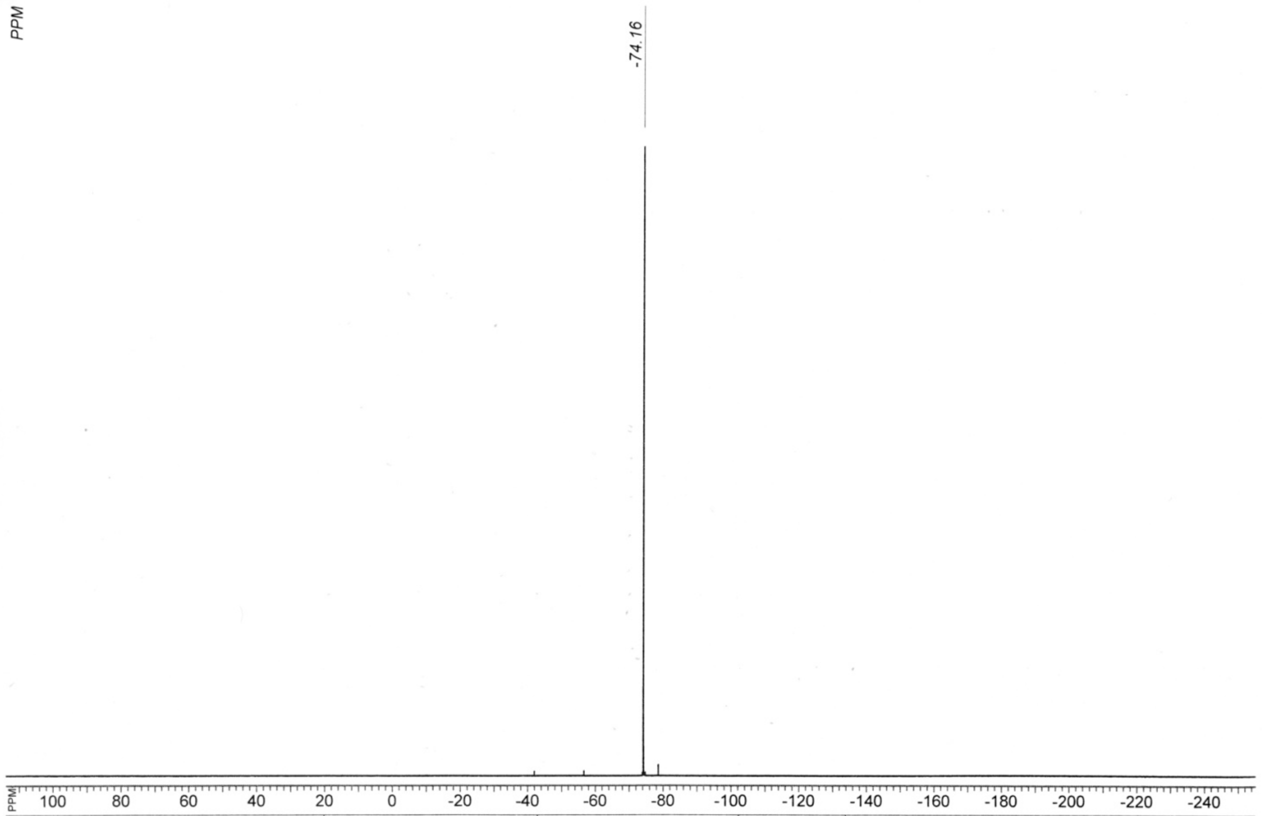

File name: UOST2273_F19{H}	Operator: USER 1	SF: 376.4986 MHz	NSC: 2	PW: 0.00 usec, RG: 1030	SI: 262144
Date: 27-Feb-2019	Solvent: CDCl3	SW: 138889 Hz	TE: 296 K	AQ: 0.94 sec, RD: 0.00 sec	

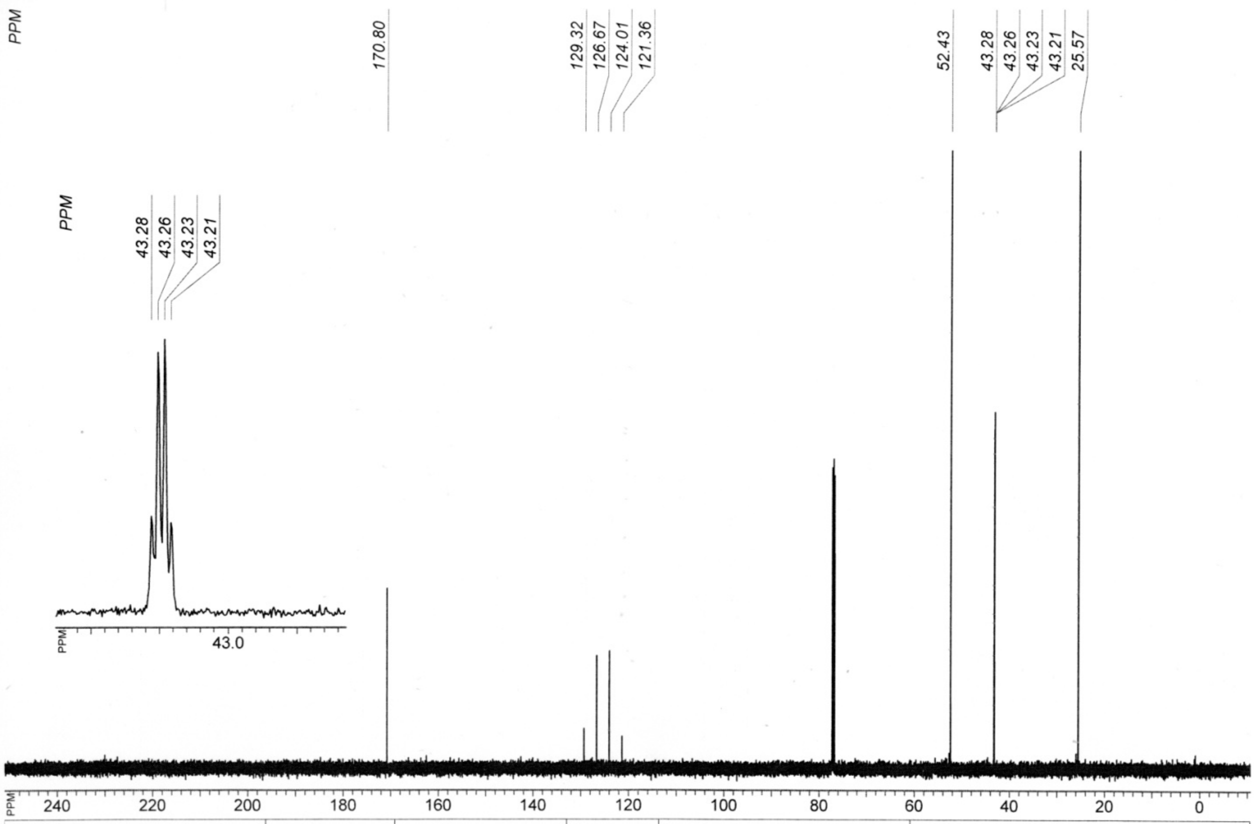

File name: uost2273_C13.fid	Operator:	SF: 125.6953 MHz	NSC: 0	PW: 3.33 usec, RG: 60	SI: 65536
Date: 28-Feb-2019	Solvent: cdcl3	SW: 32895 Hz	TE: 323 K	AQ: 0.97 sec, RD: 0.00 sec	Automated Probe tuning parameter

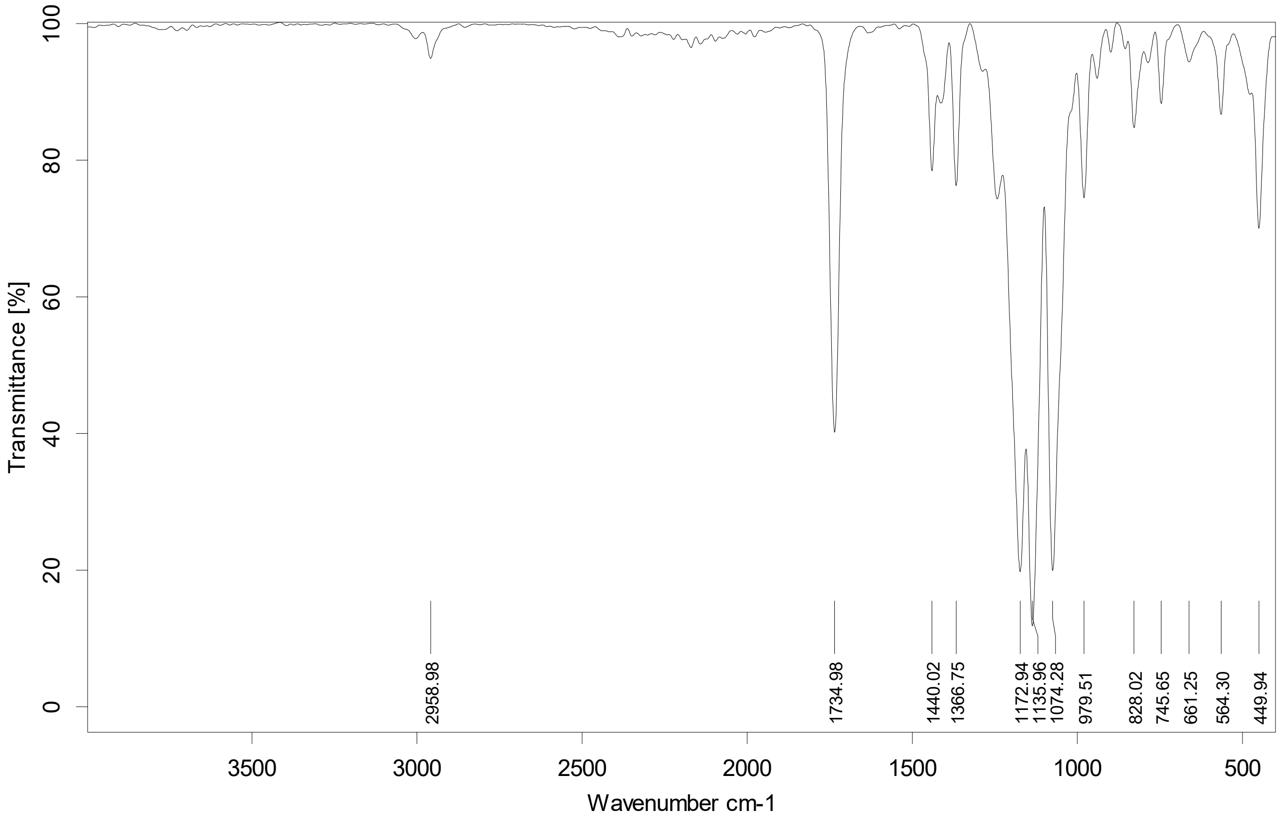

7-((Trifluoromethyl)sulfinyl)heptanenitrile 4f.

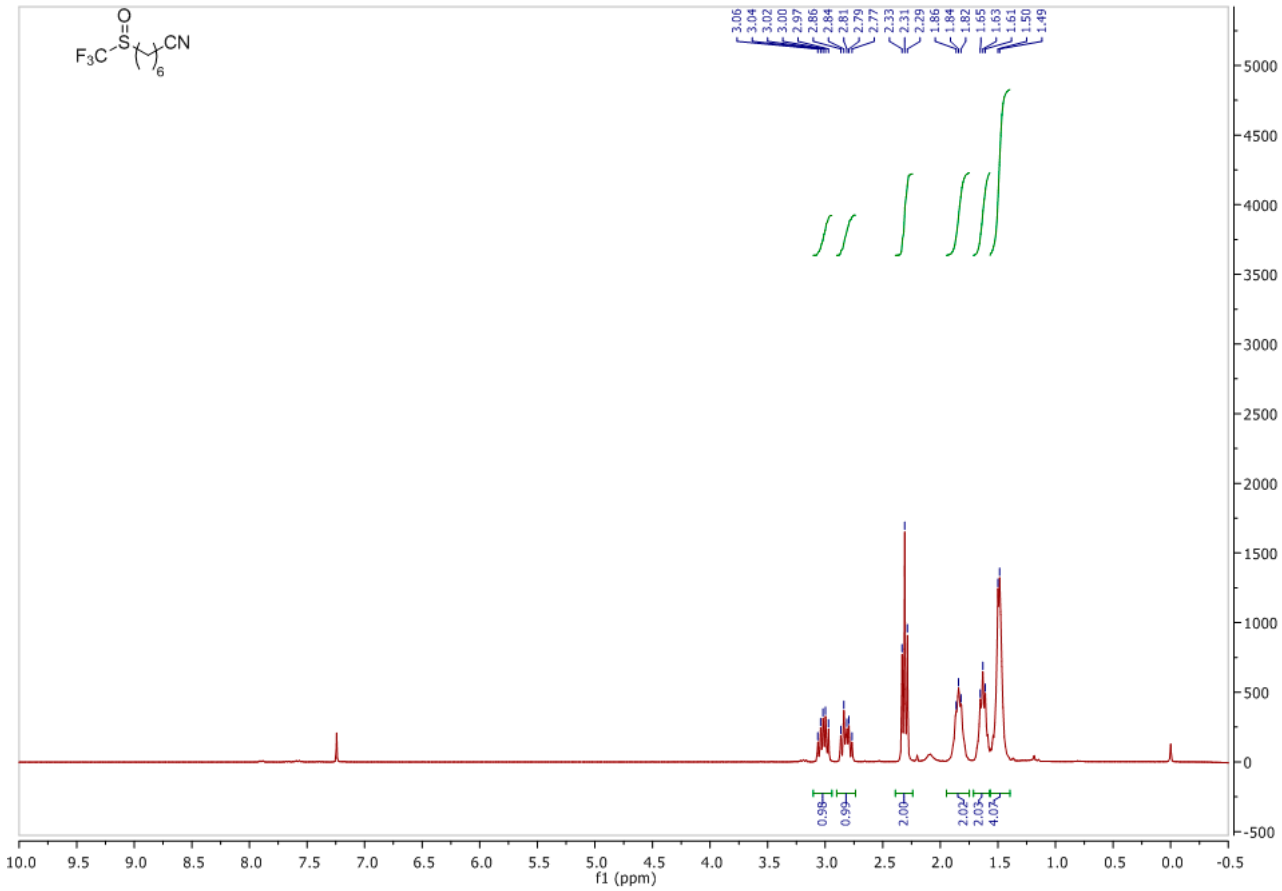

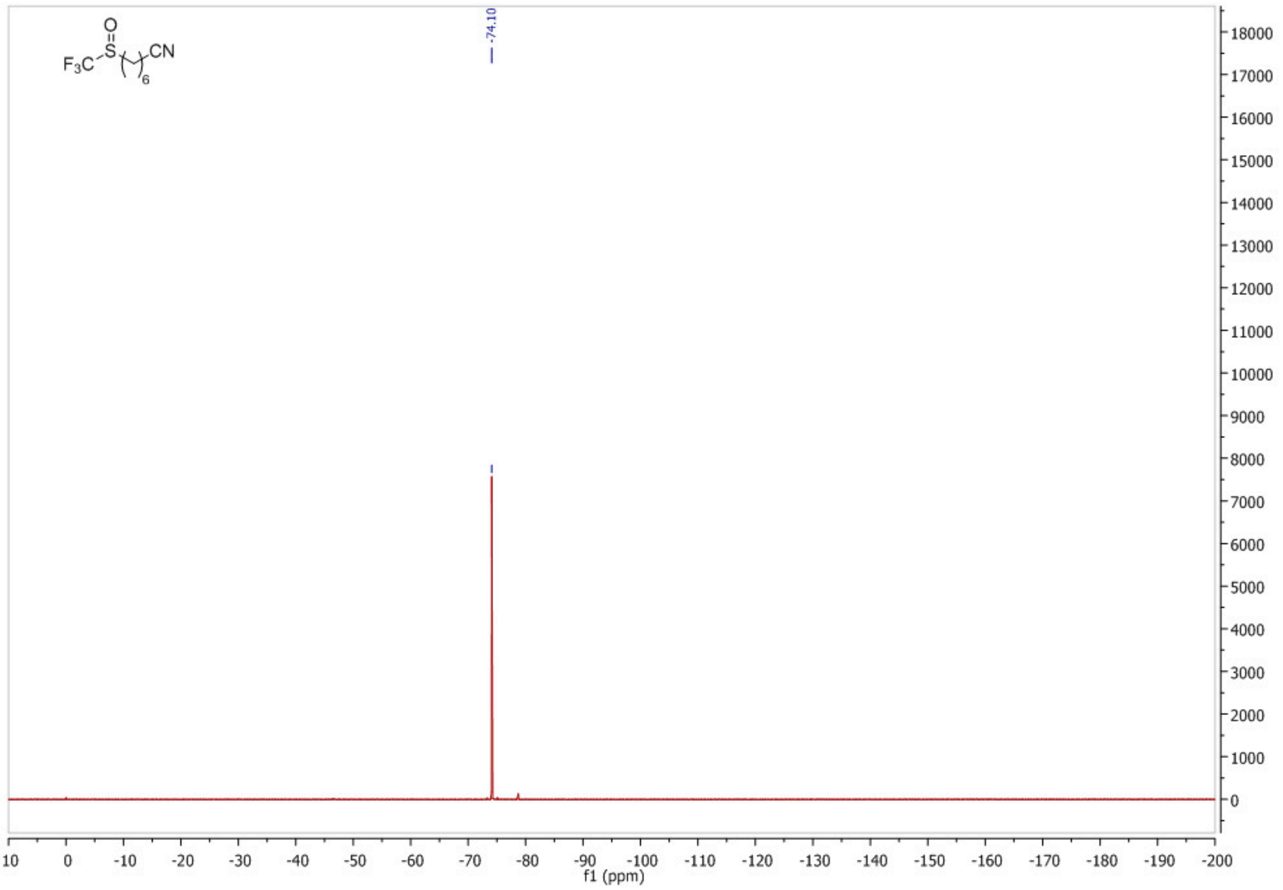

F3C S CN
6
-74.10
f1 (ppm)

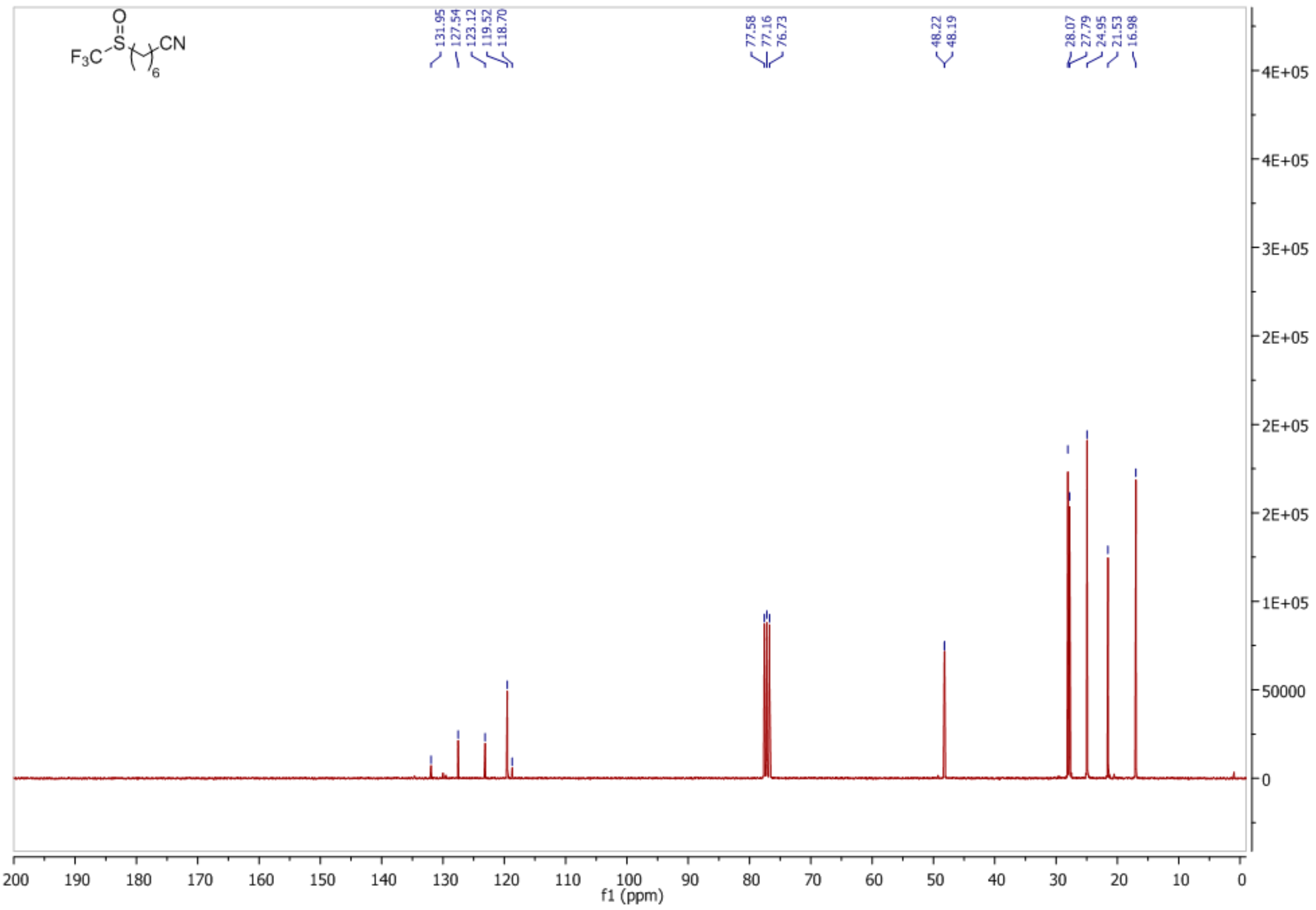

F3C S CN
6
131.95
127.54
123.12
119.52
118.70
77.58
77.16
76.73
48.22
48.19
28.07
27.79
24.95
21.53
16.98
f1 (ppm)

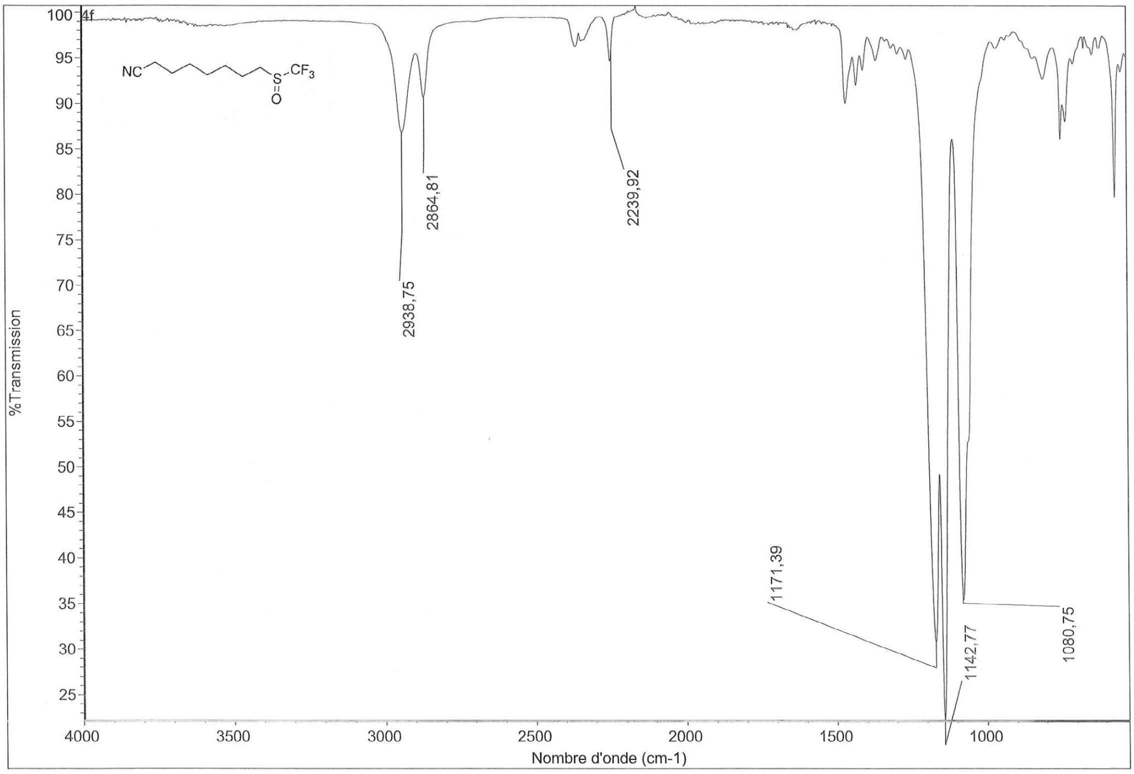

(1,1,2,2-Tetrafluoro-2-((1,2,4-triazol)-1-yl)-ethyl)-sulfinylbenzene 6d.

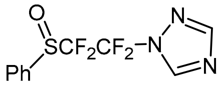

6d

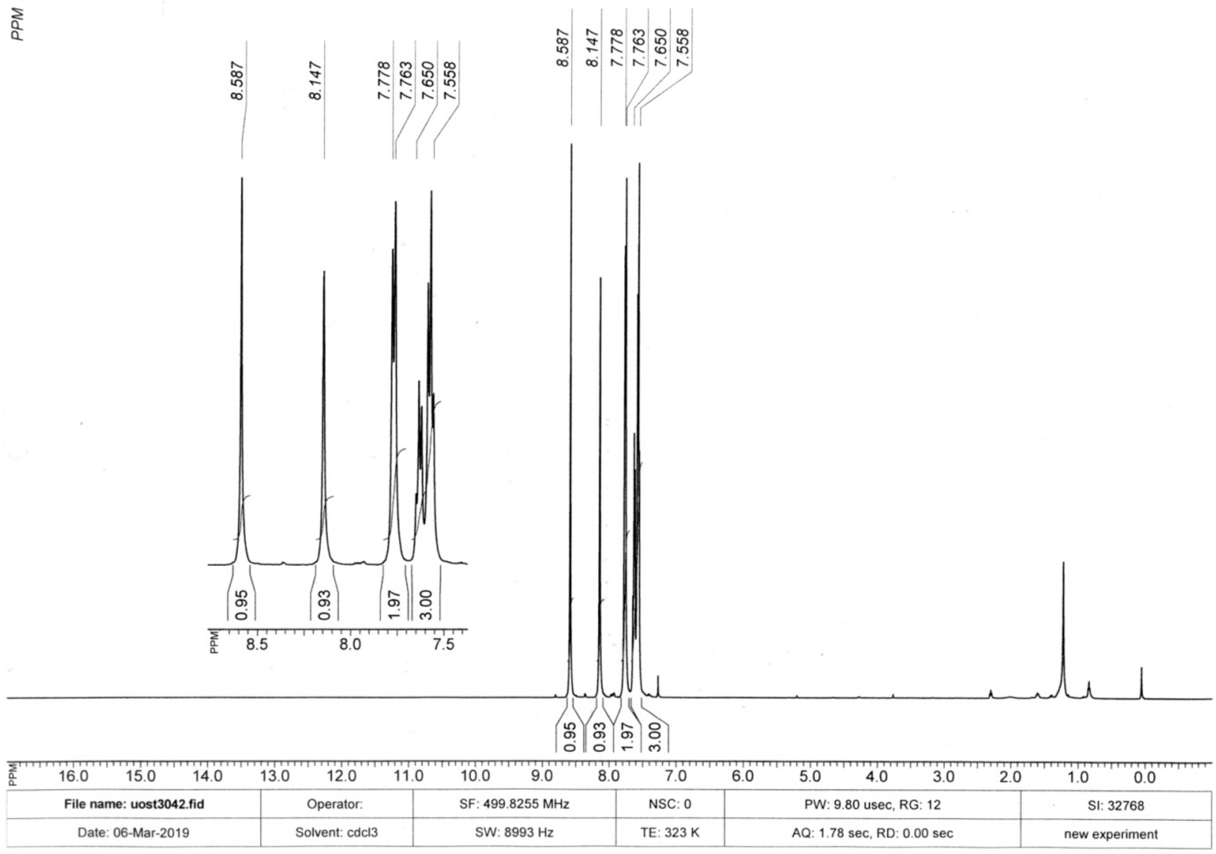

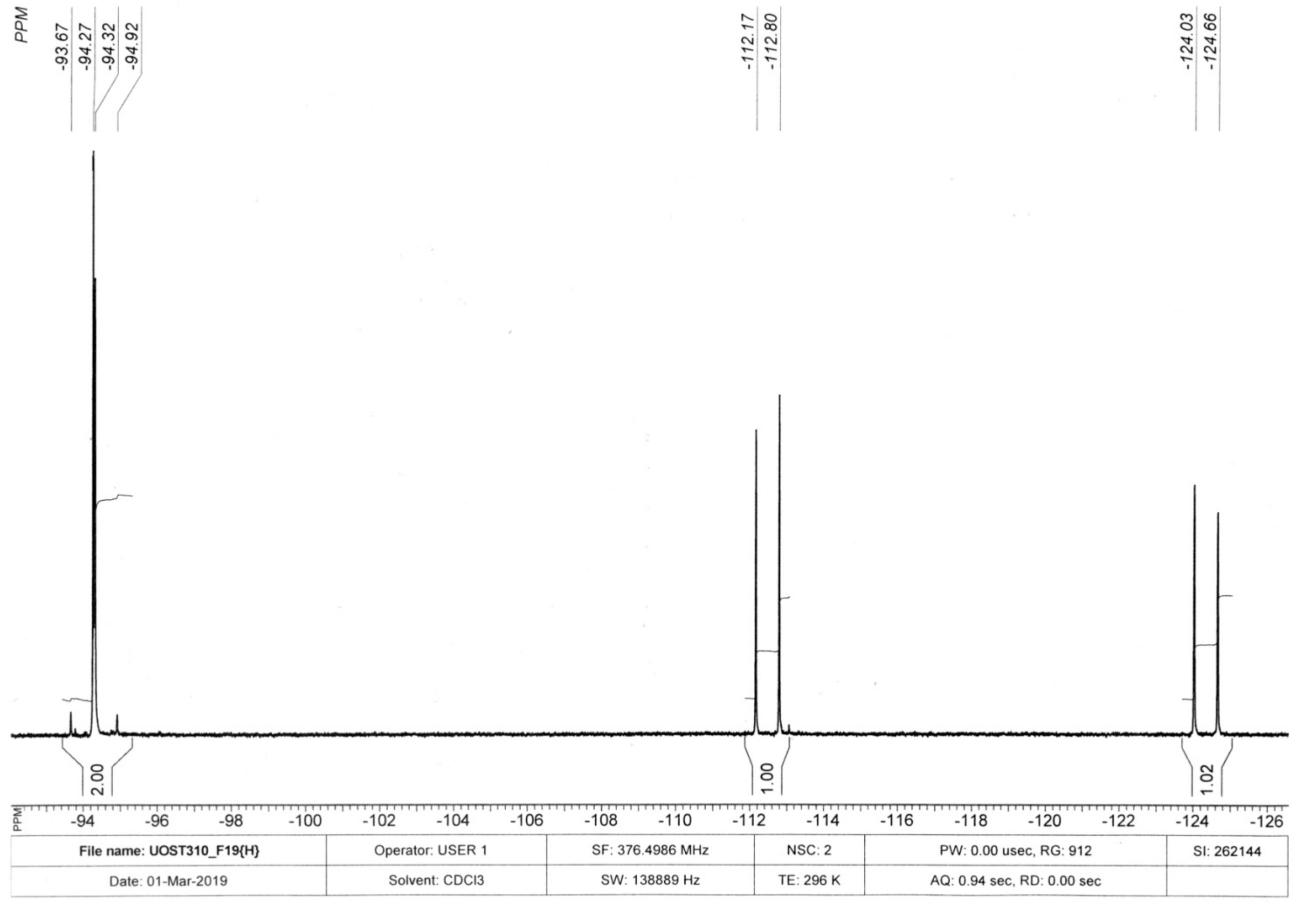
PPM
-93.67
-94.27
-94.32
-94.92
-112.17
-112.80
-124.03
-124.66
2.00
1.00
1.02
-94 -96 -98 -100 -102 -104 -106 -108 -110 -112 -114 -116 -118 -120 -122 -124 -126
File name: UOST310_F19{H}
Operator: USER 1
SF: 376.4986 MHz
NSC: 2
PW: 0.00 usec, RG: 912
SI: 262144
Date: 01-Mar-2019
Solvent: CDCl3
SW: 138889 Hz
TE: 296 K
AQ: 0.94 sec, RD: 0.00 sec

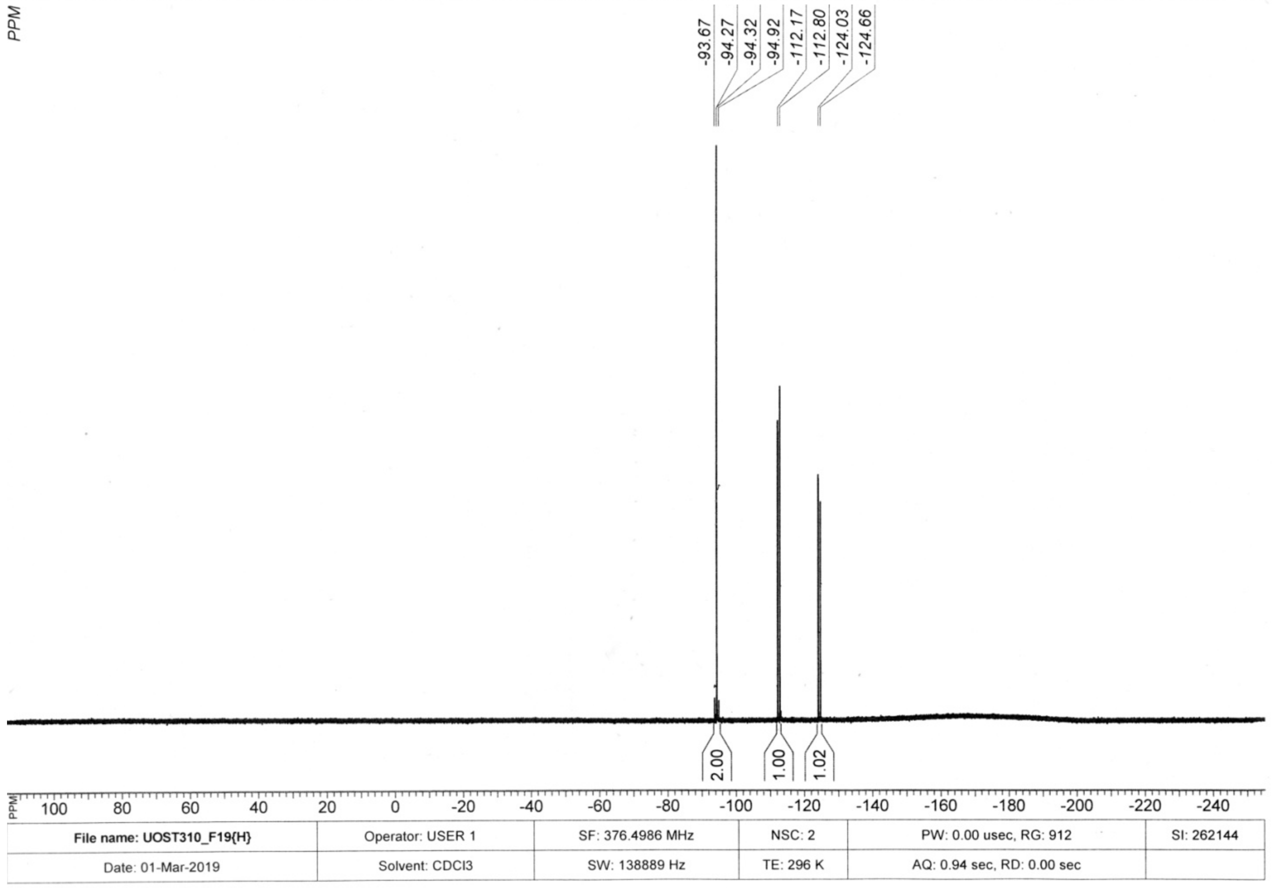
PPM
-93.67
-94.27
-94.32
-94.92
-112.17
-112.80
-124.03
-124.66
2.00
1.00
1.02
100 80 60 40 20 0 -20 -40 -60 -80 -100 -120 -140 -160 -180 -200 -220 -240
File name: UOST310_F19{H}
Operator: USER 1
SF: 376.4986 MHz
NSC: 2
PW: 0.00 usec, RG: 912
SI: 262144
Date: 01-Mar-2019
Solvent: CDCl3
SW: 138889 Hz
TE: 296 K
AQ: 0.94 sec, RD: 0.00 sec

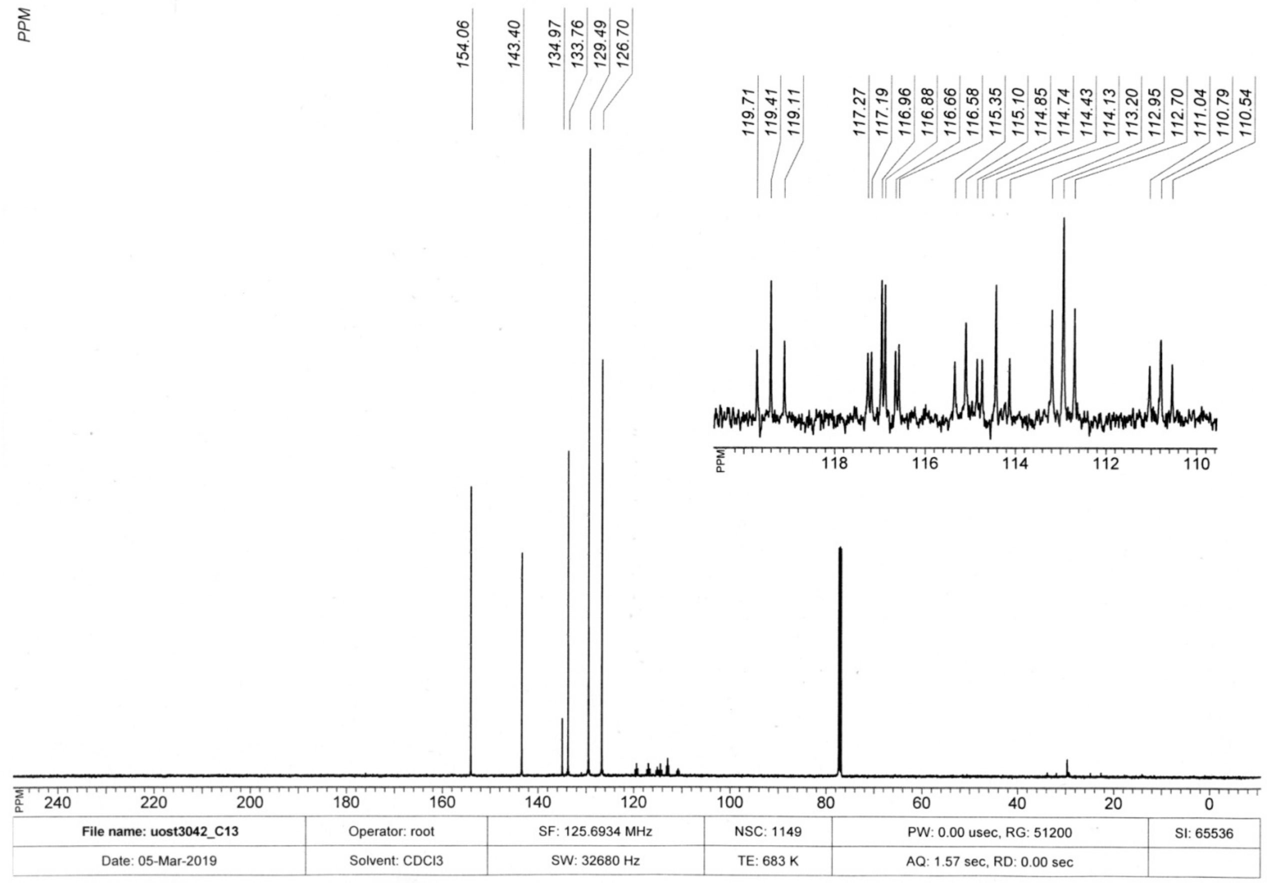
PPM
154.06
143.40
134.97
133.76
129.49
126.70
119.71
119.41
119.11
117.27
117.19
116.96
116.88
116.66
116.58
115.35
115.10
114.85
114.74
114.43
114.13
113.20
112.95
112.70
111.04
110.79
110.54
File name: uost3042_C13
Operator: root
SF: 125.6934 MHz
NSC: 1149
PW: 0.00 usec, RG: 51200
SI: 65536
Date: 05-Mar-2019
Solvent: CDCl3
SW: 32680 Hz
TE: 683 K
AQ: 1.57 sec, RD: 0.00 sec

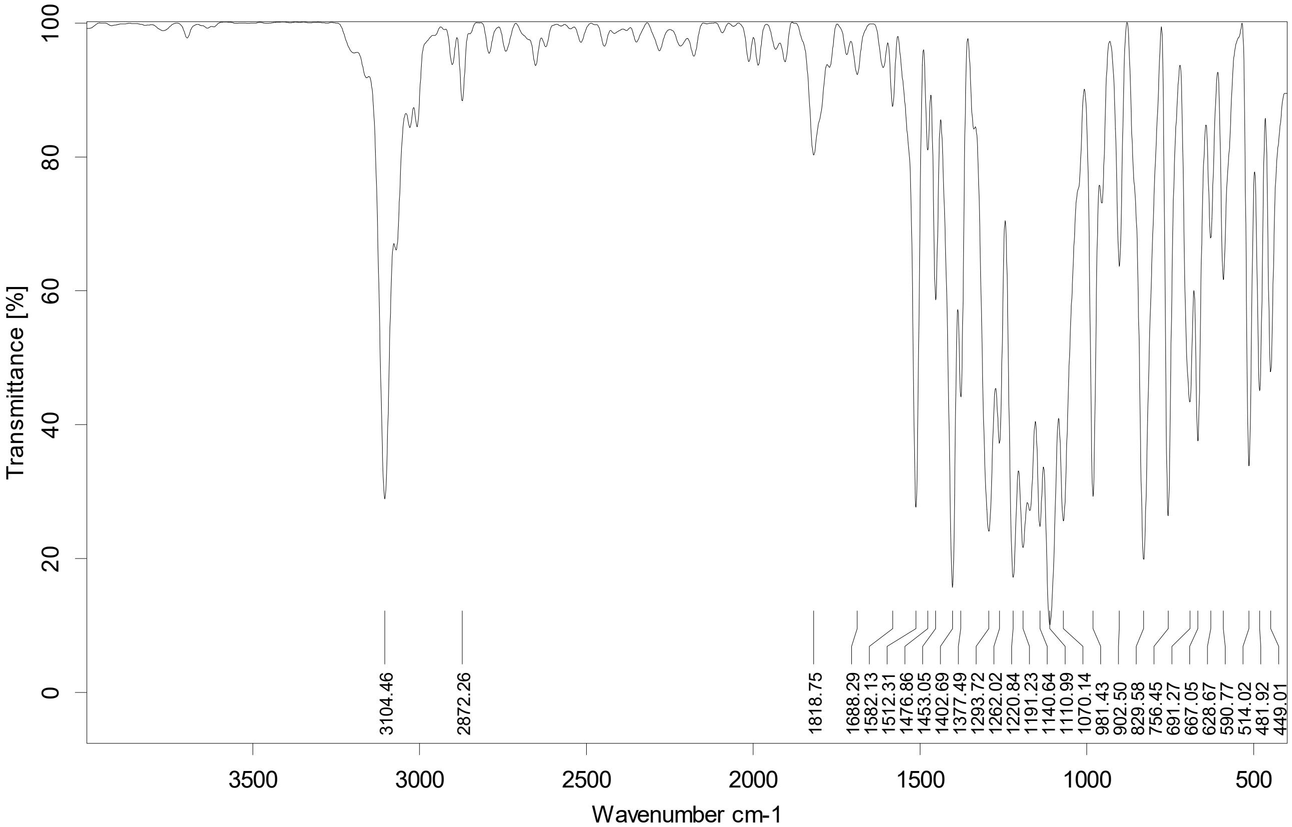
Transmittance [%]
Wavenumber cm-1
3104.46
2872.26
1818.75
1688.29
1582.13
1512.31
1476.86
1453.05
1402.69
1377.49
1293.72
1262.02
1220.84
1191.23
1140.64
1110.99
1070.14
981.43
902.50
829.58
756.45
691.27
667.05
628.67
590.77
514.02
481.92
449.01

N-(6-((Trifluoromethyl)sulfinyl)pyridin-3-yl)acetamide 8.

Product with ~80 % purity. Contain ~20% of starting sulfide.

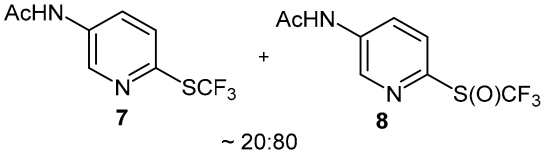

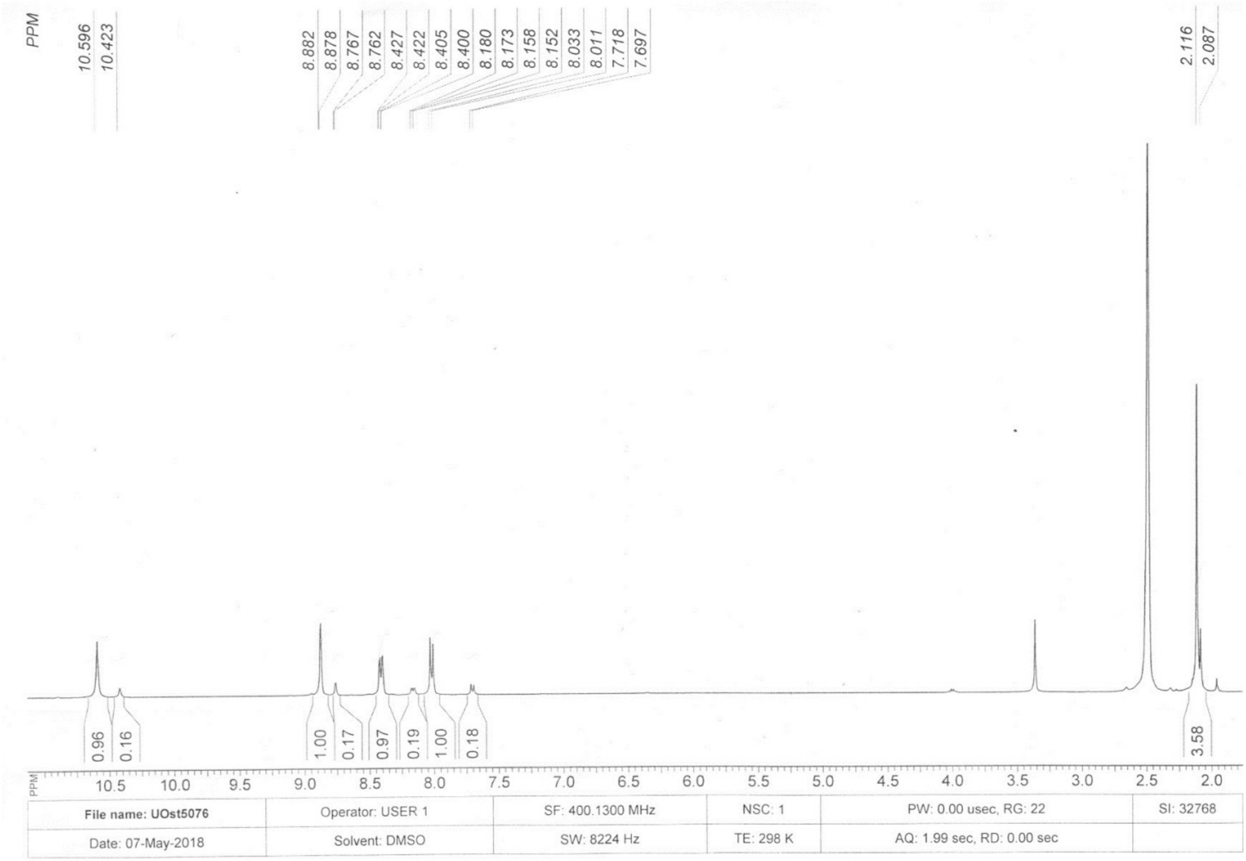

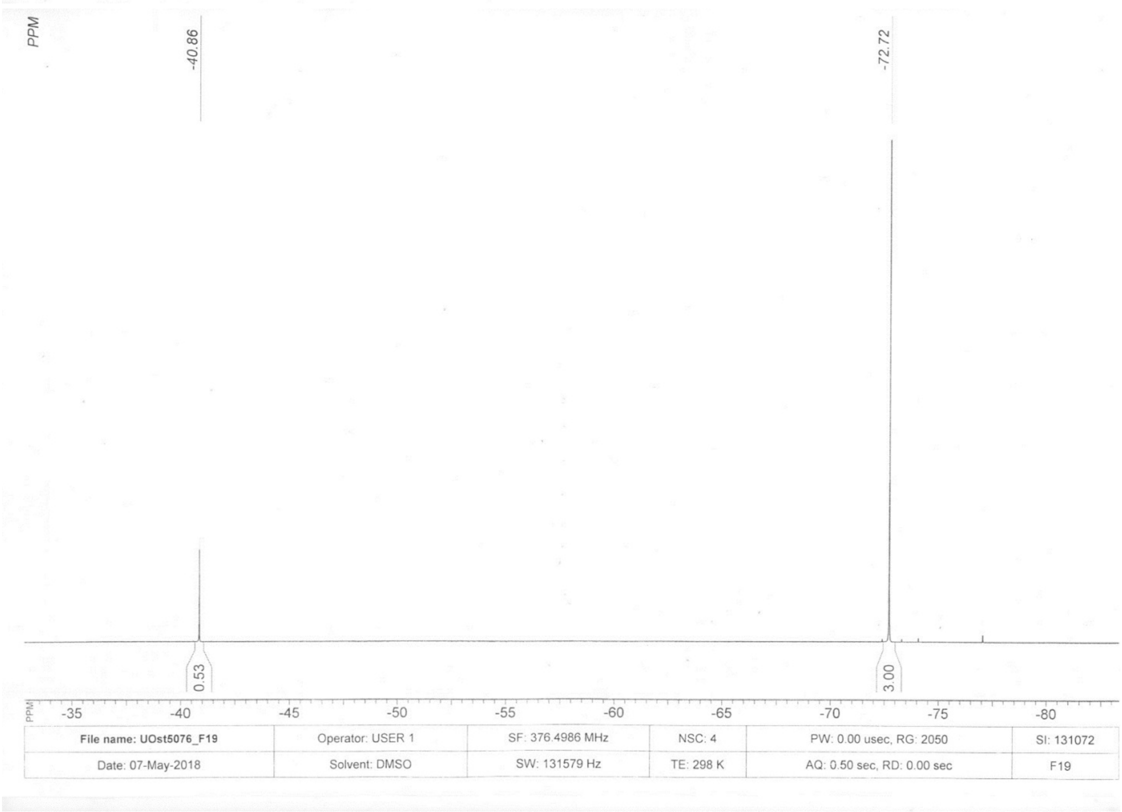

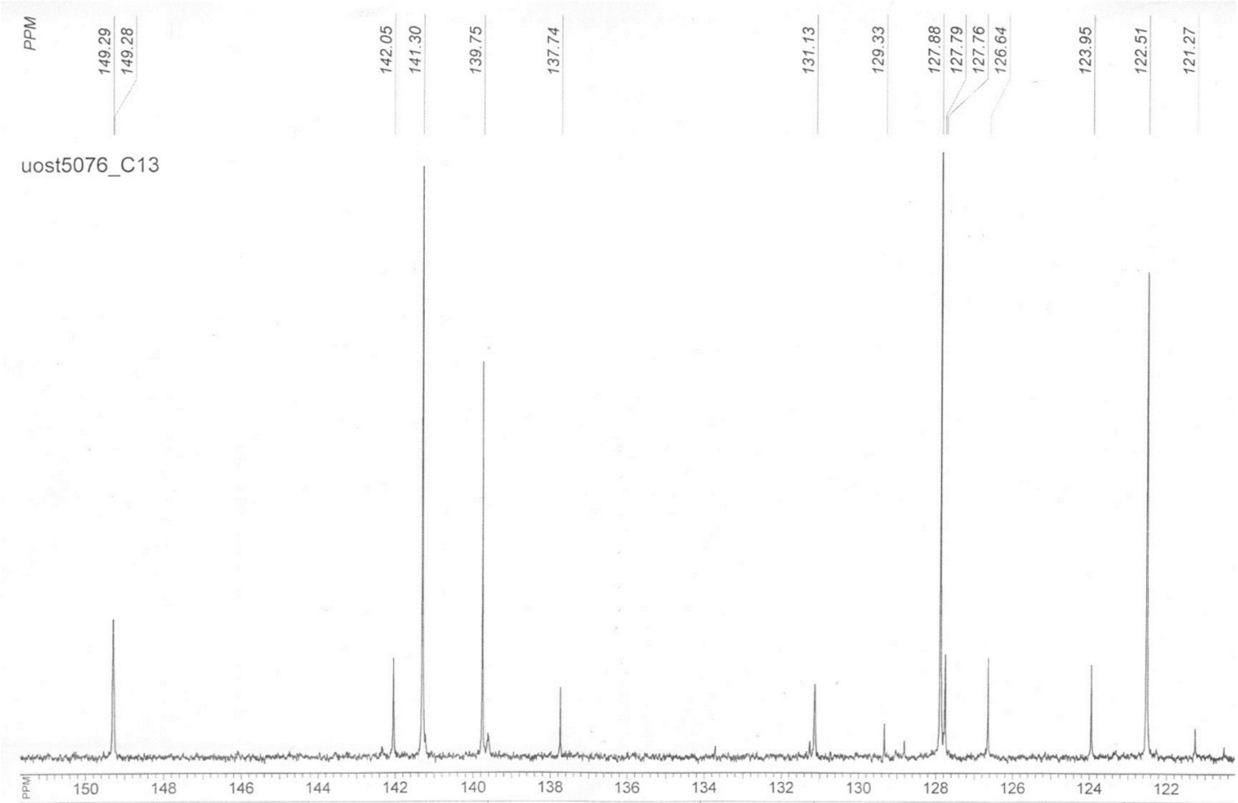

**File name: uost5076_C13**	Operator: root	SF: 125.6934 MHz	NSC: 563	PW: 0.00 usec, RG: 51200	SI: 65536
Date: 09-May-2018	Solvent: CDCl3	SW: 32680 Hz	TE: 683 K	AQ: 1.57 sec, RD: 0.00 sec	

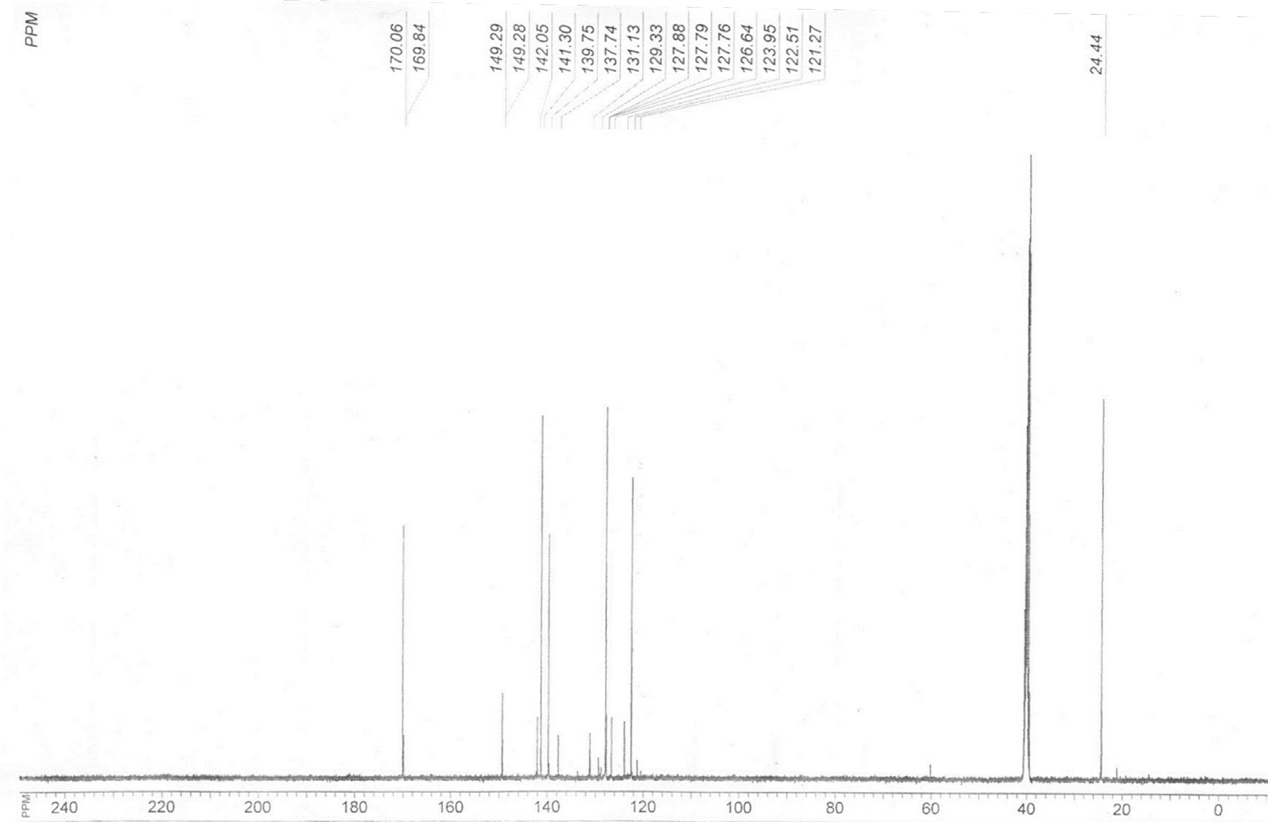

**File name: uost5076_C13**	Operator: root	SF: 125.6934 MHz	NSC: 563	PW: 0.00 usec, RG: 51200	SI: 65536
Date: 09-May-2018	Solvent: CDCl3	SW: 32680 Hz	TE: 683 K	AQ: 1.57 sec, RD: 0.00 sec	

Reaction mixture of the oxidation reaction for compound 9.

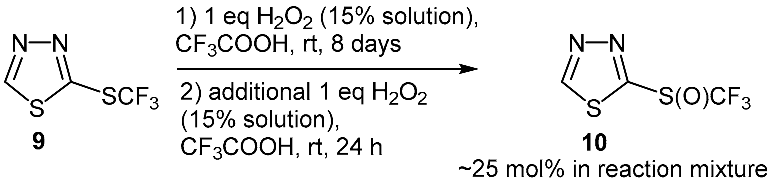

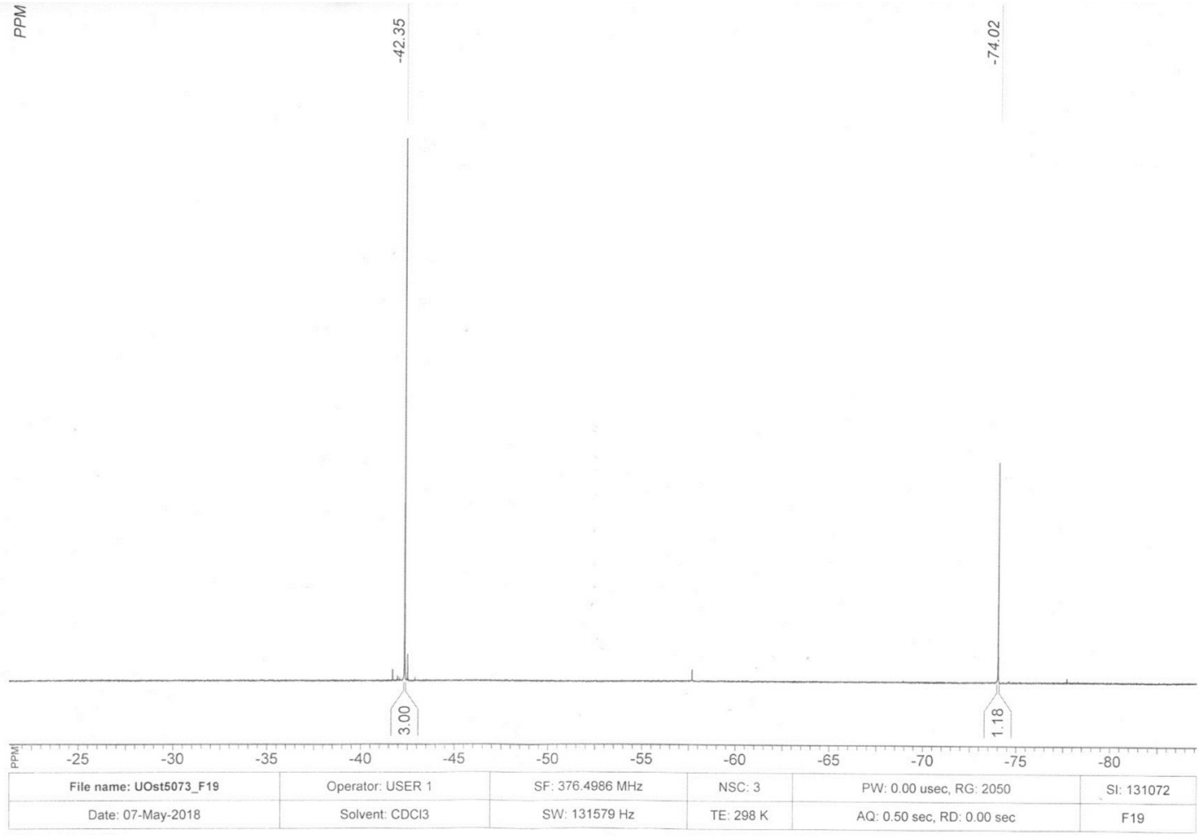

Supplement: Supplementary file 1 [file molecules-24-01249-s001.pdf]
